# YAP1 converts circELP2-mediated biochemical signals to mechanical forces through promoting cytoskeleton remodeling in pulmonary fibrosis

**DOI:** 10.7150/ijbs.127193

**Published:** 2026-04-08

**Authors:** Ruiqiong Li, Songzi Zhang, Jinjin Zhang, Haitong Zhang, Yujie Wang, Changjun Lv, Bo Liu, Lihua Ren, Xiaodong Song, Fang Wang

**Affiliations:** 1 College of Basic Medical Sciences, Jilin University, Changchun 130021, China.; 2 School of Nursing, Peking University, Beijing 100191, China.; 3 Shandong Key Lab of Complex Medical Intelligence and Aging, Yantai 264003, China.; 4 Department of Cellular and Genetic Medicine, Binzhou Medical University, Yantai 264003, China.; 5 Department of Respiratory and Critical Care Medicine, Binzhou Medical University Hospital, Binzhou Medical University, Binzhou 256603, China.

**Keywords:** pulmonary fibrosis, phase separation, circRNA, cytoskeleton remodeling, super-enhancer

## Abstract

Idiopathic pulmonary fibrosis (IPF) is a fatal interstitial lung disease with unknown mechanism and without effective drugs. The mechanisms involved in the progression of IPF are multifactorial, among which the conversion of biochemical signal to mechanical force has not been studied. Investigating the mechanism underlying IPF pathogenesis is essential for developing therapeutic approaches. The study aimed to investigate the mechanism of circELP2-mediated mechanical forces through YAP1. Mechanistic dissection clarified that circELP2 binds with the 217-307 amino acid fragment of TRIM25 to promote TRIM25 deacetylation at K447 site, which facilitates phase separation formation of TRIM25 via its 370-401 amino acid fragment. Then, TRIM25 strengthens ubiquitin-dependent degradation of 14-3-3ζ, which results in the target gene YAP1 of 14-3-3ζ translocation from the cytoplasm to the nucleus. Nuclear YAP1 drives the super-enhancer formation on the cytoskeleton gene locus to initiates the transcription of cytoskeleton remodeling genes, leading to amplifying mechanical forces. Consequently, the condition accelerates fibroblast-to-myofibroblast differentiation to promote pulmonary fibrosis. These findings confirm that YAP1 converts circELP2-mediated biochemical signals to mechanical forces through promoting cytoskeleton remodeling in pulmonary fibrosis. Targeting circELP2 can mitigate pulmonary fibrogenesis which is a mechanical force-related therapeutic target for IPF.

## Introduction

Idiopathic pulmonary fibrosis (IPF) is a fatal interstitial lung disease involving multiple cell types. Recurrent injury to alveolar epithelial cells interacts with mesenchymal, immune, and endothelial cells, triggering the differentiation of fibroblasts into myofibroblasts, followed by their proliferation and migration, as well as the deposition of extracellular matrix 1, 2. Typically, patients succumb to respiratory failure within three to five years after diagnosis. The incidence of IPF varies significantly based on geographic region, case definitions, and population demographics, with a notable increase in prevalence associated with advancing age globally 3, 4. Additionally, post-acute sequelae of coronavirus disease 2019 (COVID-19) may affect 10-30% of COVID-19 survivors, with post-acute sequelae of COVID-19-associated pulmonary fibrosis emerging as a long-term outcome with significant morbidity 5. Currently, the efficacy of available pharmacotherapies for IPF is limited, often accompanied by various side effects such as photosensitivity, skin rash, upper gastrointestinal symptoms and liver toxicity 6, 7. Therefore, elucidating the mechanisms underlying IPF pathogenesis is vital for exploring novel therapeutic approaches.

The mechanisms involved in the progression of IPF are multifactorial, among which the increased transmission between mechanical forces and biochemical signals within lung tissue being particularly underexplored. Mechanical forces, including mechanical stiffness, tension, stretch, adhesion, and density, are essential for maintaining organ structure and function, development, and overall normal physiological levels. These forces, in conjunction with biochemical signals, play a crucial role in guiding cellular processes such as differentiation, proliferation, migration, and death through mechano-transduction pathways 8, 9. Emerging evidence has shown that mechanical forces undergo significant alterations during the progression of pulmonary fibrosis 10. Wu *et al.* demonstrated that elevated mechanical tension activates a transforming growth factor-β1 (TGF-β1) signaling loop in alveolar type II cells, consequently driving the periphery-to-center progression of lung fibrosis 11. Cells are usually subjected to dynamic mechanical forces. These forces manifest as cellular stiffness, tension or stretch outside the cell, which are sensed by mechanosensitive molecules such as Piezo1, integrin and YAP/TAZ, and convert to the intracellular biological signals to regulate tissue repair, immune responses and cell fate 12-14. This mechanotransduction that mechanical forces convert to biochemical signals has received more attention. However, independent of outside-in mechanical forces, how intracellular biological signals convert to outside mechanical forces is missing 15, particularly during pulmonary fibrogenesis.

CircRNA is a covalently closed non-coding RNA lacking free 3' or 5' ends that regulates transcription, splicing, translation, and post-translational modification through interactions with DNA, RNA, and proteins, thereby participating in disease pathogenesis 16, 17. Therefore, it has emerged as a potential diagnostic marker and a target for therapeutic intervention 18-20. For instance, Roy *et al.* identified a panel of 8-circRNAs as non-invasive, liquid-biopsy biomarkers which can serve as potential diagnostic biomarkers for the early detection of gastric cancers 21. Guo *et al.* developed a therapeutic circRNA aptamer which can alleviate PKR-associated osteoarthritis 22. Therefore, exploration circRNA function and regulatory mechanism can provide new diagnostic biomarker and effective therapeutic target for disease.

Our previous studies identified that circELP2 was significantly highly expressed in plasma samples from IPF patients by high-throughput microarray assay 23. The cytoplasmic circELP2 can accelerate pulmonary fibrogenesis through targeting YAP1 by binding miRNA-630 24. Further study on IPF patients discovered that the lung samples of IPF patients exhibit cytoskeleton remodeling with a high tension or stiffness, which excites our interest. Yes-associated protein 1 (YAP1), a mechanotransducer, can read both biochemical signals and mechanical forces 25-27. Cytoskeleton can generate, transmit and respond to mechanical forces to affect cellular behaviour 28-30. Whether YAP1 converts circELP2-mediated biochemical signals to mechanical forces through promoting cytoskeleton remodeling in pulmonary fibrosis needs to be explored in depth. Multiple regulatory pathways can synergistically regulate the same gene, enhancing the precision and efficiency of expression. This enables cells to respond rapidly and robustly to weak signals while increasing their sensitivity to environmental changes. By studying these different signaling pathways, more precise treatment can be provided for patients. For these reasons, this article, a follow-up to previous research on circELP2 24, will try to address the three issues: one, whether circELP2 can be a biomarker for IPF; two, whether circELP2 can target YAP1 by its binding protein besides miRNA-630; three, whether circELP2 can convert biological signals to mechanical signals through YAP1 that lead to increased lung mechanical forces. This study first elucidated how the circELP2-mediated biochemical signals translate into mechanical forces, which not only provides a theoretical framework for understanding the mechanotransduction in pulmonary fibrosis, but also lays a scientific foundation for developing novel anti-fibrotic drugs targeting the circRNA-protein phase separation.

## Materials and Methods

### Human lung tissues

The acquisition of human samples was approved by the Medical Ethics Committee of Binzhou Medical University (approval number: 2023-125). All participants provided written informed consent prior to their enrolment. IPF patients, diagnosed in accordance with the American Thoracic Society and European Respiratory Society criteria for IPF. The age-matched healthy people and paracancerous lung tissues as controls were collected at the Affiliated Hospital of Binzhou Medical University (Binzhou and Yantai, China).

### MRC-5 cell culture and transfection

The human fetal lung fibroblasts medical research council cell strain-5 (MRC-5) cell line was purchased from the American Culture Collection of Typical Cultures (CCL-171™), incubated in Minimum Essential Medium (MEM, USA,) containing 1% glutamine, 1% non-essential amino acid, 1% pyruvic acid, and 10% fetal bovine serum and placed in an incubator at 37 °C and 5% CO_2_. MRC-5 cell treated with 5 ng/mL TGF-β1 (MCE, HY P70543) for 48 h was used as the cell model of pulmonary fibrosis. In the gain- and loss-of-function experiments, MRC-5 cells were treated with si-RNA (si-circELP2/si-TRIM25/si-YAP1) or overexpressed plasmid of circELP2, TRIM25, and YAP1 (over-circELP2/over-TRIM25/over-YAP1) for 6 h before TGF-β1 treatment for 48 h. In the rescue experiments, MRC-5 cells were treated with si-TRIM25/si-YAP1 for 6 h before the treatment of over-circELP2 for 48 h. The vector map of the circELP2 overexpression plasmid is listed in Figure S2 of the supplementary materials.

### Animal model

All animal experiments were approved by the Animal Ethics Committee of Binzhou Medical University (approval number: 2023-146). Eight-week-old C57BL/6J mice were purchased from the Pengyue Laboratory Animal Breeding Co., Ltd. (Jinan, China). All mice were bred and maintained in a 12 h light/dark cycle and allowed free access to food and water. The mice were divided into different groups (10 mice in each group) according to the experimental requirements. The Bleomycin (BLM)-treated mice were administered 5 mg/kg BLM, which was sprayed into the lungs using a Penn-Century MicroSprayer (PennCentury Inc., Wyndmoor, PA, USA). The sham group only received the same amount of normal saline as the control group. 1.0 × 10^12^ vg/mL circELP2, si-TRIM25/si-TRIM25 NC or si-YAP1/si-YAP1 NC (HANBIO, Shanghai) was packaged into the adeno-associated virus (AAV) and sprayed into the mouse lung according to the experimental requirements.

### RNA scope

RNA scope was performed to detect the expression of circELP2 in IPF patients by using BaseScope Reagent Kit v2-RED (Advanced Cell Diagnostics, USA). The fresh lung-tissue slices of IPF patients were baked at 60 °C for 60 min before deparaffinized and dehydrated with xylene and ethanol, respectively. After hydrogen peroxide was dropped on the slices, the slices were placed in target retrieval reagent at 98 °C for 15 min and incubated with protein IV at 40 °C for 30 min. circELP2 probe or positive control probe was added and incubated at 40 °C for 2 h. Then, the tissues were orderly treated with AMP1 (40 °C, 35 min), AMP2 (40 °C, 35 min), AMP3 (40 °C, 15 min), AMP4 (40 °C, 35 min), AMP5 (40 °C, 35 min), AMP6 (40 °C, 15 min), AMP7 (RT, 45 min), AMP8 (RT, 15 min). After coloration with chromogenic solution for 10 min, the tissues were stained with hematoxylin for 2 min. Finally, the tissues were sealed with vector mount sealer.

### RNA-binding protein immunoprecipitation (RIP)

RIP assays were performed using the RNA Immunoprecipitation Kit (GENESEED, China) according to the manufacturer's instructions. 1 × 10^7^ MRC-5 cells were transfected with the segmented plasmid of TRIM25 labeled with 3xFlag (TRIM25 MUT 3xFlag). Then, cell samples were lysed in lysis buffer containing protease inhibitors and RNase inhibitors. 100 μL of supernatant was taken as input control, and normal rabbit anti-IgG antibody (Cell Signaling Technology, USA) was used as negative control. Anti-Flag was pre-conjugated to magnetic beads to capture the antigen. Finally, the RNA bound to the magnetic beads was eluted and purified, and the data were analyzed by qRT-PCR for the detection of circELP2.

### Western blot

Western blot experiment was employed to detect the expression of protein. MRC-5 cells or lung tissues were lysed with radioimmunoprecipitation assay (RIPA, Sparkjade, China, EA0004) buffer and phenylmethylsulfonyl fluoride (PMSF, Sparkjade, China, EA0005) (RIPA: PMSF=100:1). Protein concentration was determined using a BCA protein assay kit (Coolaber, China). Samples of total protein lysate containing 20 μg of protein were subjected to sodium dodecyl sulphate polyacrylamide gel electrophoresis, transferred to polyvinylidene difluoride (PVDF) membranes. The membranes were sealed with 5% skim milk for 2 h, and incubated overnight at 4 °C with anti-collagen III, anti-collagen I, anti-α-SMA, anti-vimentin, anti-GAPDH, anti-FAP, anti-S100A4, anti-p-YAP1, anti- YAP1, anti-MYH9, anti-Myo1c, anti-F-actin, anti-TRIM25, anti-14-3-3ζ, and anti-BRD4. The membranes were washed with 1×Tris Buffered Saline Tween three times and incubated with secondary antibodies at room temperature for 1 h. The expression of the proteins was detected by an enhanced chemiluminescence reagent kit (Spark Jade, China). The dilution ratio and item number of the antibodies were both listed in Table S1 of the supplementary materials.

### Phalloidin staining for cytoskeleton observation

MRC-5 cells or human lung tissue slices were fixed with 4% formaldehyde for half an hour, rinsed twice in PBS, and permeabilized with 0.1% Triton-X 100 in PBS for half an hour. FITC phalloidin (ABclonal, China, RM02836) was added to the cells and incubated for half an hour at room temperature. The MRC-5 cells were stained with DAPI (Solarbio, China, C0060) and blocked with antifade mounting medium (Solarbio, China, S2100) on the cell slides. Images were observed under a laser confocal microscope (Zeiss LSM880, Germany).

### Co-immunoprecipitation (Co-IP)

Co-IP assays were performed using the Co-IP Kit (Absin, China). Lysed 4 × 10^6^ MRC-5 cells were centrifuged at 12,000 × g for 5 min, and the antibody (anti-TRIM25/anti-14-3-3ζ/anti-H3K27ac) was added to the collected supernatant for overnight incubation at 4 °C. Protein A/G agarose beads were added to the supernatant and incubated for 3 h at 4 °C. The beads were collected and centrifuged at 12,000 × g for 1 min at 4 °C, then washed with wash buffer. Proteins were resuspended in the SDS-PAGE sample buffer and heated at 95 °C for 5 min. The samples were analyzed by Western blot for the detection of the binding protein.

### Fluorescence recovery after photobleaching experiment (FRAP)

1 × 10^6^/mL MRC-5 cells were inoculated in a glass dish with a diameter of 3.5 cm. EGFP-TRIM25 and EGFP-TRIM25-∆370-401 fluorescent plasmids (Keyybio, China) were transfected into the cells for 48 h. Droplets formed by *in vitro* TRIM25 protein phase separation were selected, and laser bleached using 488 nm excitation wavelength at 100% power in the centre region of the droplets, the fluorescence recovery was recorded continuously under the microscope after bleaching for 200 s. FRAP experiments were carried out under a laser confocal microscope (Zeiss LSM880), and images were acquired and analyzed using the ZEN software, with FRAP curves plotted.

### Chromatin Immunoprecipitation-PCR (ChIP-PCR)

The Chromatin IP Kit (Cell Signaling Technology, #9005) was used to detect the binding of H3K27ac or YAP1 to MYH9 in MRC-5 cells. 2 × 10^7^ cells were fixed in 1% formaldehyde for 10 min to induce the cross-linking of protein-DNA complexes. After washing with PBS, the cells were treated with PBS containing Protease Inhibitor Cocktail (PIC). MRC-5 cells were centrifuged at 4 °C for 5 min at 2000 × g, and the supernatant was removed. Micrococcal Nuclease and EDTA were used for chromatin digestion. After centrifuged at 4 °C for 1 min at 16,000 × g, cells were resuspended in 1×ChIP buffer containing PIC. Ultrasound was used to break the nuclear membrane, and chromatin preparation was obtained after centrifugation at 9,400 × g for 10 min. Chromatin is diluted by 1×ChIP buffer containing PIC. Then, 10 μL of the supernatant was collected as the 2% Input. Add the immunoprecipitation antibody of H3K27ac (Cell Signaling Technology, USA, 8173S) or YAP1 (Proteintech, China, 13584-1-AP), Normal Rabbit IgG (Cell Signaling Technology, USA, #2729) functions as a negative control to the remaining supernatant, and the samples were rotated and incubated at 4 °C overnight. Protein G Magnetic Beads were added to the samples and incubated by rotation at 4 °C for 2 h and were washed orderly with low-salt and high-salt solutions. The chromatin was eluted from the anti-H3K27ac or YAP1/Protein G Magnetic Beads and cross-linked, and then the DNA was purified using a centrifugation column. Next, the immunoprecipitated DNA was purified and detected through qPCR according to 2×SYBR Green Pro Taq HS Premix (Accurate Biology, AG11701). Primer sequences for qPCR were listed as follows. MYH9-F 5′-CAGCCACTTCTCGGTTCACT -3′ and MYH9-R 5′-CACTGGCCCAGACAGTGTAG-3′.

### Quantitative real-time PCR (qRT-PCR)

qRT-PCR experiments were used to detect the expression levels of circELP2, MYH9, and Myo1c. Total RNA was extracted using TRIzol reagent. Reverse transcription was performed using Evo M-MLV RT Premix reagent (Accurate Biology, AG11706). The qRT-PCR was performed using a 2×SYBR Green Pro Taq HS Premix (Accurate Biology, A3A2291). The reaction conditions of circELP2 were as follows: Predenaturation at 95 °C for 10 min and PCR amplification for 45 cycles at 95 °C for 5 s, 60 °C for 30 s, and 72 °C for 30 s. The reaction conditions of MYH9 and Myo1c were as follows: Pre-denaturation at 50 °C for 2 min, 95 °C for 10 min. PCR amplification for 40 cycles at 95 °C for 10 s, 60 °C for 30 s. circELP2- F 5′- CTGATGAAGAGGAGCTGTTA-3′ and circELP2-R 5′-AGAGAAACACTTTCTGAAAAG-3′; MYH9-F 5′-AAGCTGGTATGGGTGCCTTC-3′ and MYH9-R 5′-CTTGGGCGGGTTCATCTTCT-3′; Myo1c-F 5′-GCACCAGAATCATGGGGAGC-3′ and Myo1c-R 5′-TCCAGTCACTCTTGTCGTTGA- 3′.

### Forced vital capacity (FVC) testing

FVC testing was used to detect the lung function of mice. After underwent intraperitoneal anesthesia, mice were tracheotomized and intubated. Mechanical ventilation was performed with a breathing rate of 150 breaths/min, 10 mL/kg tidal volume, and 3 cm H_2_O PEEP. The negative pressure-driven forced expiratory (NPFE) maneuver was applied. Mice lung was inflated to a pressure of 30 cm H_2_O for 2 s, then the airway was connected to the negative pressure reservoir (-50 cm H_2_O) for 2 s. FVC was calculated directly from the flow cycle generated during lung deflation.

### Immunofluorescence

Immunofluorescence experiment was employed to detect the level of protein and co-localization between proteins. MRC-5 cells were inoculated into 24-well plates and fixed with 4% paraformaldehyde fixative. The paraffin sections of human lung tissues were baked for dewaxing. The human tissue and MRC-5 cell samples were punched with 0.3% TritonX-100 and then washed 3 times with PBS, and then closed with goat serum for 1 h. Primary antibodies were added, and the plates and slices were incubated at 4 °C overnight with antibodies: anti-YAP1 (Proteintech, China, 13584-1-AP), anti-p-YAP1 (Proteintech, China, 29018-1-AP), anti-Acetyl-Histone H3 (Lys27) (H3K27ac) (Cell Signaling Technology, USA, 8173S), anti-F-actin (Abcam, UK, ab1840320), anti-TRIM25 (Proteintech, China, 12573-1-AP), anti-14-3-3ζ (Affinity, China, AF6356). On the next day, fluorescently labeled secondary antibody was added and incubated for 60 min at room temperature. Cell nuclei were stained by DAPI (Solarbio, China, C0060) and then washed with 1×PBS. Finally, antifade mounting medium (Solarbio, China, S2100) was applied to the cell slides. All images were collected under fluorescence microscopy (Invitrogen EVOS M7000, Thermo Fisher, USA).

### Protein stability assay

1 × 10^6^/mL MRC-5 cells were inoculated in cell culture dishes. After corresponding processing according to the groups, 10 μg/mL cycloheximide in serum-free medium was added to the cell samples. Cellular proteins from the same group were extracted after 0, 2, 4, 6, and 8 h of cycloheximide treatment. TRIM25, 14-3-3ξ, and GAPDH protein levels were detected by protein blotting.

### Half-life analysis for circELP2

Half-life analysis was used to detect the RNA stability. 1 × 10^6^/mL MRC-5 cells were inoculated in 6-cm cell culture dishes. After corresponding processing according to the groups, serum-free medium containing 5 μg/mL of actinomycin D was added to the cell samples, and treated for different times (0, 1, 2, 3, 4 h) in each group. Cells samples were collected, and total RNA was extracted for subsequent qRT-PCR assays to detect circELP2.

### 1, 6-hexanediol treatment

1 × 10^6^/mL MRC-5 cells were inoculated in 24-well plates. 5% 1,6-hexanediol (Sigmaaldrich, USA, 240117) was added to the culture medium for 10 min at room temperature after TGF-β1 treatment for 48 h or not, and immunofluorescence staining was carried out. The changes in protein expression were observed under a confocal laser scanning microscope (Zeiss LSM880, Germany).

### H&E and Masson trichrome staining

H&E and Masson trichrome staining were employed to detect the structure and collagen deposition of lung tissue. The isolated tissues were fixed in 4% paraformaldehyde at room temperature for 2 h, and then continued fixation for 48 h at 4 °C. The fixed mice lung tissues were dehydrated and soaked in paraffin overnight. The following day, lung tissues were cut into 4 μm sections using a Leica (RM2255) slicer. The slices were baked at 60 °C for 60 min before deparaffinized and dehydrated with xylene and ethanol, respectively. Hematoxylin and eosin staining solutions were respectively used to stain the nucleus and cytoplasm of cells. Masson trichrome staining was carried out as instructed by Masson's Trichrome Stain Kit (Solarbio, China). The stained sections were dropped with neutral gel, and sealed with coverslips. The lung tissues of each group were observed under light microscopy. H&E staining was semi-quantitatively assessed using the Ashcroft scoring system, which grades the histological features of fibrosis on a scale from 0 to 8. The collagen content of Masson staining was quantified using Image J: [Relative collagen content (%) = blue-stained area in visual field/total area (%)].

### MicroCT observation

The MicroCT imaging system for small animals (PerkinElmer, USA) was warm-up. The mice were subjected to intraperitoneal anesthesia, then positioned flat on the MicroCT machine. The X-ray parameters were adjusted to 90 kV and 88 μA. The resolution of CT images was 36 mm FOV, with an exposure time of 4 min. Two-dimensional tomographic images were obtained by using imaging software (SimpleViewer). Three-dimensional reconstruction was performed by using Analyze 11.0 (AnalyzeDirect, USA). Finally, the MicroCT images were taken and outputted.

### Ubiquitination assay

After transfection for 48 h, MRC-5 cells were treated with 15 μM MG132 (MeilunBio, MB5137) for 6 h. The samples were lysed in the lysis buffer and then treated with Co-IP treatment. Finally, the samples were boiled for 5 min at 95 °C in loading buffer and analyzed by Western blot with the indicated antibodies.

### RNA fluorescence *in situ* hybridization (RNA FISH)

The FISH Kit (Bersin, Bes1001) was used to detect the level of circELP2 in lung tissue slides of humans. Lung sections were orderly subjected to dewaxing, rehydration (100%, 90%, 80%, 70% ethanol), and permeating for later use. After incubating the lung tissue in the pre-hybridization solution at 37 °C for 30 min, the circELP2 probe and the hybridization solution were mixed in a ratio of 1:39, and then dropped onto the lung tissue section for overnight at 37 °C. 25% formamide/2×SSC, 0, 1% NP-40/2×SSC, 0.5×SSC, and 0.2×SSC were orderly used to wash the sections. Added DAPI reagent for staining the cell nucleus. Finally, the sections were sealed with an anti-fluorescence quenching mounting agent and then observed under a confocal microscope.

### Immunofluorescence-fluorescence *in situ* hybridization (IF-FISH)

The IF-FISH Kit (Bersin, Bes1023) was used to detect the co-localization between circELP2 and TRIM25 in MRC-5 cells. MRC-5 cell slides inoculated in 24-well plates were orderly treated with 4% paraformaldehyde for fixation, 0.1% Triton permeating, 1% paraformaldehyde for fixation, and 70%, 80%, 90%, and 100% ethanol for dehydration. The usage and washing of circELP2 probes were the same as the RNA FISH experiment. After the probes were washed, goat serum was added and sealed for 60 min. Then the TRIM25 antibody was added and incubated overnight at 4 °C. The next day, a fluorescently labeled secondary antibody was added for binding to the TRIM25 antibody, then DAPI was added for staining the cell nucleus. Finally, the sections were sealed with an anti-fluorescence quenching mounting agent and then observed under a confocal microscope.

### Acetylation omics analysis

The lung tissue sample was ground and lysed with lysis buffer (8 M urea, 1% protease inhibitor cocktail). The remaining debris was removed by centrifugation at 12,000 × g at 4 °C for 10 min. After that, the BCA kit was employed to detect the protein concentration of the supernatant. For enzymolysis, 20% TCA was added to the sample and mixed up, then precipitated at 4 °C for 2 h. The precipitate was washed 3 times with pre-cooled acetone after centrifuging at 4500 × g for 5 min. Then, 200 mM TEAB was added to the dried precipitate, and ultrasound to disperse the sediment. Added trypsin to the samples at a ratio of 1:50 (protein: enzyme, m/m) and enzymatically hydrolyzed overnight. Added dithiothreitol (DTT) to achieve a final concentration of 5 mM, and then reacted at 56 °C for 30 min. Afterwards, added iodoacetamide (IAA) to reach a final concentration of 11 mM, and incubated at room temperature in the dark for 15 min.

To enrich modified peptides, tryptic peptides dissolved in IP buffer (100 mM NaCl, 1 mM EDTA, 50 mM Tris-HCl, 0.5% NP-40, pH 8.0) were incubated with pre-washed acetylated resin (PTM-104, PTM Bio) at 4 °C overnight with gentle shaking. Then the peptides were washed 4 times with IP buffer and 2 times with deionized water. The bound peptides were eluted 3 times from the resin with 0.1% trifluoroacetic acid (TFA) eluent. Finally, the eluted fractions were combined and vacuum-dried.

For LC-MS/MS analysis, the resulting peptides were dissolved in solvent A (0.1% formic acid, 2% acetonitrile/ in water) and separated using the NanoElute ultra-high performance liquid chromatography system. Then, the peptides were injected into the Capillary electrospray for ionization and then analyzed by the timsTOF Pro mass spectrometer. The electrospray voltage applied was 1.60 kV. The parent ions of the peptide segments and their secondary fragments were all detected and analyzed using high-resolution TOF, with secondary mass spectrometry scan ranges from 100 to 1700 m/z. The timsTOF Pro was operated in parallel accumulation serial fragmentation (PASEF) pattern. After the acquisition of a primary mass spectrum, 10 PASEF mode acquisitions were conducted for secondary spectra with the charge number of the parent ion ranging from 0 to 5. The dynamic exclusion was set to 30 s.

### Assay for transposase-accessible chromatin with high-throughput sequencing (ATAC-seq)

50 μL of pre-cooled RSB (with the addition of 0.1% NP40, 0.1% Tween-20, and 0.01% digitonin) was added to 5 × 10^4^ MRC-5 cells, and then inverted 3 times before being incubated on ice for 3 min. Then, 1 mL of pre-cooled RSB (with only 0.1% Tween-20) was added and mixed 3 times. Centrifuged at 500 × g for 5 min at 4 °C, then discard the supernatant. Added 50 μL of transposition mix, incubated at 37 °C in a shaking incubator at 1000 rpm for 30 min. Purified the DNA using the Qiagen MinElute PCR Purification Kit. Purified the DNA fragments using the Zymo DNA Clean and Concentrator-5 Kit. Then, PCR was employed to DNA amplification. The reaction system is configured as follows: Transposed DNA (10 μL), TAE (1 μL), 5×TAB (10 μL), Primer 1 (2.5 μL), Primer 2 (2.5 μL), PPM (5 μL). The amplification procedure: 98 °C for 3 min, 98 °C for 15 s, 63 °C for 30 s (5-9 cycles), 72 °C for 30 s, 72 °C for 5 min. After the library was qualified, sequencing was performed using the Illumina HiSeq platform. After ATAC-seq sequencing, the raw data were obtained. Subsequently, Unique mapped reads were used for further information analysis.

### Youngs modulus measurement by atomic force microscope (AFM)

2 × 10^5^ MRC-5 cells were cultured in WillCo-dish glass-bottom dishes (Willco Wells BV, GWST-5040). Lung tissue samples were prepared into 8-μm frozen sections. Cell and tissue images were captured in QI mode under an AFM (JPK NanoWizard 4, Bruker Nano GmbH). A PFQNM-LCCAL probe (Bruker Nano GmbH) with an end radius of 75 nm and a force constant of 0.09 N/m was used to acquire topographical images. After an entire cell was imaged by AFM in PBS, the colloid probe (MLCT-O-A probe, Bruker Nano GmbH) with a 20-μm diameter silica sphere was localized on an intact single cell. Youngs modulus was measured using the AFM indentation test based on a Hertz model.

### Statistical analysis

The data represented one of at least three independent experiments and were analyzed using GraphPad Prism 9.5.1. One-way ANOVA followed by Tukey post hoc test was used to determine significance among multiple groups. T-test was used to determine the significance across two samples. All statistical analysis was expressed as mean ± standard deviation (SD), and *p < 0.05 was considered statistically significant.

## Results

### The lung samples of IPF patients exhibit cytoskeleton remodeling with high tension

**Figure 1:**
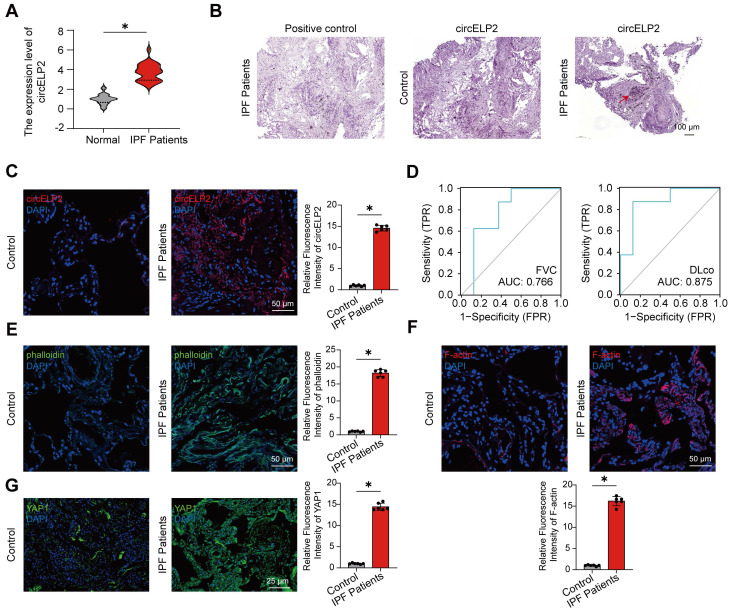
** The lung samples of IPF patients exhibit cytoskeleton remodeling with a high tension. (A)** qRT-PCR displayed that circELP2 expression increased in peripheral blood samples from 25 IPF patients compared with the normal people (n=25). **(B)** RNA scope detection illustrated that circELP2 was increased and primarily localized in the cytoplasm of lung samples from IPF patients. The red arrow represents the positive indicator of circELP2.** (C)** RNA FISH experiment was used to detect the expression of circELP2 in control and IPF lung tissues. The result showed that the level of circELP2 was increased in IPF patients compared with the control group (n=6). **(D)** The ROC analysis of circELP2 and FVC showed sensitivity and specificity values of 87.5% and 62.5%, respectively, in fibrotic patients, with an area under the ROC curve of 0.766. The ROC analysis between circELP2 and DLco indicated sensitivity and specificity values of 87.5% for both metrics in patients, with an area under the ROC curve of 0.875. **(E)** FITC-Phalloidin staining in lung tissue sections showed increased actin filament intensity and aggregation in IPF patients (n=6). **(F)** The immunofluorescence of F-actin in lung tissue sections showed that F-actin accumulation was increased in IPF patients compared with the control group (n=6). **(G)** The immunofluorescence of YAP1 in lung tissue sections showed that YAP1 levels were elevated in IPF patients compared with the control group (n=6).* p* *< 0.05, mean ± SD.

First, the expression of circELP2 was assessed in IPF patients. The qRT-PCR results showed that the circELP2 expression was upregulated in the peripheral blood samples of 25 IPF patients compared with the control people (Fig. 1A). The RNA scope detection illustrated that circELP2 was increased and primarily localized in the cytoplasm of lung samples from IPF patients compared with the control group (Fig. 1B). The RNA FISH experiment proved that the expression of circELP2 in IPF patients was increased compared with the control group (Fig. 1C). The receiver operating characteristic (ROC) curve between circELP2 and FVC showed that the sensitivity and specificity values were 87.5% and 62.5% in the IPF patients, respectively. The area under the ROC curve was 0.766. The sensitivity and specificity values were 87.5% and 87.5% between circELP2 and DLco, respectively. The area under the ROC curve was 0.875 (Fig. 1D). The data indicated the clinical application value of circELP2 as a biomarker for IPF. Interestingly, FITC-Phalloidin staining in lung tissue sections showed increased actin filament intensity and aggregation in IPF patients compared with the control group (Fig. 1E). F-actin, the main component of cytoskeletal microfilaments, is a tension-sensitive anchor and transporter that can sense cellular mechanical forces. The laser scanning confocal microscope observation showed the F-actin accumulation in IPF patients compared with the control group (Fig. 1F). Meanwhile, the expression of YAP1 was increased dramatically in IPF patients compared with the control group (Fig. 1G). YAP1 is known to translate mechanical forces into biochemical signals. Therefore, how YAP1 converts circELP2-mediated biological signals into mechanical forces arouses our interest.

### Highly expressed circELP2 binds with TRIM25 to promote pulmonary fibrosis

**Figure 2:**
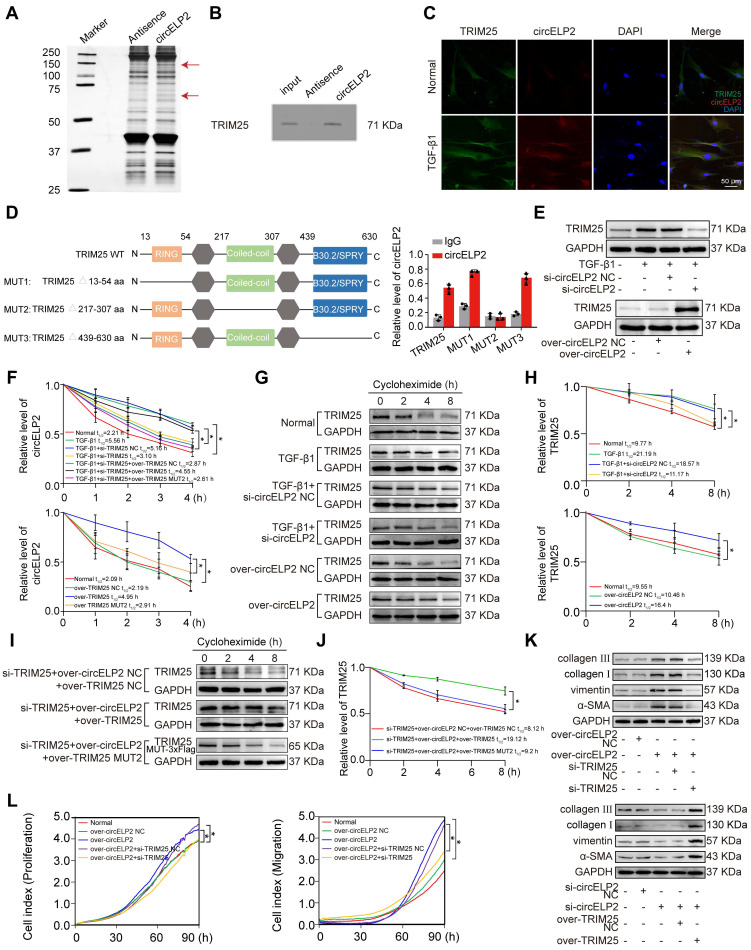
** circELP2 binds with TRIM25 to promote pulmonary fibrosis. (A)** A specific biotin-labeled circELP2 probe was performed an RNA pulldown assay in MRC-5 cells. Silver staining images revealed that the differential protein bands (indicated by red arrows). Antisense RNA was used as a negative control. **(B)** RNA pulldown analysis confirmed that circELP2 bound to the TRIM25 protein. **(C)** MRC-5 cells were treated with or without TGF-β1, followed by an IF-FISH experiment with the circELP2 probe and the TRIM25 antibody. The IF-FISH experiment showed that TRIM25 (green) and circELP2 (red) were co-localized in the cytoplasm. **(D)** The ring domain (MUT1), coiled-coil domain (MUT2), and SPRP domain (MUT3) in TRIM25 were deleted to identify the binding domain in MRC-5 cells. RIP results demonstrated that circELP2 expression was lost after mutation of the 217-307 aa fragment in the coiled-coil domain (n=3). **(E)** MRC-5 cells were transfected with si-circELP2 or over-circELP2 in the presence or absence of TGF-β1. The following Western blot assay showed that the level of TRIM25 was reduced in the TGF-β1+si-circELP2 group compared with the TGF-β1+si-circELP2 negative control (NC) group, and increased in the over-circELP2 group compared with the over-circELP2 negative control (NC) group. **(F)** MRC-5 cells were transfected with si-TRIM25, over-TRIM25, and over-TRIM25 MUT2 plasmids in the presence or absence of TGF-β1, followed by RNA stability assay. The result showed that the stability of circELP2 was enhanced by TRIM25 overexpression but decreased by TRIM25 knockdown; the treatment of over-TRIM25 MUT2 reduced the stability of circELP2 compared with the treatment of over-TRIM25 (n=3). **(G)** MRC-5 cells were transfected with si-circELP2 or over-circELP2 in the presence or absence of TGF-β1, followed by protein stability assay. The result showed that circELP2 overexpression increased the stability of TRIM25, whereas circELP2 knockdown reduced it.** (H)** Quantitative analysis of the protein stability assay of TRIM25 (n=3). **(I)** MRC-5 cells were treated with si-TRIM25, over-circELP2, over-TRIM25, and over-TRIM25 MUT2 plasmids, followed by protein stability assay. The result showed that the treatment of over circELP2+over-TRIM25 MUT2 reduced the stability of TRIM25 compared with the treatment of over circELP2+over-TRIM25 in TRIM25-knockdown cells.** (J)** Quantitative analysis of the protein stability assay of TRIM25 (n=3). **(K)** In MRC-5 cells, rescue experiments were conducted by transfecting over-circELP2, followed by transfection of si-TRIM25, or transfecting si-circELP2, followed by transfection of over-TRIM25. The following Western Blot experiment showed that si-TRIM25 reversed the promoting effect of over-circELP2 on collagen III, collagen I, vimentin, and α-SMA; over-TRIM25 reversed the inhibitory effect of si-circELP2 on collagen III, collagen I, vimentin, and α-SMA. **(L)** Rescue experiment in MRC-5 cells was conducted by transfecting over-circELP2, followed by transfection of si-TRIM25. The following cell proliferation and migration experiments confirmed that si-TRIM25 repressed MRC-5 cell proliferation and migration, and reversed the increasing trend caused by over-circELP2 (n=3). *p < 0.05, mean ± SD.

Next, whether circELP2 can target YAP1 to promote cytoskeleton remodeling leading to the high mechanical forces through its binding protein was explored. TGF-β1-treated MRC-5 cell was used as the cell model of pulmonary fibrosis, in which TGF-β1 activated the fibroblast-to-myofibroblast differentiation. RNA pull-down discovered circELP2 specifically bound to 50-75 KDa proteins (Fig. 2A). Further mass spectrometry analysis revealed that tripartite motif containing 25 (TRIM25) was a binding protein with circELP2. The results were verified via Western blot after RNA pull-down (Fig. 2B). IF-FISH analysis showed that TRIM25 and circELP2 were colocalized in the cytoplasm, and TGF-β1 treatment increased the colocalization (Fig. 2C). To identify the binding domain of TRIM25 with circELP2, the ring domain (13-54 aa), coiled-coil domain (217-307 aa), and SPRP domain (439-630 aa) of TRIM25 were selectively knocked out, with the corresponding mutants named TRIM25 MUT1, TRIM25 MUT2, and TRIM25 MUT3, respectively. RNA immunoprecipitation (RIP) experiments demonstrated that the binding of TRIM25 and circELP2 was disappeared with the treatment of TRIM25 MUT2. The result proved that the 217-307 amino acid (aa) fragment in coiled-coil domain was the binding domain between TRIM25 and circELP2 (Fig. 2D).

Then, to demonstrate the effect of circELP2 on TRIM25 in pulmonary fibrosis, the gain- and loss-of-function experiments elucidated that circELP2 knockdown (si-circELP2) reduced TRIM25 expression in TGF-β1-treated MRC-5 cells, whereas circELP2 overexpression (over-circELP2) increased TRIM25 levels (Fig. 2E). The analysis of stability showed that the half-life of circELP2 was enhanced by the TRIM25 overexpression, but it was reduced by the TRIM25 knockdown. The treatment of over-TRIM25 MUT2 reduced the half-life of circELP2 compared with the treatment of over-TRIM25 (Fig. 2F). The protein stability of TRIM25 was enhanced by the circELP2 overexpression, but it was reduced by the circELP2 knockdown (Fig. 2G, H). To more clearly demonstrate that circELP2 directly enhances TRIM25 protein stability through their specific interaction, we knocked out the endogenous TRIM25 and overexpressed circELP2, then respectively overexpressed TRIM25 and TRIM25 MUT2 to detect the stability of TRIM25. The protein stability experiment showed that the treatment of over circELP2+over-TRIM25 MUT2 reduced the stability of TRIM25 compared with the treatment of over circELP2+over-TRIM25 in TRIM25-knockdown cells (Fig. 2I, J). circELP2 overexpression promoted the expression of collagen I and III, vimentin, α-SMA, and cellular proliferation and migration, but knockdown of TRIM25 reversed the increasing trend caused by circELP2 overexpression. Knockdown of circELP2 inhibited the expression of collagen I and III, vimentin, and α-SMA, but TRIM25 overexpression reversed their decreasing trend caused by si-circELP2 (Fig. 2K, L). Collectively, these findings suggest that circELP2 promotes pulmonary fibrosis dependent on its binding protein TRIM25 *in vitro*.

**Figure 3:**
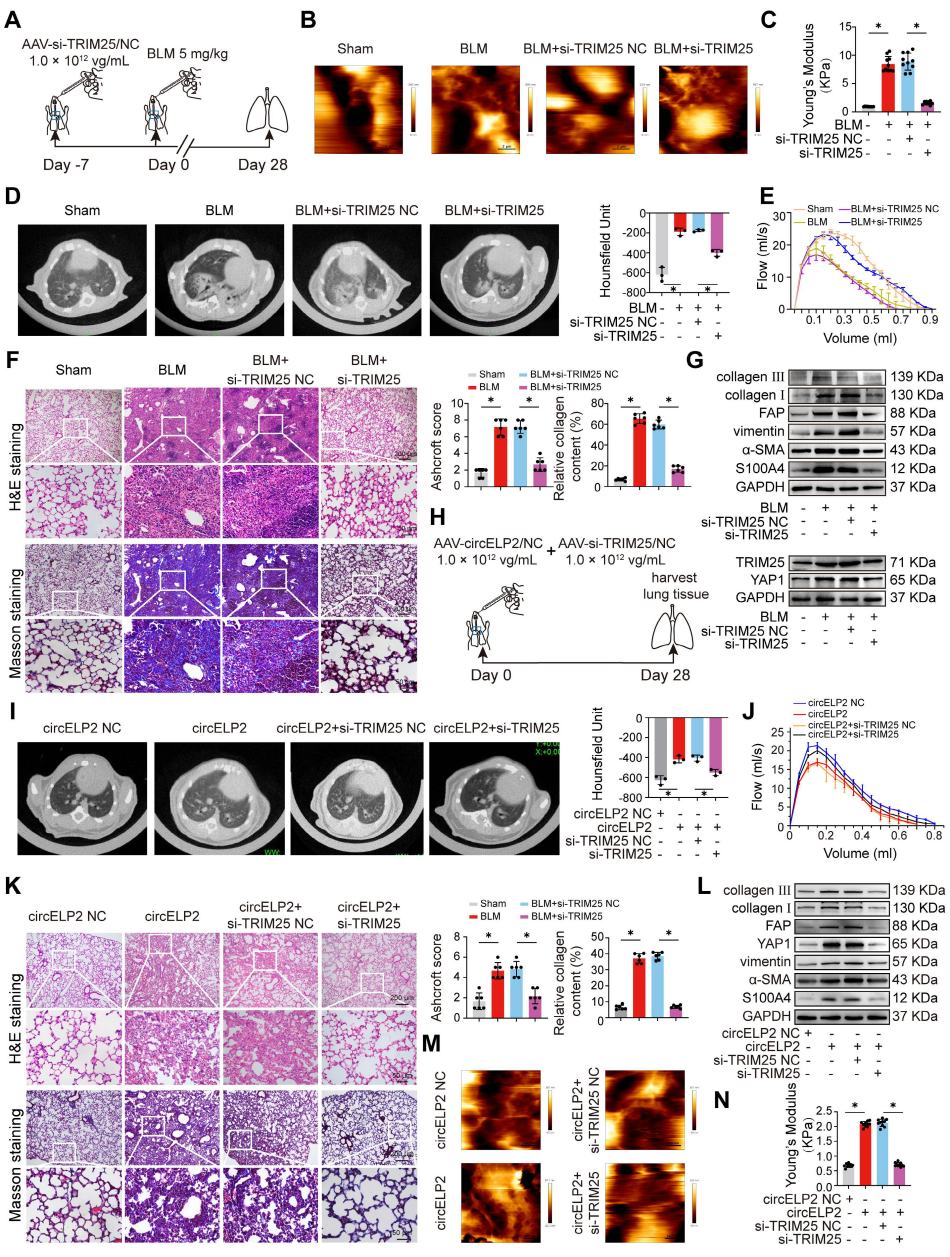
** circELP2 enhances cell mechanical forces via TRIM25 to deteriorate pulmonary fibrosis in mice. (A)** Schematic illustration of spraying AAV-si-TRIM25/NC into mice. **(B)** The representative image of AFM in mice treated with BLM, BLM+si-TRIM25 negative control (NC), and BLM+si-TRIM25. **(C)** The detection of Youngs modulus showed that si-TRIM25 treatment reduced the value of Youngs modulus (n=10). **(D)** Micro CT result showed a diffuse degree of fibrosis in the BLM and BLM+si-TRIM25 negative control (NC) groups. The extent of fibrosis in the BLM+si-TRIM25 group was significantly lower compared with the BLM+si-TRIM25 negative control (NC) group (n=3). **(E)** Lung function assessments revealed that TRIM25 knockdown alleviated the decline in lung function induced by BLM. **(F)** H&E revealed thinner alveolar walls in the BLM+si-TRIM25 group compared with the BLM+si-TRIM25 negative control (NC) group. Masson's staining showed that the collagen deposition (blue) decreased in the BLM+si-TRIM25 group compared with the BLM+si-TRIM25 negative control (NC) group (n=6). **(G)** Western blot analysis confirmed decreased levels of collagen III, collagen I, FAP, vimentin, α-SMA, S100A4, TRIM25, and YAP1 in the BLM+si-TRIM25 group compared with the BLM+si-TRIM25 negative control (NC) group. **(H)** Schematic illustration of spraying AAV-circELP2 and AAV-si-TRIM25/NC into mice. **(I)** Micro CT result showed a fibrotic lesion in the circELP2 group. si-TRIM25 significantly mitigated the pro-fibrotic effects of circELP2 (n=3).** (J)** Lung function assessments revealed that TRIM25 knockdown alleviated the decline in lung function induced by circELP2 overexpression. **(K)** H&E staining indicated thicker alveolar walls and collagen fibers in the circELP2 group, whereas si-TRIM25 significantly reversed the fibrotic characteristics induced by circELP2. Masson staining revealed that the collagen deposition (blue) was more significant in the circELP2 group, with a marked decrease after TRIM25 knockdown (n=6). **(L)** Western blot results illustrated high expression levels of collagen III, collagen I, FAP, vimentin, α-SMA, S100A4, and YAP1 after circELP2 overexpression, whereas the levels of proteins were notably decreased by TRIM25 interference. **(M)** The representative image of AFM in mice treated with circELP2 negative control (NC), circELP2, circELP2+si-TRIM25 negative control (NC), and circELP2+si-TRIM25. **(N)** The value of Youngs modulus in the circELP2+si-TRIM25 group was significantly lower than the circELP2+si-TRIM25 negative control (NC) group (n=10). *p < 0.05, mean ± SD.

Next, that circELP2 exerts profibrotic function via TRIM25 was confirmed *in vivo*. First, the small interference fragment of TRIM25 (si-TRIM25) was packaged into AAV (AAV-si-TRIM25/NC) and sprayed into mice before 7 days of BLM spraying. The lung tissue was harvested after 28 days of BLM treatment (Fig. 3A). Frozen sections of lung tissue were observed by atomic force microscopy. The morphological images of mechanical force showed a rough lung surface in the BLM group compared with the sham group, and the lung surface was smooth and flat in the BLM+si-TRIM25 group compared with the BLM+si-TRIM25 negative control (NC) group (Fig. 3B). Material elasticity is commonly represented by the Youngs modulus, which is the linear resistance of materials to elastic deformation under stress. The detection of Youngs modulus showed that si-TRIM25 reduced the value of Youngs modulus, suggesting that the tissue stiffness was significantly reduced after si-TRIM25 treatment in pulmonary fibrosis (Fig. 3C). The MicroCT images, lung function, H&E and Masson staining confirmed the ability of the si-TRIM25 treatment to improve the diffuse degree of fibrosis, lung function, lung structure, and collagen deposition (Fig. 3D-F). The expression of fibrotic proteins and the targeting proteins TRIM25 and YAP1 diminished in the si-TRIM25-treated mice (Fig. 3G). The data indicated that TRIM25 is a profibrotic factor, and interference with TRIM25 can attenuate pulmonary fibrosis.

Then, the rescue experiments were further performed to verify that the profibrotic function of circELP2 depended on TRIM25 in mice. AAV-circELP2 and AAV-si-TRIM25/NC were packaged and sprayed into mice (Fig. 3H). The Micro CT, lung function, H&E and Masson staining, Western blot confirmed that the lung morphology, lung function, lung structure, and collagen deposition had an abnormal performance after circELP2 overexpression compared with the circELP2 negative control (NC) group, whereas si-TRIM25 treatment improved the condition of the lungs (Fig. 3I-L). Frozen sections of lung tissue were observed by atomic force microscopy. The morphological images of mechanical force showed that the circELP2+si-TRIM25 group had a smoother surface topology than the circELP2 and the circELP2+si-TRIM25 negative control (NC) groups (Fig. 3M). The value of Youngs modulus showed that the circELP2+si-TRIM25 group had significantly lower Youngs modulus than the circELP2+si-TRIM25 negative control (NC) groups, suggesting that the tissue stiffness was significantly reduced by si-TRIM25 (Fig. 3N). All the above results indicated that circELP2 acts as a profibrotic factor dependent on TRIM25 *in vivo* and *in vitro*, in which mechanical forces increased significantly.

### TRIM25 protein forms liquid-liquid phase separation (LLPS) structure in pulmonary fibrosis

**Figure 4:**
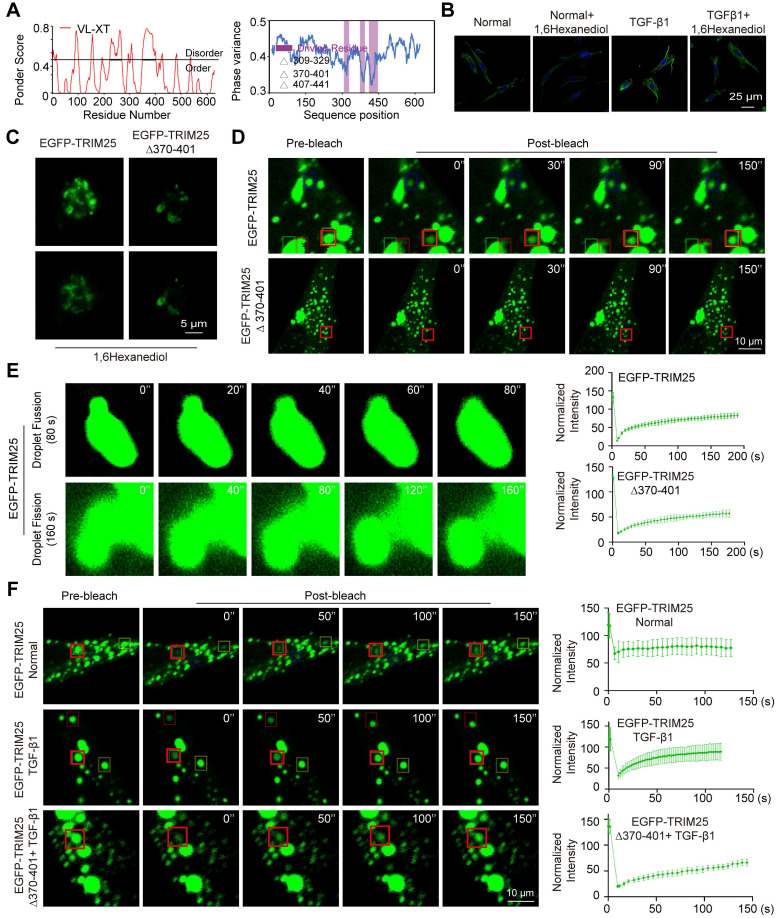
** TRIM25 phase separation is enhanced in the MRC-5 cell model of pulmonary fibrosis. (A)** PSP Hunter predicted that the three key phase-forming regions of the TRIM25 protein were located within the amino acid fragments 309-329, 370-401, and 407-441. The Ponder database indicated that TRIM25 contained two IDRs in the 82-188 and 358-435 fragments. **(B)** MRC-5 cells were treated with 1,6-hexanediol with or without TGF-β1, followed by the immunofluorescence experiment. The result showed that the fluorescence intensity of TRIM25 (green) was significantly increased in TGF-β1-treated MRC-5 cells, and decreased with 1,6-hexanediol treatment in both the normal and TGF-β1-treated cells. **(C)** MRC-5 cells were transfected with EGFP-TRIM25, and EGFP-TRIM25-∆370-401, followed by the immunofluorescence experiment. The result showed that MRC-5 cells in the EGFP-TRIM25-∆370-401 group exhibited significantly fewer punctate structures than those in the EGFP-TRIM25 group. The addition of 1,6-hexanediol markedly reduced the number of punctate structures. **(D)** MRC-5 cells were transfected with EGFP-TRIM25, and EGFP-TRIM25-∆370-401, followed by FRAP analysis. The result showed that the fluorescence recovery was significantly reduced in the EGFP-TRIM25-∆370-401 group compared with the EGFP-TRIM25 group. **(E)** Fusion and fission of phase-separated droplets of EGFP-TRIM25 in MRC-5 cells were visualized using laser confocal microscopy. Two adjacent TRIM25 droplets rapidly fused into a single droplet within 80 s, and these fused droplets underwent fission within 160 s in MRC-5 cells expressing EGFP-TRIM25. **(F)** MRC-5 cells were transfected with EGFP-TRIM25, TRIM25-∆370-401 with or without TGF-β1, followed by FRAP analysis. The result showed that the fluorescence recovery after fluorescence bleaching was significantly enhanced in the TGF-β1-treated group compared with the normal group, and notably diminished in the EGFP-TRIM25 ∆370-401+ TGF-β1 group compared with the EGFP-TRIM25+ TGF-β1 group. The signal intensity relative to pre-bleaching values (y-axis) was plotted against time relative to photobleaching (x-axis).

LLPS structure, characterized as a novel ubiquitous non-membranous organelle, consists of proteins and non-coding RNA that govern their interactions and functions 31. The intrinsically disordered region (IDR) serves as a crucial driver of protein phase separation. The PSP Hunter and Ponder databases indicated that the TRIM25 protein possessed a significant number of IDRs, and the overlapping IDR is 370-401 aa fragment (Fig. 4A). 1,6-hexanediol disrupts the weak hydrophobic forces between molecules and is widely utilized as a phase separation inhibitor. The effect of 1,6-hexanediol on TRIM25 protein phase separation was analyzed. Fluorescence images revealed a reduction in fluorescent brightness in MRC-5 cells treated with 1,6-hexanediol (Fig. 4B). Subsequently, plasmids encoding enhanced green fluorescent protein (EGFP) tagged TRIM25 (EGFP-TRIM25) and a truncated version of the 370-401 aa fragment (EGFP-TRIM25-∆370-401) were constructed and transfected into MRC-5 cells. Fluorescence imaging demonstrated that EGFP-TRIM25-∆370-401 formed significantly fewer punctate structures compared with EGFP-TRIM25. Furthermore, the addition of 1,6-hexanediol substantially decreased the number of punctate structures (Fig. 4C). These findings confirmed that TRIM25 is capable of forming phase separation structures, and the 370-401 aa fragment plays a critical role in this process.

To prove whether TRIM25 phase separation occurs through the 370-401 aa fragment, fluorescence recovery after photobleaching (FRAP) was employed to examine the dynamic change of TRIM25 foci. The results revealed that both EGFP-TRIM25 and EGFP-TRIM25-∆370-401 were expressed in MRC-5 cells. The fluorescence recovery was significantly reduced in the EGFP-TRIM25-∆370-401 group compared with the EGFP-TRIM25 group (Fig. 4D). Condensates formed via phase separation typically exhibit liquid-like properties, such as the ability of neighboring droplets to spontaneously fuse and rapid recovery after bleaching. The fusion and fission of the phase-separated droplets were monitored using a laser confocal microscope over an extended period. It was observed that two adjacent TRIM25 droplets swiftly fused into a larger single droplet within 80 s, and the two fused droplets completed splitting within 160 s (Fig. 4E). Subsequently, the effect of TGF-β1 treatment on TRIM25 phase separation was examined in MRC-5 cells. FRAP images indicated that the fluorescence recovery after fluorescence bleaching was significantly enhanced in the TGF-β1-treated group compared with the normal group. The fluorescence recovery was notably diminished in the EGFP-TRIM25 ∆370-401+ TGF-β1 group compared with the EGFP-TRIM25+ TGF-β1 group (Fig. 4F). These findings suggest that the 370-401 aa fragment facilitates TRIM25 phase separation, and the TRIM25 phase separation structure participates in the progression of pulmonary fibrosis. Consequently, further research focused on how circELP2 affects TRIM25 protein phase separation to promote pulmonary fibrosis.

### circELP2 promotes TRIM25 phase separation via TRIM25 deacetylation

**Figure 5:**
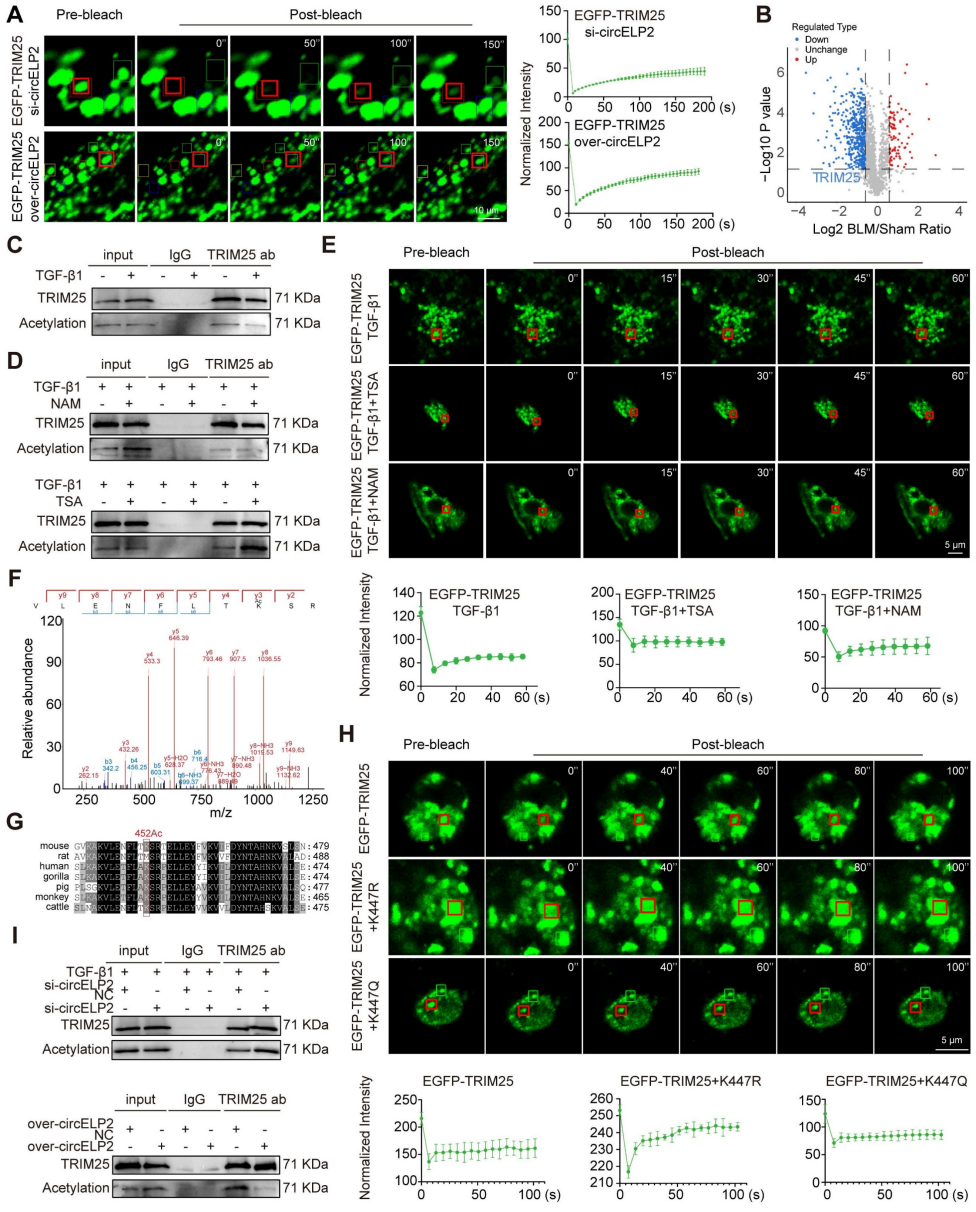
** circELP2 promotes TRIM25 phase separation via TRIM25 deacetylation. (A)** MRC-5 cells were transfected with si-circELP2, and over-circELP2, followed by FRAP analysis. The result showed that the mobility of TRIM25 fluorescent droplets was significantly enhanced after circELP2 overexpression, and significantly reduced after circELP2 knockdown. **(B)** The volcano plot analysis of acetylation omics. **(C)** MRC-5 cells were either treated with or without TGF-β1, followed by Co-IP analysis. The result showed that the acetylation modification decreased in the TGF-β1-treated group compared with the normal group. Input represents the positive control; IgG serves as the negative control. **(D)** MRC-5 cells were treated with Trichostatin A (TSA) and Nicotinamide (NAM) in the presence of TGF-β1, followed by Co-IP analysis. The result showed that TSA treatment increased the levels of acetylation modification compared with the TGF-β1 group, while NAM treatment had no significant effect on the acetylation modification compared with the TGF-β1 group. **(E)** The FRAP results showed that TSA treatment reduced the mobility of TRIM25 fluorescent droplets compared with the TGF-β1 group, while NAM treatment had no significant effect. **(F)** The analysis of the acetylation site of TRIM25 in mice. **(G)** Conservation analysis of the acetylation site between mice and humans showed that the K452 site in mice corresponded to the K447 site in humans. **(H)** MRC-5 cells were transfected with EGFP-TRIM25, EGFP-TRIM25-K447R, and EGFP-TRIM25-K447Q, followed by FRAP assay. The result showed that in contrast to the EGFP-TRIM25 group, the fluorescence recovery was significantly enhanced in the EGFP-TRIM25-K447R group. The fluorescence recovery showed no significant difference in the EGFP-TRIM25-K447Q group. K represents lysine, R represents arginine, and Q represents glutamine. **(I)** MRC-5 cells were transfected with si-circELP2, over-circELP2 with or without TGF-β1, followed by Co-IP analysis. The result showed that the acetylation modification was increased after si-circELP2 treatment and decreased after over-circELP2 treatment.

The effect of circELP2 on the phase separation of TRIM25 was further investigated through knockdown and overexpression of circELP2 in MRC-5 cells. FRAP analysis revealed a significant enhancement in the mobility of TRIM25 fluorescent droplets after overexpressed circELP2, and significantly reduced after knockdown circELP2 (Fig. 5A). These findings indicated that circELP2 facilitates the formation of TRIM25 phase separation. Therefore, how circELP2 regulates TRIM25 phase separation was further investigated.

Acetylation modification can be highly enriched in the intrinsic disordered region of proteins, playing an important role in the regulation of phase separation 32. Hence, acetylation omics analysis was performed, and the volcano plot analysis showed that the level of acetylation modification of TRIM25 was decreased in BLM-treated mice compared with the sham group (Fig. 5B). Then, Co-IP experiments were conducted to confirm that TRIM25 protein underwent acetylation modification, and indicated that TRIM25 bound to pan-acetylation antibody. The acetylation modification was decreased in the TGF-β1-treated group compared with the normal group (Fig. 5C). The level of acetylation can be inhibited by histone deacetylase (HDAC). Therefore, we added the inhibitors of different HDAC families to MRC-5 cells with the treatment of TGF-β1 to detect the level of acetylation of TRIM25. Trichostatin A (TSA) serves as the HDAC Class I/II inhibitor, and Nicotinamide (NAM) serves as the HDAC Class III (SIRT) inhibitor. The following Co-IP results demonstrated that TSA treatment increased the levels of acetylation modification compared with the TGF-β1 group, while NAM treatment had no significant effect on the acetylation modification compared with the TGF-β1 group in MRC-5 cells (Fig. 5D). The results indicate that the acetylation level of TRIM25 was regulated by HDAC Class I/II. To verify the effect of acetylation on phase separation of TRIM25, the FRAP results showed that TSA treatment reduced the mobility of TRIM25 fluorescent droplets compared with the TGF-β1 group, while NAM treatment had no significant effect (Fig. 5E). The results suggest that HDAC Class I/II-mediated deacetylation is involved in the regulation of phase separation of TRIM25.

Modification omics analysis identified that K452 is the acetylation site of TRIM25 in mice (Fig. 5F). Considering that MRC-5 cells are lung fibroblasts of human, the conservation of acetylation site between mice and humans was analyzed. The result showed that the K452 site in mice corresponded to the K447 site in humans (Fig. 5G). Subsequently, the K447 site was mutated to non-acetylated arginine (TRIM25 K447R) and acetyl-mimetic glutamine (TRIM25 K447Q) to further examine the role of acetylation in TRIM25 phase separation in MRC-5 cells. FRAP analysis showed that the mobility of TRIM25 fluorescent droplets was significantly increased in the EGFP-TRIM25+K447R group compared with the EGFP-TRIM25 group; however, no significant difference in mobility was observed in the EGFP-TRIM25+K447Q group (Fig. 5H), indicating TRIM25 deacetylation at K447 site promotes TRIM25 phase separation.

Finally, the effect of circELP2 on the acetylation modification of TRIM25 protein was detected. Co-IP result illustrated that the binding of TRIM25 protein to pan-acetylated protein was significantly increased under the si-circELP2 action, while the binding amount was reduced under the overexpressed circELP2 action (Fig. 5I). These results suggest that circELP2 promotes TRIM25 phase separation through TRIM25 deacetylation at K447 site.

### circELP2 enhances ubiquitination degradation of 14-3-3ζ by promoting deacetylated TRIM25 phase separation

**Figure 6:**
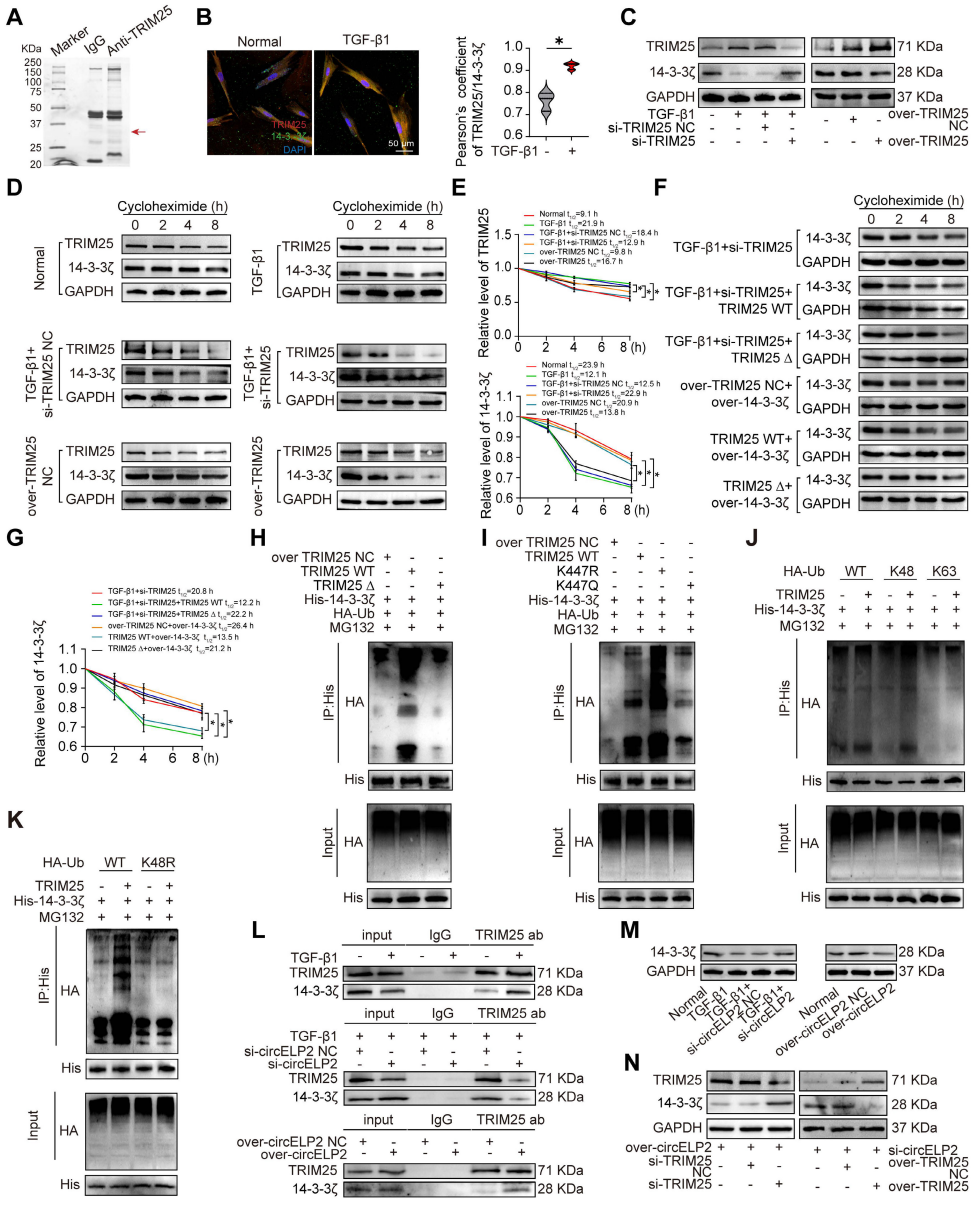
** circELP2 enhances ubiquitination degradation of 14-3-3ζ by promoting phase separation of deacetylated TRIM25. (A)** Coomassie brilliant blue staining demonstrated the presence of several differential protein bands from TRIM25 Co-IP. IgG serves as the antibody control group. **(B)** MRC-5 cells were either treated with or without TGF-β1, followed by immunofluorescence with TRIM25 and 14-3-3ζ antibodies. The result showed that the co-localization of TRIM25 (red) and 14-3-3ζ (green) in the cytoplasm was significantly enhanced in the TGF-β1-treated group compared with the normal group (n=3). **(C)** MRC-5 cells were transfected with si-TRIM25, over-TRIM25 with or without TGF-β1, followed by Western blot assay. The result showed that si-TRIM25 inhibited TRIM25 and promoted 14-3-3ζ expression. over-TRIM25 increased TRIM25 and decreased 14-3-3ζ expression. **(D)** MRC-5 cells were transfected with si-TRIM25, over-TRIM25 with or without TGF-β1, followed by the protein stability assay. The result showed that TGF-β1 and over-TRIM25 increased the stability of TRIM25, decreased the stability of 14-3-3ζ. si-TRIM25 elevated the stability of 14-3-3ζ, whereas reduced the stability of TRIM25. **(E)** Quantitative analysis of the protein stability assay of TRIM25 and 14-3-3ζ (n=3). **(F)** MRC-5 cells were transfected with si-TRIM25, TRIM25 WT, TRIM25 ∆, over-14-3-3ζ with or without TGF-β1, followed by the protein stability assay. The result showed that the stability of 14-3-3ζ was enhanced after the treatment of TRIM25 ∆ compared with the treatment of TRIM25 WT. **(G)** Quantitative analysis of the protein stability assay of 14-3-3ζ (n=3). **(H)** MRC-5 cells were transfected with His-14-3-3ζ, HA-Ub, over-TRIM25 negative control (NC), TRIM25 WT, and TRIM25 ∆, followed by Co-IP assay. The result showed that 14-3-3ζ ubiquitination was decreased after the 370-401 aa fragment was deleted compared with the treatment of TRIM25 WT.** (I)** MRC-5 cells were transfected with His-14-3-3ζ, HA-Ub, over-TRIM25 negative control (NC), TRIM25 WT, TRIM25 K447R, and TRIM25 K447Q, followed by Co-IP assay. The result showed that 14-3-3ζ ubiquitination was increased after the K447 mutation to K447R, and no significant difference after the K447 mutation to K447Q contrast to the TRIM25 WT group. **(J)** MRC-5 cells were transfected with His-14-3-3ζ and HA-Ub (WT, K48 only or K63 only) with or without TRIM25, followed by Co-IP assay. The result showed that K48-linked polyubiquitination was increased after the treatment of TRIM25 compared with K63-linked polyubiquitination.** (K)** MRC-5 cells were transfected with His-14-3-3ζ and HA-Ub (WT or K48 mutant) with or without TRIM25, followed by Co-IP assay. The K48-mutated (K48R) ubiquitin result demonstrated no increase in 14-3-3ζ ubiquitination when TRIM25 was present. **(L)** MRC-5 cells were transfected with si-circELP2, over-circELP2 with or without TGF-β1, followed by Co-IP analysis. The result showed that TRIM25 directly interacted with 14-3-3ζ under the TGF-β1 action. The binding affinity between TRIM25 and 14-3-3ζ decreased after circELP2 knockdown, and increased after circELP2 overexpression. **(M)** MRC-5 cells were transfected with si-circELP2, over-circELP2 with or without TGF-β1, followed by the Western blot assay with 14-3-3ζ antibody. The level of 14-3-3ζ was increased by si-circELP2 and decreased by overexpressed circELP2. **(N)** In MRC-5 cells, rescue experiments were conducted by transfecting over-circELP2, followed by transfection of si-TRIM25, or transfecting si-circELP2, followed by transfection of over-TRIM25. The following Western Blot experiment showed that si-TRIM25 reversed the effects of circELP2 overexpression on both TRIM25 and 14-3-3ζ. TRIM25 overexpression counteracted the effects of circELP2 knockdown on TRIM25 and 14-3-3ζ. *p < 0.05, mean ± SD.

TRIM25 is recognized as an E3 ubiquitin ligase. Co-IP was conducted to investigate its substrate, then found a distinct band in the 25-37 KDa range (Fig. 6A). Further, mass spectrometry discovered that 14-3-3ζ is one of the binding proteins. Immunofluorescence double-labeling experiment confirmed the co-localization of TRIM25 and 14-3-3ζ in the cytoplasm. Notably, the co-localization of TRIM25 and 14-3-3ζ was significantly enhanced in the TGF-β1-treated group compared with the normal group (Fig. 6B). si-TRIM25 reduced TRIM25 levels but increased 14-3-3ζ expression, whereas overexpressed TRIM25 elevated TRIM25 levels but decreasing 14-3-3ζ expression (Fig. 6C). Protein stability experiments revealed that TGF-β1 and over-TRIM25 increased the stability of TRIM25, decreased the stability of 14-3-3ζ. si-TRIM25 elevated the stability of 14-3-3ζ, whereas reduced the stability of TRIM25 (Fig. 6D, E). The rescue protein stability experiment revealed that the overexpression of TRIM25 WT reduced the stability of 14-3-3ζ, thereby reversing the effect of si-TRIM25. Furthermore, the stability of 14-3-3ζ was increased under the treatment of TRIM25-∆370-401 compared with the treatment of TRIM25 WT (Fig. 6F, G). Co-IP results showed that 14-3-3ζ ubiquitination was reduced under the treatment of TRIM25-∆370-401 compared with the treatment of TRIM25 WT (Fig. 6H). These findings suggest that 14-3-3ζ is a substrate of TRIM25, and TRIM25 phase separation promotes the ubiquitination degradation of 14-3-3ζ.

K447R and K447Q were used to further investigate the promotion of 14-3-3ζ ubiquitination degradation depending on the TRIM25 deacetylation. The Co-IP results confirmed a significant increase in 14-3-3ζ ubiquitination after K447 mutation to K447R, and no significant difference after K447 mutation to K447Q in contrast to TRIM25 WT group (Fig. 6I). TRIM25, as an E3 ubiquitin ligase, can prominently catalyze the formation of K48-linked or K63-linked polyubiquitin chains on its substrate proteins, which are respectively related to the ubiquitin-proteasome degradation process and cellular signaling pathways 33, 34. To ascertain the type of ubiquitination of 14-3-3ζ, HA-Ub (wild type, K48 only, K63 only) was transfected into MRC-5 cells with or without TRIM25. The Co-IP result showed that TRIM25 increased the K48-linked polyubiquitination on 14-3-3ζ but had no discernible impact on the K63-linked polyubiquitination (Fig. 6J). Additionally, the K48-mutated (K48R) ubiquitin result demonstrated no increase in 14-3-3ζ ubiquitination when TRIM25 was present (Fig. 6K). Subsequently, Co-IP experiments validated that circELP2 regulation of 14-3-3ζ is dependent on TRIM25. Knockdown of circELP2 inhibited the binding between TRIM25 and 14-3-3ζ, whereas overexpression of circELP2 promoted the interaction (Fig. 6L). 14-3-3ζ levels were increased by si-circELP2 but decreased by overexpressed circELP2 (Fig. 6M). According to the rescue experiments, si-TRIM25 reversed the effects of overexpressed circELP2 on both TRIM25 and 14-3-3ζ, while overexpressed TRIM25 opposed the effects of circELP2 knockdown on TRIM25 and 14-3-3ζ (Fig. 6N). Collectively, all the above data indicated that circELP2 enhances K48-linked ubiquitination degradation of 14-3-3ζ by promoting the phase separation of deacetylated TRIM25.

### 14-3-3ζ-mediated YAP1 nuclear translocation promotes the formation of super-enhancer to activate the expression of cytoskeleton genes

**Figure 7:**
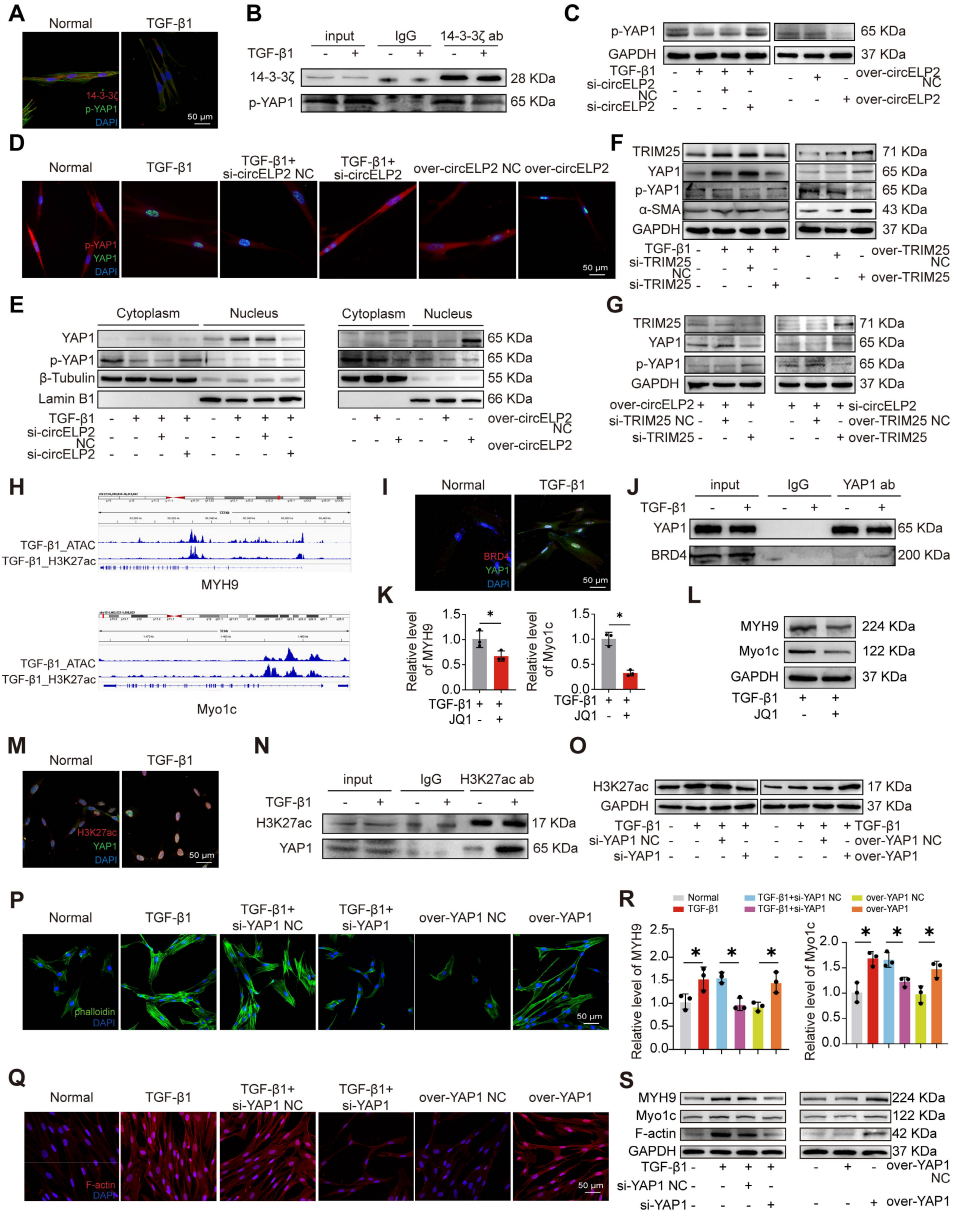
** 14-3-3ξ-mediated YAP1 nuclear translocation promotes the formation of super-enhancer. (A)** MRC-5 cells were either treated with or without TGF-β1, followed by immunofluorescence with 14-3-3ζ and p-YAP1 antibodies. The result showed that 14-3-3ζ (red) and p-YAP1 (green) co-localized in the cytoplasm, and both showed decreased expression in the TGF-β1-treated group. **(B)** MRC-5 cells were either treated with or without TGF-β1, followed by Co-IP assay. The result showed that the binding between 14-3-3ζ and p-YAP1 was reduced in the TGF-β1-treated group. **(C)** MRC-5 cells were transfected with si-circELP2, over-circELP2 with or without TGF-β1, followed by Western blot assay. The result showed that knockdown of circELP2 increased p-YAP1 expression, whereas overexpression of circELP2 decreased it. **(D)** MRC-5 cells were transfected with si-circELP2, over-circELP2 with or without TGF-β1, followed by the immunofluorescence assay with YAP1 and p-YAP1 antibodies. The result showed that knockdown of circELP2 decreased YAP1 (green) expression, whereas increased cytoplasmic p-YAP1 (red) expression. Overexpressed circELP2 had the opposite effect. **(E)** MRC-5 cells were transfected with si-circELP2, over-circELP2 with or without TGF-β1, followed by Nuclear-cytoplasmic separation and Western blot experiment. β-Tubulin serves as the cytoplasmic control, and Lamin B1 serves as the nuclear control. The result showed that circELP2 knockdown elevated cytoplasmic p-YAP1 levels and reduced nuclear YAP1 expression, whereas circELP2 overexpression elevated nuclear YAP1 levels and reduced cytoplasmic p-YAP1 expression. **(F)** MRC-5 cells were transfected with si-TRIM25, over-TRIM25 with or without TGF-β1, followed by the Western blot experiment. The result showed that knockdown TRIM25 decreased TRIM25, YAP1, and α-SMA levels, and increased p-YAP1 level. Overexpression of TRIM25 increased TRIM25, YAP1, and α-SMA levels, and decreased p-YAP1 level. **(G)** In MRC-5 cells, rescue experiments were conducted by transfecting over-circELP2, followed by transfection of si-TRIM25, or transfecting si-circELP2, followed by transfection of over-TRIM25. The following Western Blot experiment showed that knockdown of TRIM25 reversed the effect of overexpressed circELP2 on YAP1 and p-YAP1. Overexpression of TRIM25 reversed the effect of circELP2 knockdown on YAP1 and p-YAP1 levels. **(H)** ATAC-seq and ChIP-seq analyses depicted the enrichment of MYH9 and Myo1c. **(I)** MRC-5 cells were either treated with or without TGF-β1, followed by the immunofluorescence assay. The result showed that YAP1 (green) and BRD4 (red) co-localized within the nucleus, and TGF-β1 treatment led to an increase in nuclear YAP1 and BRD4 expression. **(J)** MRC-5 cells were either treated with or without TGF-β1, followed by the Co-IP experiment. The experiment confirmed the specific binding between YAP1 and BRD4, and TGF-β1 treatment further enhanced the interaction.** (K)** MRC-5 cells were treated with TGF-β1 with or without JQ1. The following qRT-PCR result showed that the gene levels of MYH9 and Myo1c were significantly decreased after JQ1 treatment (n=3).** (L)** Western blot experiments showed that the protein levels of MYH9 and Myo1c were significantly decreased after JQ1 treatment. **(M)** MRC-5 cells were either treated with or without TGF-β1, followed by immunofluorescence with anti-H3K27ac and anti-YAP1 antibodies. The result showed that H3K27ac (red) and YAP1 (green) co-localized within the nucleus. The co-localization was enhanced in the TGF-β1-treated group. **(N)** MRC-5 cells were either treated with or without TGF-β1, followed by the Co-IP experiment. The result showed that the binding of H3K27ac to YAP1 was enhanced in the TGF-β1-treated group. **(O)** MRC-5 cells were transfected with si-YAP1, over-YAP1 in the presence of TGF-β1, followed by the Western blot experiment. The result showed that H3K27ac was increased in the TGF-β1-treated group, reduced after YAP1 knockdown, and elevated after YAP1 overexpression. **(P)** MRC-5 cells were transfected with si-YAP1, over-YAP1 with or without TGF-β1, followed by the FITC-phalloidin experiment. The result showed that the actin filament (green) intensity and aggregation were increased after YAP1 overexpression. YAP1 knockdown reduced actin filament intensity and aggregation. **(Q)** The immunofluorescence showed that F-actin (red) accumulation was increased in the overexpressed YAP1 group, and YAP1 knockdown reduced the accumulation of F-actin. **(R)** The qRT-PCR results showed that si-YAP1 reduced MYH9 and Myo1c levels. YAP1 overexpression increased MYH9 and Myo1c levels (n=3). **(S)** The Western blot experiment showed that F-actin, MYH9, and Myo1c were increased by overexpressing YAP1 and decreased by YAP1 knockdown. *p < 0.05, mean ± SD.

The previous study has demonstrated that circELP2 targets YAP1 via sponging miRNA-630 24, so whether circELP2 regulates YAP1 via targeting TRIM25-14-3-3ζ was further explored. First, the specific binding between 14-3-3ζ and p-YAP1 was tested by immunofluorescence. The results showed that 14-3-3ζ and p-YAP1 were primarily localized in the cytoplasm, and the expression of both 14-3-3ζ and p-YAP1 was decreased in TGF-β1-treated groups (Fig. 7A). The specific binding between 14-3-3ζ and p-YAP1 was further verified by Co-IP, and the binding was decreased in TGF-β1-treated group (Fig. 7B). These findings confirmed the direct interaction between 14-3-3ζ and p-YAP1. Phosphorylation and dephosphorylation play critical roles in regulating YAP1 translocation from nucleus to cytoplasm. Thus, the effects of circELP2 and TRIM25 on YAP1 and p-YAP1 were examined. Western blot analysis revealed that circELP2 knockdown increased p-YAP1 expression, whereas circELP2 overexpression decreased p-YAP1 levels (Fig. 7C). Immunofluorescence analysis and nuclear-cytoplasmic separation experiments further showed that circELP2 knockdown elevated cytoplasmic p-YAP1 levels and reduced nuclear YAP1 expression, whereas circELP2 overexpression produced the opposite effects (Fig. 7D-E). Subsequently, we explored whether the enhancement of circELP2 on YAP1 dephosphorylation was dependent on TRIM25. TRIM25 knockdown reduced TRIM25, YAP1, and α-SMA expression, while increasing p-YAP1 levels. Conversely, TRIM25 overexpression elevated TRIM25, YAP1, and α-SMA expression, while decreasing p-YAP1 levels (Fig. 7F). According to the rescue experiments (Fig. 7G), TRIM25 knockdown reversed the trend of increased YAP1 and decreased p-YAP1 caused by circELP2 overexpression. The overexpression of TRIM25 reversed the trend of decreased YAP1 as well as increased p-YAP1 caused by si-circELP2. These findings indicated that circELP2 promotes the nuclear translocation of YAP1 by inducing its dephosphorylation, a process dependent on TRIM25.

So, what genes are regulated by YAP1 after entering the nucleus were explored. The high intensity of H3K27ac modification was an indicator discriminating super-enhancer from enhancers. Combined ATAC-seq and H3K27ac ChIP-seq analyses revealed the enrichment of cytoskeleton-related genes, such as myosin heavy chain 9 (MYH9) and myosin 1c (Myo1c) (Fig. 7H), suggesting that their transcription may be regulated by super-enhancers. Myo1c is a motor protein that interacts with F-actin to play a crucial role in the dynamic control of the cytoskeleton and material transport. MYH9 operates as an intracellular molecular motor that engages with actin and is essential for generating chemomechanical stresses, as well as for the subsequent rearrangement of the actin cytoskeleton. Therefore, further research was conducted to investigate whether YAP1 was involved in super-enhancer formation to promote the transcription of cytoskeleton-related genes. Given that bromodomain-containing protein 4 (BRD4) functions as a super-enhancer reader that specifically recognizes and is highly enriched at super-enhancer regions, we hypothesized that if YAP1 drives super-enhancer assembly, it should functionally recruit BRD4 to the same nuclear loci. Therefore, we examined the subcellular co-localization of YAP1 and BRD4. Immunofluorescence images demonstrated that YAP1 and BRD4 co-localized within the nucleus, and TGF-β1 treatment led to an increase in nuclear YAP1 and BRD4 expression (Fig. 7I). Co-IP experiment confirmed the specific binding between YAP1 and BRD4, and TGF-β1 treatment further enhanced this interaction (Fig. 7J). These results suggested that YAP1 promotes the formation of super-enhancer by recruiting BRD4. To determine the functional impact of super enhancers on MYH9 and Myo1c, JQ1 was applied as a super-enhancer inhibitor. The qRT-PCR and Western blot experiments results showed that the levels of MYH9 and Myo1c were significantly decreased after JQ1 treatment (Fig. 7K-L). Co-localization analyses of H3K27ac and YAP1 were performed both in the presence and absence of TGF-β1. Immunofluorescence images demonstrated that H3K27ac and YAP1 co-localized within the nucleus, and TGF-β1 treatment led to an increase in nuclear YAP1 and H3K27ac expression (Fig. 7M). Co-IP experiment confirmed the specific binding between H3K27ac and YAP1, and TGF-β1 treatment further enhanced this interaction (Fig. 7N). Gain- and loss-of-function experiment indicated that H3K27ac was heightened in the TGF-β1-treated group. Knockdown of YAP1 resulted in reduced H3K27ac level, and overexpression of YAP1 enhanced H3K27ac level (Fig. 7O). These results suggested that YAP1 promotes the enrichment of H3K27ac to form super-enhancer.

Next, the role of YAP1 in cytoskeletal remodeling was further examined. FITC-phalloidin staining and immunofluorescence analysis revealed that YAP1 overexpression enhanced filament aggregation and F-actin accumulation. The YAP1 knockdown alleviated the filament aggregation and F-actin accumulation (Fig. 7P, Q). qRT-PCR results demonstrated that YAP1 knockdown decreased the expression of MYH9 and Myo1c genes. However, TGF-β1 and overexpression of YAP1 promoted the expression of MYH9 and Myo1c genes (Fig. 7R). The expression levels of F-actin, MYH9, and Myo1c increased in the overexpressed YAP1 group but decreased following YAP1 knockdown (Fig. 7S). The above data showed that nuclear translocation of YAP1 promotes the formation of super-enhancer to activate the expression of cytoskeleton genes led to cytoskeleton remodeling.

### circELP2 mediates conversion of biochemical signals to mechanical forces through promoting cytoskeleton remodeling depending on YAP1

**Figure 8:**
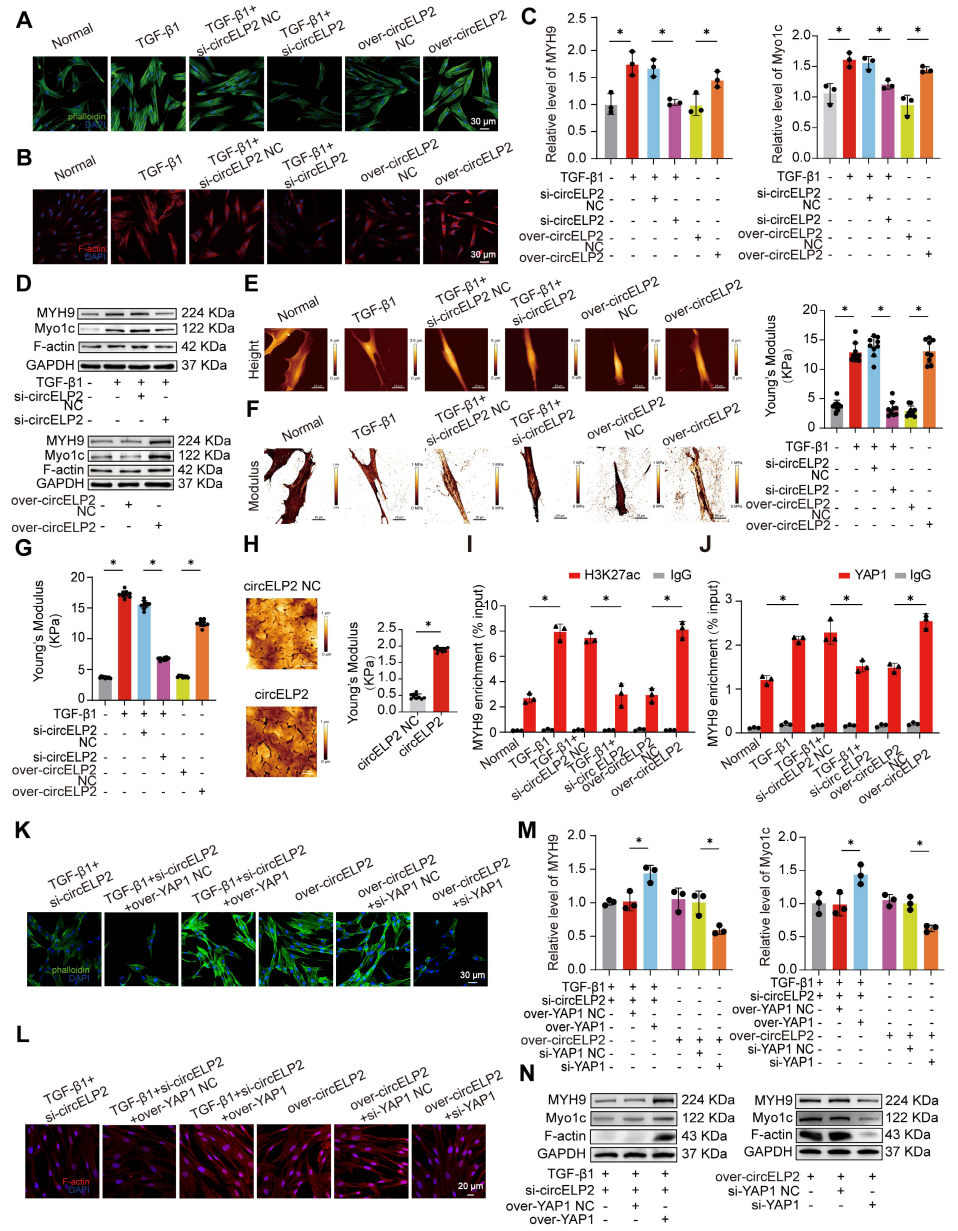
** circELP2 enhances mechanical force by promoting cytoskeletal remodeling depending on YAP1. (A)** MRC-5 cells were transfected with si-circELP2, over-circELP2 with or without TGF-β1, followed by the FITC-phalloidin experiment. The result showed that actin filament (green) intensity and aggregation were increased after circELP2 overexpression. circELP2 knockdown reduced actin filament intensity and aggregation. **(B)** The immunofluorescence assay showed that F-actin (red) accumulation was increased in the overexpressed circELP2 group, circELP2 knockdown reduced the accumulation of F-actin. **(C)** MRC-5 cells were transfected with si-circELP2, over-circELP2 with or without TGF-β1. The following qRT-PCR results showed that si-circELP2 reduced MYH9 and Myo1c levels, and circELP2 overexpression increased MYH9 and Myo1c levels (n=3). **(D)** Western blot experiment showed that F-actin, MYH9, and Myo1c levels were increased after circELP2 overexpression and decreased after circELP2 knockdown. **(E)** Representative AFM images of MRC-5 cells treated with si-circELP2, over-circELP2 with or without TGF-β1. **(F)** MRC-5 cells were transfected with si-circELP2, over-circELP2 with or without TGF-β1, followed by the detection of Youngs modulus. The Youngs modulus were increased after circELP2 overexpression and decreased after circELP2 knockdown (n=9). **(G)** Reaction forces were increased after circELP2 overexpression and decreased following circELP2 knockdown (n=9). **(H)** Representative AFM images in mice. circELP2 overexpression resulted in a high Youngs modulus value (n=10). **(I)** MRC-5 cells were transfected with si-circELP2, over-circELP2 with or without TGF-β1, followed by the ChIP-PCR assay. The result showed that TGF-β1 and circELP2 overexpression promoted binding of H3K27ac to MYH9 (n=3). **(J)** The ChIP-PCR result showed that TGF-β1 and circELP2 overexpression enhanced binding of YAP1 to MYH9 (n=3). **(K)** In MRC-5 cells, rescue experiments were conducted by transfecting si-circELP2, followed by transfection of over-YAP1, or transfecting over-circELP2, followed by transfection of si-YAP1. The following FITC-phalloidin experiment indicated that YAP1 overexpression reversed the low intensity and aggregation of actin filaments (green) induced by si-circELP2. si-YAP1 reversed the high intensity and aggregation of actin filaments caused by circELP2 overexpression. **(L)** Immunofluorescence assay of F-actin (red) in MRC-5 cells indicated that YAP1 overexpression reversed the low accumulation of F-actin induced by si-circELP2. si-YAP1 reversed the high accumulation of F-actin caused by circELP2 overexpression. **(M)** qRT-PCR results illustrated that YAP1 overexpression reversed the decreasing tendency of si-circELP2 on MYH9 and Myo1c levels. si-YAP1 reversed the increasing tendency of circELP2 overexpression on MYH9 and Myo1c levels (n=3). **(N)** Western blot experiment in MRC-5 cells illustrated that YAP1 overexpression reversed the decreased trend of F-actin, MYH9, and Myo1c caused by si-circELP2. Knockdown of YAP1 reversed the increased trend of F-actin, MYH9, and Myo1c caused by circELP2 overexpression. *p < 0.05, mean ± SD.

The cytoskeleton is capable of sensing and conducting mechanical forces, converting external mechanical signals into biochemical signals, and also converting biochemical signals into mechanical signals. The report was less on the latter. Hence, whether circELP2 mediates conversion of biochemical signals to mechanical forces through promoting cytoskeleton remodeling depending on YAP1 was further explored. FITC-phalloidin and immunofluorescence assay indicated that circELP2 overexpression enhanced filament aggregation and F-actin accumulation. The circELP2 knockdown alleviated the filament aggregation and F-actin accumulation (Fig. 8A, B). qRT-PCR results demonstrated that si-circELP2 decreased the expression of MYH9 and Myo1c genes. However, overexpression of circELP2 promoted the expression of MYH9 and Myo1c genes (Fig. 8C). The expression levels of F-actin, MYH9, and Myo1c increased in the overexpressed circELP2 group but decreased following circELP2 knockdown (Fig. 8D). The above finding showed that circELP2 can promote cytoskeleton remodeling.

The cytoskeleton orchestrates cell mechanics inside and outside of the cell and facilitates the physical integration of cells into tissues, while tissue-scale forces and extracellular rigidity govern the cell behavior. Aimed at clarifying the interaction between circELP2 and mechanical forces, the cellular morphology was first observed under atomic force microscopy. The images of the normal group showed spindle-shaped cells with a smooth surface. Overexpression of circELP2 resulted in a rougher cellular morphology, increased cell thickness, and elongated, flattened cell shapes. We also found stress fibers arranged in parallel along the main axis of the cells. After si-circELP2 treatment, the cellular morphology improved, and the stress fibers were reduced (Fig. 8E). The values of the Youngs modulus revealed a higher mechanical force in the overexpressed circELP2 group compared with the over-circELP2 negative control (NC) group. The si-circELP2 decreased the Youngs modulus value (Fig. 8F). Then, the reaction force of the cells was measured using a colloid probe with ball-stuck pressed cells. The reaction forces were increased in the overexpressed circELP2 group and decreased after si-circELP2 treatment (Fig. 8G). The atomic force microscopy images also revealed that the overexpressed circELP2 caused the lung surface to be rough, and the Youngs modulus value, the mechanical stiffness indicator, to increase in mice (Fig. 8H). All the above data suggested that circELP2 enhances mechanical forces through promoting cytoskeleton remodeling.

Then, whether circELP2 induced cytoskeleton remodeling through the formation of YAP1-mediated super-enhancer was further explored by a series of rescue experiments. ChIP-PCR results demonstrated that TGF-β1 and overexpression of circELP2 promoted H3K27ac and YAP1 binding to the MYH9 gene. si-circELP2 inhibited H3K27ac and YAP1 binding to the MYH9 gene (Fig. 8I, J). The rescue experiment indicated that YAP1 overexpression reversed the low filament aggregation and F-actin accumulation, the decreased gene levels of MYH9 and Myo1c, and the decreased protein levels of F-actin, MYH9, and Myo1c induced by si-circELP2. Meanwhile, si-YAP1 reduced the filament aggregation and F-actin accumulation, and reversed the increased gene levels of MYH9 and Myo1c, the increased protein levels of MYH9, Myo1c, and F-actin caused by the overexpression of circELP2 (Fig. 8K-N). All the data presented indicated that YAP1 converts circELP2-mediated biochemical signals to mechanical forces through promoting cytoskeleton remodeling.

**Figure 9:**
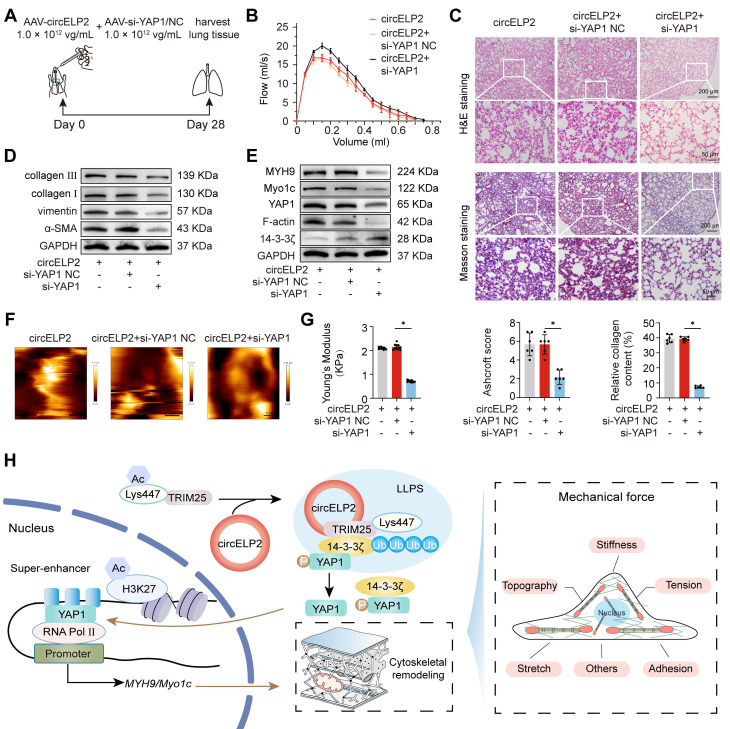
** circELP2 via YAP1 enhances mechanical forces to deteriorate pulmonary fibrosis in mice. (A)** Schematic illustration of spraying AAV into mice. AAV-circELP2 and AAV-si-YAP1/NC were packaged and sprayed into mice. The lung tissue was harvested after 28 days of AAV treatment. **(B)** Lung function assessments revealed that YAP1 knockdown alleviated the decline in lung function induced by circELP2 overexpression. **(C)** H&E staining indicated thicker alveolar walls and collagen fibers in the circELP2 overexpression group, whereas si-YAP1 significantly reversed the fibrotic characteristics induced by circELP2 overexpression. Masson staining revealed that the collagen deposition (blue) was more significant in the circELP2 overexpression group, with a marked decrease after YAP1 knockdown (n=6). **(D)** Western blot results illustrated that the expression of collagen III, collagen I, vimentin, and α-SMA were decreased in the circELP2+si-YAP1 group compared with the circELP2+si-YAP1 negative control (NC) group. **(E)** The expression of MYH9, Myo1c, F-actin, and YAP1 was decreased in the circELP2+si-YAP1 group compared with the circELP2+si-YAP1 negative control (NC) group, but the level of 14-3-3ξ was increased. **(F)** The representative image of AFM in mice treated with circELP2, circELP2+si-YAP1 negative control (NC), and circELP2+si-YAP1.** (G)** The value of Youngs modulus showed that the circELP2+si-YAP1 group had significantly lower Youngs modulus than the circELP2+si-YAP1 negative control (NC) group (n=10). **(H)** Regulatory mechanism of circELP2 in pulmonary fibrosis. *p < 0.05, mean ± SD.

Finally, the above finding was confirmed in mice. Because the circELP2 was screened from the peripheral blood samples of patients with IPF and had a low degree of homology in mice. According to the similar studies 35, 36, we first validated the mouse model is relevant to our proposed mechanism in mouse fibroblast L929 cells. The RIP assay showed that TRIM25 interacted with circELP2 after transfecting with over-circELP2 in L929 cells (Fig. S1A). Western blot showed that the levels of TRIM25, YAP1, MYH9, Myo1c, and F-actin were increased in the over-circELP2 group, the level of 14-3-3ζ had an opposite trend (Fig. S1B). The findings in L929 cells corroborated the human fibroblast results, which suggest that the mouse model is relevant to the proposed mechanism. Hence, we can use mice model to further confirm the proposed mechanism. So AAV-circELP2 and AAV-si-YAP1/NC were packaged and sprayed into mice for rescue experiments (Fig. 9A). The lung function, H&E and Masson staining, and Western blot confirmed that the lung function, lung structure, collagen deposition, and the level of fibrosis-related protein had an abnormal performance after circELP2 overexpression, whereas si-YAP1 treatment improved the condition of the lungs (Fig. 9B-D). The expression of MYH9, Myo1c, F-actin, and YAP1 was decreased by interfering with YAP1 after overexpression of circELP2 in contrast to the treatment of over-circELP2, but the level of 14-3-3ξ was increased (Fig. 9E). Frozen sections of mice lung tissues were observed by atomic force microscopy. The morphological images of mechanical force showed that the circELP2+si-YAP1 group had a smoother lung surface than the circELP2+ si-YAP1 negative control (NC) group (Fig. 9F). The value of Youngs modulus showed that the circELP2+si-YAP1 group had lower value than the circELP2+ si-YAP1 negative control (NC) group, suggesting that the tissue stiffness was significantly reduced by si-YAP1 treatment (Fig. 9G). All the above results indicated that circELP2 mediates conversion of biochemical signals to mechanical forces through promoting cytoskeleton remodeling depending on YAP1 *in vivo* and *in vitro*, leading to pulmonary fibrosis.

## Discussion

Numerous circRNAs are involved in pulmonary fibrogenesis, acting either as fibrosis inhibitors or pro-fibrotic factors, but only a limited number circRNAs have been validated by functional characterization in pulmonary fibrosis 37, 38. This study elucidated that YAP1 converts circELP2-mediated biochemical signals to mechanical forces through promoting cytoskeleton remodeling in pulmonary fibrosis. Mechanistic investigations revealed that circELP2 interacted with the 217-307 aa region of TRIM25, enhancing each other expression and stability. Then, circELP2 promoted TRIM25 deacetylation at the K447 site, which enhanced the formation of phase separation of TRIM25 via its 370-401 aa fragment. Further, the E3 ubiquitin ligase TRIM25 strengthened the ubiquitination degradation of 14-3-3ζ, leading to the targeted protein YAP1 translocation from cytoplasm to nucleus after dephosphorylation. The accumulating nuclear YAP1 mediated the formation of super-enhancer on the cytoskeleton gene locus, which initiated the transcription of cytoskeleton genes leading to the highly mechanical forces (Fig. 9H).

Phase separation can promote the formation of biomolecular condensates, including nucleoli, nucleolus plaques, and stress granules, which can rapidly enter the dense phase and take on the nature of droplets 39, 40. These structures play crucial roles in regulating important biological processes such as chromatin assembly, translation, and protein degradation 41, 42. For example, circVAMP3 promotes the formation of stress granules by interacting with the CAPRIN1 protein and inhibits the development of hepatocellular carcinoma by suppressing c-Myc translation 43. circSPECC1 enhances phase separation of ATG4B, which promotes the ubiquitination and degradation of ATG4B in gastric cancer cells 44. Despite the increasing number of studies exploring circRNAs and their interactions with RNA-binding proteins (RBPs) in phase separation, the mechanisms by which circRNAs affect this process remain underexplored. Emerging evidence suggests that RNA molecules can act as molecular scaffolds to recruit proteins and promote liquid-liquid phase separation by increasing local concentration and multivalency 45, 46. Consistent with this viewpoint, we found that circELP2 directly binds TRIM25 through its coiled-coil domain (217-307 aa), thereby facilitating TRIM25 recruitment and enrichment, which likely represents an important prerequisite for condensate formation. Notably, the RNA-binding site of TRIM25 does not overlap with its LLPS-driving region. This observation is consistent with many phase-separating systems, in which RNA binding frequently functions as an upstream recruitment or scaffolding event, whereas phase separation is primarily driven by distinct protein regions 47. Our results suggest that RNA binding promotes the phase separation process mediated by the 370-401 aa fragment of TRIM25 by increasing the local concentration of TRIM25 and enhancing multivalent interactions in pulmonary fibrosis. Moreover, post-translational modifications such as lysine acetylation have been shown to modulate the phase separation threshold and material properties of condensates 48. In our study, we demonstrate that deacetylation of TRIM25 at the K447 site mediated by circELP2 markedly enhances its phase separation capacity.

Post-translational modifications of proteins, such as acetylation/deacetylation, phosphorylation, methylation, ubiquitination, and SUMOylation, can influence the formation and stability of phase-separated structures 49. For instance, the deletion of arginine methylation in the RNA-binding protein FUS enhances its phase separation, ultimately contributing to neurodegeneration 50, 51, whereas phosphorylated histone deacetylase 6 (p-HDAC6) forms phase separation, further promoting the dysregulation of chromatin architecture in female triple-negative breast cancer 52. Nevertheless, research on the effects of acetylation on phase separation, particularly in fibrotic diseases, remains limited. The present study first identified that circELP2 promoted TRIM25 deacetylation to enhance the phase separation formation of TRIM25. TRIM25, as a protein with E3 ubiquitin ligase activity, can activates RIG-I by ubiquitinating RIG-I's CARD domain 53. Reportedly, TRIM25 ubiquitinates Keap1 to allow Nrf2 nuclear translocation, thereby inhibiting endoplasmic reticulum stress and alleviating AEC senescence 54. TRIM25 undergoes phase separation and co-condenses with the stress granule core protein G3BP1 in a dsRNA-dependent manner, which resulting in a significant enhancement of TRIM25's ubiquitination activity towards multiple antiviral proteins. This study was the first to reveal that circELP2-drived TRIM25 phase separation directly promoted the ubiquitination degradation of 14-3-3ξ, leading to YAP1 translocation from the cytoplasm to the nucleus. Nuclear YAP1 drove the super-enhancer formation on the cytoskeleton gene locus to initiate gene transcription.

Super-enhancers are clusters of enhancers that are arranged consecutively and contain greater transcription-related proteins and RNAs compared with most enhancers 55, 56. Within super-enhancer regions, there are a higher density of transcriptional activation-related histone modifications and binding to the mediator complex and bromodomain containing 4 proteins (BRD4) than do normal enhancers. Sabari *et al.* demonstrated that the transcriptional co-activators BRD4 and MED1 can phase-separate at super-enhancers to form droplets, effectively clustering the transcribed regions near the super-enhancers and facilitating a compartmentalized response to the transcription process 41. Kastano *et al.* found that the low-complexity structural domains of transcription factors form centralized hubs at super-enhancers through functionally relevant dynamic, multivalent, and sequence-specific protein-protein interactions 57. But the specific formation mechanisms have been unclear. YAP1 can act as a transcriptional coactivator to activate downstream gene expression. Herein, we first identified that YAP1 involved in the super-enhancer formation to initiate the transcription of cytoskeleton-related genes. We speculate that the enrichment of YAP1 in the super-enhancer region promote the formation of chromatin loops and enhance the interaction with promoters, thus promoting cytoskeleton gene transcription 58. The transcription of cytoskeleton-related genes induced cytoskeleton remodeling leading to increase the mechanical forces.

Cytoskeletal remodeling can cause changes in mechanical forces, which are transmitted to the surrounding extracellular matrix and neighboring cells through cell adhesion and cellular connections. Mechanical forces are sensed by cells and further affect cell differentiation, metabolism, and apoptosis 59-61. For example, the accumulation of YAP in the nucleus causes stem cells to produce "mechanical memory", which changes cytoskeletal stress and elastic deformation of the nucleus, reversing stem cell differentiation, and affecting cell stemness 62. Early micro-injuries to alveoli can increase local vascular permeability, promoting cytoskeletal remodeling and altering cellular mechanical forces. If these mild injuries are not properly repaired, fibroblasts can be activated, leading to excessive extracellular matrix deposition and resulting in pulmonary fibrosis 36. Most mechanobiology studies focus more on the conversion of mechanical forces to biological signals. But in reality, mechanical forces and biological signals interact and transform with each other, which means that the cooperation of mechanical forces inside and outside of the cell jointly controls cell fate and tissue homeostasis 63, 64. Cytoskeleton acts as a central hub for sensing mechanical and biological cues between inside and outside of the cells. Its assembly and formation are dynamically tunable depending on the physicochemical properties of the surrounding environment and partitioning biomolecules. Meanwhile, biomechanical properties of cytoskeleton can control the microenvironment for biomolecular interactions and assembly 65, 66. In other words, the cytoskeleton can both sense and generate mechanical forces, which ensures that the cell has a tunable response to mechanical forces. Nevertheless, little is known about how cytoskeleton converts the biological signals into mechanical forces. This study first revealed that YAP1 promoted the formation of super-enhancer to activate the expression of cytoskeletal remodeling genes, resulting in the elevated mechanical forces. This process was mediated by circELP2 and ultimately led to the occurrence of pulmonary fibrosis.

In conclusion, this work demonstrated that YAP1 converts circELP2-mediated biochemical signals to mechanical forces through promoting cytoskeleton remodeling leading to pulmonary fibrosis and identified this pathway as a therapeutic target for pulmonary fibrosis. These findings highlight a critical mechanism of circELP2-YAP1- cytoskeleton axis during pulmonary fibrogenesis.

## Supplementary Material

Supplementary figures and tables.

## Data Availability

All data are included in the manuscript and are available upon request by contacting the corresponding author.

## Abbreviations

IPF: Idiopathic pulmonary fibrosis
TGF-β1: Transforming growth factor-β1
circRNA: circular RNA
YAP1: Yes-associated protein 1
BLM: Bleomycin
AAV: Adeno-associated virus
qRT-PCR: Quantitative real-time PCR
H3K27ac: Acetyl-Histone H3 (Lys27)
BRD4: bromodomain-containing protein 4
RNA FISH: RNA fluorescence *in situ* hybridization
IF-FISH: immunofluorescence-fluorescence *in situ* hybridization
RIP: RNA immunoprecipitation
Co-IP: Co-Immunoprecipitation
TRIM25: Tripartite motif 25
LLPS: Liquid-liquid phase separation
IDR: Intrinsically disordered region
EGFP: Enhanced green fluorescent protein
FRAP: Fluorescence recovery after photobleaching
ChIP-PCR: Chromatin Immunoprecipitation-PCR
ATAC-seq: Assay for transposase-accessible chromatin with high-throughput sequencing
AFM: Atomic force microscopy
ROC: Receiver operating characteristic
RBPs: RNA-binding proteins
RIPA: Radioimmunoprecipitation assay
PMSF: Phenylmethylsulfonyl fluoride
NPFE: Negative pressure-driven forced expiratory
MYH9: myosin heavy chain 9
Myo1c: myosin 1c
