# Supplementary material for: YAP1 converts circELP2-mediated biochemical signals to mechanical forces through promoting cytoskeleton remodeling in pulmonary fibrosis
Source: Int J Biol Sci. 2026 Apr 8;22(8):4264–88. doi: 10.7150/ijbs.127193 (PMC13137980; doi:10.7150/ijbs.127193)
Supplement: Supplementary file 1 — Supplementary figures and tables. [file ijbsv22p4264s1.pdf]

Figure S1. (A) L929 cells were transfected with over-circELP2, followed by RIP assay. The result showed that TRIM25 interacted with circELP2. **(B)** Western blot showed that the levels of TRIM25, YAP1, MYH9, Myo1c, and F-actin were increased in the over-circELP2 group; the level of 14-3-3ζ had an opposite trend. *p* *< 0.05, mean ± SD.

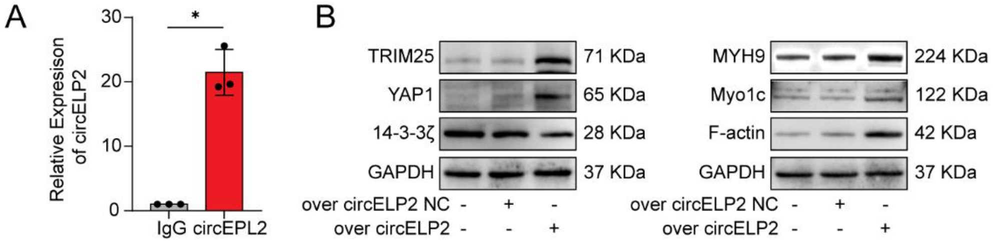

Figure S2. The vector map of the circELP2 overexpression plasmid.

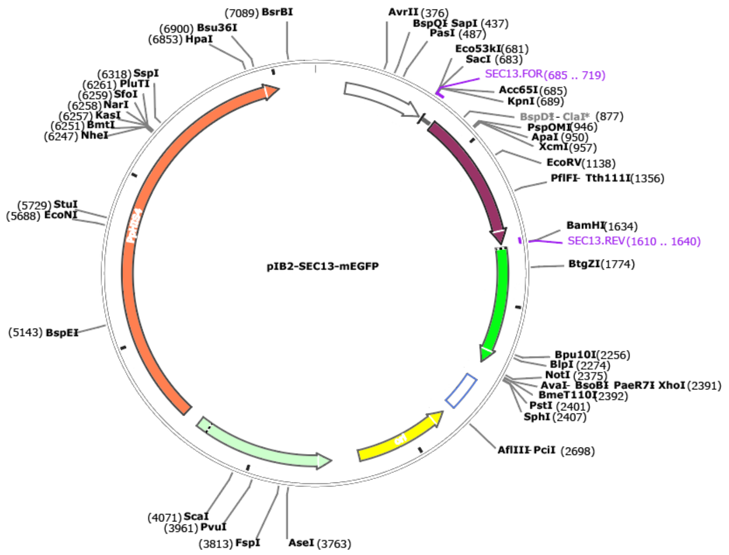

Table S1. The antibody details in the Western blot experiment.

Antibody Name	Dilution	Manufacturer	Catalog Number
anti-Collagen III	1:1000	Affinity, China	AF5457
anti-Collagen I	1:1000	Affinity, China	AF7001
anti-α-SMA	1:1000	Affinity, China	AF1032
anti-Vimentin	1:1000	Affinity, China	AF7013
anti-GAPDH	1:1000	Affinity, China	AF7021
anti-FAP	1:1000	Cell Signaling Technology, USA	66562
anti-S100A4	1:20000	Proteintech, China	16105-1-AP
anti-TRIM25	1:20000	Proteintech, China	12573-1-AP
anti-14-3-3ζ	1:5000	Proteintech, China	14881-1-AP
anti-p-YAP1	1:1000	Affinity, China	AF3328
anti- YAP1	1:5000	Proteintech, China	13584-1-AP
anti-BRD4	1:1000	Cell Signaling Technology, USA	63759
anti-H3K27ac	1:2000	ABclonal, China	A7253

anti- Acetylation	1:1000	PTM Bio, China	PTM-102
anti-LaminB1	1:10000	Bioswamp, China	RMAB60267
anti-β-Tubulin	1:2000	Affinity, China	AF7011
anti-MYH9	1:20000	Proteintech, China	11128-1-AP
anti-Myo1c	1:1000	Bioswmp, China	PAB53130
anti-F-actin	1:500	Abcam, UK	Ab205
HRP Goat Anti-Rabbit IgG	1:5000	Bioswamp, China	SAB48169
HRP Goat Anti-Mouse IgG	1:5000	Bioswamp, China	SAB48168

Table S2. The protein peptide information bound to circELP2 identified by mass spectrometry.

Accession	Gene Nan	Description	Mass	Score	Matches	Sequences	emPAI	Coverage	
P04264	KRT1	Keratin, type II cytoskeletal 1 OS=H	66170	9440	329(259)	33(27)	4.45	52%	P04264_uniprot
P13645	KRT10	Keratin, type I cytoskeletal 10 OS=H	59020	6132	240(181)	25(23)	3.32	38%	P13645_uniprot
P35908	KRT2	Keratin, type II cytoskeletal 2 epide	65678	2643	104(82)	18(17)	1.41	27%	P35908_uniprot
P35579	MYH9	Myosin-9 OS=Homo sapiens OX=9	227646	2283	156(98)	63(51)	1.31	29%	P35579_uniprot
P35527	KRT9	Keratin, type I cytoskeletal 9 OS=H	62255	1734	86(61)	17(14)	1.66	35%	P35527_uniprot
P08779	KRT16	Keratin, type I cytoskeletal 16 OS=H	51578	1594	79(49)	20(16)	2.24	46%	P08779_uniprot
P04259	KRT6B	Keratin, type II cytoskeletal 6B OS=	60315	1347	76(50)	18(13)	1.00	30%	P04259_uniprot
P02533	KRT14	Keratin, type I cytoskeletal 14 OS=H	51872	1300	58(45)	17(13)	1.37	37%	P02533_uniprot
P60709	ACTB	Actin, cytoplasmic 1 OS=Homo sap	42052	1281	72(56)	7(7)	1.67	23%	P60709_uniprot
P02538	KRT6A	Keratin, type II cytoskeletal 6A OS=	60293	1137	86(50)	23(16)	1.34	36%	P02538_uniprot
P41252	IARS	Isoleucine--tRNA ligase, cytoplasm	145718	1113	87(49)	28(22)	0.74	23%	P41252_uniprot
Q7Z794	KRT77	Keratin, type II cytoskeletal 1b OS=	62149	1098	43(32)	8(6)	0.36	14%	Q7Z794_uniprot
P48668	KRT6C	Keratin, type II cytoskeletal 6C OS=	60273	1028	80(46)	20(15)	1.22	32%	P48668_uniprot
Q04695	KRT17	Keratin, type I cytoskeletal 17 OS=H	48361	916	46(35)	15(12)	1.21	30%	Q04695_uniprot
P13647	KRT5	Keratin, type II cytoskeletal 5 OS=H	62568	885	72(46)	24(19)	1.64	37%	P13647_uniprot
P54136	RARS	Arginine--tRNA ligase, cytoplasmic	76129	805	63(37)	25(19)	1.32	41%	P54136_uniprot
P15924	DSP	Desmoplakin OS=Homo sapiens OX	334021	730	83(35)	46(23)	0.26	17%	P15924_uniprot
P11142	HSPA8	Heat shock cognate 71 kDa protein (	71082	671	57(22)	21(11)	0.97	33%	P11142_uniprot
P62805	HIST1H4A	Histone H4 OS=Homo sapiens OX=	11360	666	33(25)	5(4)	2.77	41%	P62805_uniprot
P47929	LGALS7	Galectin-7 OS=Homo sapiens OX=9	15123	579	36(16)	6(5)	2.38	51%	P47929_uniprot
P48594	SERPINB4	Serpin B4 OS=Homo sapiens OX=9	44997	523	20(15)	9(7)	0.76	24%	P48594_uniprot
P04792	HSPB1	Heat shock protein beta-1 OS=Hom	22826	517	18(13)	4(4)	0.98	24%	P04792_uniprot
Q5VTE0	EEF1A1P5	Putative elongation factor 1-alpha-li	50495	510	22(15)	7(5)	0.37	16%	Q5VTE0_uniprot
P04083	ANXA1	Annexin A1 OS=Homo sapiens OX	38918	476	21(17)	9(8)	1.08	31%	P04083_uniprot
P53621	COPA	Coatomer subunit alpha OS=Homo	139797	463	49(20)	27(15)	0.48	22%	P53621_uniprot
P07355	ANXA2	Annexin A2 OS=Homo sapiens OX	38808	439	31(20)	13(12)	1.89	40%	P07355_uniprot
P11021	HSPA5	Endoplasmic reticulum chaperone B	72402	421	31(15)	16(12)	0.86	30%	P11021_uniprot
P0DMV8	HSPA1A	Heat shock 70 kDa protein 1A OS=H	70294	416	33(19)	13(11)	0.81	24%	P0DMV8_uniprot
P07900	HSP90AA	Heat shock protein HSP 90-alpha O	85006	414	30(15)	15(8)	0.35	25%	P07900_uniprot
P31947	SFN	14-3-3 protein sigma OS=Homo sap	27871	393	38(21)	8(7)	1.76	29%	P31947_uniprot

P08238	HSP90AB	Heat shock protein HSP 90-beta OS	83554	389	31(14)	16(7)	0.31	28%	P08238_uniprot
P31151	S100A7	Protein S100-A7 OS=Homo sapiens	11578	369	14(11)	4(4)	1.84	23%	P31151_uniprot
P04406	GAPDH	Glyceraldehyde-3-phosphate dehyd	36201	351	22(17)	8(8)	1.40	24%	P04406_uniprot
Q9UM54	MYO6	Unconventional myosin-VI OS=Ho	150965	349	32(18)	18(12)	0.38	15%	Q9UM54_uniprot
Q9NZT1	CALML5	Calmodulin-like protein 5 OS=Hom	15883	347	18(11)	5(3)	1.63	36%	Q9NZT1_uniprot
P02545	LMNA	Prelamin-A/C OS=Homo sapiens O	74380	344	22(11)	16(9)	0.61	28%	P02545_uniprot
Q15046	KARS	Lysine--tRNA ligase OS=Homo sap	68461	342	39(18)	17(9)	0.67	26%	Q15046_uniprot
P06702	S100A9	Protein S100-A9 OS=Homo sapiens	13291	312	20(9)	6(5)	2.94	63%	P06702_uniprot
P05109	S100A8	Protein S100-A8 OS=Homo sapiens	10885	299	16(10)	4(3)	2.01	44%	P05109_uniprot
P31944	CASP14	Caspase-14 OS=Homo sapiens OX=	27947	290	13(12)	8(7)	1.20	29%	P31944_uniprot
P29508	SERPINB	Serpin B3 OS=Homo sapiens OX=	44594	280	15(12)	9(8)	0.77	19%	P29508_uniprot
Q00610	CLTC	Clathrin heavy chain 1 OS=Homo s	193260	266	18(9)	11(8)	0.14	8%	Q00610_uniprot
P0DOX5		Immunoglobulin gamma-1 heavy ch	49926	263	30(10)	7(5)	0.67	16%	P0DOX5_uniprot
Q02413	DSG1	Desmoglein-1 OS=Homo sapiens O	114702	256	19(9)	10(8)	0.25	13%	Q02413_uniprot
P63104	YWHAZ	14-3-3 protein zeta/delta OS=Homo	27899	255	18(10)	5(3)	0.76	22%	P63104_uniprot
P08670	VIM	Vimentin OS=Homo sapiens OX=9	53676	238	22(14)	15(9)	0.81	28%	P08670_uniprot
Q9BQE3	TUBA1C	Tubulin alpha-1C chain OS=Homo	50548	230	31(12)	8(4)	0.37	22%	Q9BQE3_uniprot
P14923	JUP	Junction plakoglobin OS=Homo sa	82434	224	20(9)	10(6)	0.26	16%	P14923_uniprot
P04075	ALDOA	Fructose-bisphosphate aldolase A O	39851	220	19(8)	8(5)	0.75	32%	P04075_uniprot
Q13813	SPTAN1	Spectrin alpha chain, non-erythrocy	285163	214	34(10)	29(10)	0.13	13%	Q13813_uniprot
Q6UWP8	SBSN	Suprabasin OS=Homo sapiens OX=	60562	209	10(6)	7(4)	0.37	20%	Q6UWP8_uniprot
P81605	DCD	Dermcidin OS=Homo sapiens OX=	11391	205	6(5)	2(2)	0.69	12%	P81605_uniprot
P06733	ENO1	Alpha-enolase OS=Homo sapiens O	47481	205	17(9)	8(6)	0.50	21%	P06733_uniprot
P35580	MYH10	Myosin-10 OS=Homo sapiens OX=	229827	200	27(13)	15(7)	0.10	8%	P35580_uniprot
Q0ZGT2	NEXN	Nexilin OS=Homo sapiens OX=96	80837	192	22(10)	12(7)	0.43	13%	Q0ZGT2_uniprot
P60174	TPI1	Triosephosphate isomerase OS=Ho	31057	186	12(6)	6(4)	0.50	20%	P60174_uniprot
O43707	ACTN4	Alpha-actinin-4 OS=Homo sapiens	105245	178	23(9)	17(9)	0.40	26%	O43707_uniprot
P14618	PKM	Pyruvate kinase PKM OS=Homo sa	58470	178	21(11)	9(7)	0.73	23%	P14618_uniprot
P00338	LDHA	L-lactate dehydrogenase A chain O	36950	170	17(10)	9(6)	0.82	29%	P00338_uniprot
P62937	PPIA	Peptidyl-prolyl cis-trans isomerase	18229	164	9(7)	5(5)	1.34	22%	P62937_uniprot
P12956	XRCC6	X-ray repair cross-complementing p	70084	162	13(5)	7(4)	0.20	13%	P12956_uniprot
Q15149	PLEC	Plectin OS=Homo sapiens OX=960	533462	161	25(7)	21(6)	0.04	5%	Q15149_uniprot
P27348	YWHAQ	14-3-3 protein theta OS=Homo sapi	28032	159	13(8)	3(3)	0.57	13%	P27348_uniprot
P0DP23	CALM1	Calmodulin-1 OS=Homo sapiens O	16827	151	9(5)	3(2)	0.73	30%	P0DP23_uniprot

P62258	YWHAE	14-3-3 protein epsilon OS=Homo sa	29326	149	15(10)	5(4)	0.71	22%	P62258_uniprot
P62979	RPS27A	Ubiquitin-40S ribosomal protein S2	18296	149	10(6)	4(4)	0.97	25%	P62979_uniprot
Q86SG5	S100A7A	Protein S100-A7A OS=Homo sapie	11412	143	6(4)	2(2)	1.20	21%	Q86SG5_uniprot
P07476	IVL	Involucrin OS=Homo sapiens OX=9	68551	142	15(7)	12(7)	0.39	27%	P07476_uniprot
P06396	GSN	Gelsolin OS=Homo sapiens OX=96	86043	134	12(5)	6(4)	0.16	13%	P06396_uniprot
P61981	YWHAG	14-3-3 protein gamma OS=Homo sa	28456	132	13(9)	4(4)	0.74	16%	P61981_uniprot
P30086	PEBP1	Phosphatidylethanolamine-binding	21158	131	4(3)	2(2)	0.34	18%	P30086_uniprot
Q13835	PKP1	Plakophilin-1 OS=Homo sapiens O	84119	130	11(3)	6(2)	0.08	10%	Q13835_uniprot
P36952	SERPINB	Serpin B5 OS=Homo sapiens OX=9	42530	129	5(4)	4(3)	0.35	14%	P36952_uniprot
Q14258	TRIM25	E3 ubiquitin/ISG15 ligase TRIM25	72581	127	8(5)	6(5)	0.25	11%	Q14258_uniprot
P01834	IGKC	Immunoglobulin kappa constant OS	11929	125	13(5)	2(2)	0.66	35%	P01834_uniprot
P07339	CTSD	Cathepsin D OS=Homo sapiens OX	45037	125	12(6)	5(4)	0.33	14%	P07339_uniprot
P23396	RPS3	40S ribosomal protein S3 OS=Hom	26842	125	12(5)	7(3)	0.42	30%	P23396_uniprot
Q9UHB6	LIMA1	LIM domain and actin-binding prot	85630	122	13(5)	9(4)	0.30	15%	Q9UHB6_uniprot
P13639	EEF2	Elongation factor 2 OS=Homo sapi	96246	122	11(8)	8(5)	0.18	8%	P13639_uniprot
Q06830	PRDX1	Peroxiredoxin-1 OS=Homo sapiens	22324	121	11(6)	4(4)	0.75	22%	Q06830_uniprot
E9PAV3	NACA	Nascent polypeptide-associated con	205979	119	4(3)	2(2)	0.03	1%	E9PAV3_uniprot
P00558	PGK1	Phosphoglycerate kinase 1 OS=Hon	44985	116	10(4)	4(2)	0.33	12%	P00558_uniprot
P04080	CSTB	Cystatin-B OS=Homo sapiens OX=	11190	114	7(4)	2(2)	1.24	24%	P04080_uniprot
Q07020	RPL18	60S ribosomal protein L18 OS=Hor	21735	114	4(2)	3(2)	0.33	19%	Q07020_uniprot
P07237	P4HB	Protein disulfide-isomerase OS=Ho	57480	113	25(7)	15(6)	0.40	33%	P07237_uniprot
Q5QNW6	HIST2H2I	Histone H2B type 2-F OS=Homo sa	13912	112	9(7)	3(3)	0.93	26%	Q5QNW6_uniprot
P02768	ALB	Serum albumin OS=Homo sapiens (	71317	110	11(4)	5(2)	0.14	9%	P02768_uniprot
P10599	TXN	Thioredoxin OS=Homo sapiens OX	12015	109	4(3)	1(1)	0.29	12%	P10599_uniprot
P68371	TUBB4B	Tubulin beta-4B chain OS=Homo s	50255	104	18(5)	6(3)	0.29	17%	P68371_uniprot
P01876	IGHA1	Immunoglobulin heavy constant alp	38486	101	6(2)	3(2)	0.18	11%	P01876_uniprot
P15311	EZR	Ezrin OS=Homo sapiens OX=9606	69484	101	13(6)	6(4)	0.20	10%	P15311_uniprot
Q92876	KLK6	Kallikrein-6 OS=Homo sapiens OX	27523	101	4(4)	2(2)	0.26	13%	Q92876_uniprot
Q6KB66	KRT80	Keratin, type II cytoskeletal 80 OS=	51007	99	8(4)	8(4)	0.28	20%	Q6KB66_uniprot
Q09666	AHNAK	Neuroblast differentiation-associate	629213	98	35(4)	27(3)	0.02	6%	Q09666_uniprot
P06753	TPM3	Tropomyosin alpha-3 chain OS=Ho	32987	98	6(5)	4(3)	0.33	13%	P06753_uniprot
P09211	GSTP1	Glutathione S-transferase P OS=Ho	23569	95	8(4)	5(3)	0.49	36%	P09211_uniprot
P58107	EPPK1	Epiplakin OS=Homo sapiens OX=9	557674	93	18(3)	12(2)	0.01	3%	P58107_uniprot
P55072	VCP	Transitional endoplasmic reticulum	89950	90	12(5)	9(5)	0.24	11%	P55072_uniprot

P26640	VARS	Valine--tRNA ligase OS=Homo sap	141642	88	7(3)	7(3)	0.10	6%	P26640_uniprot
O75223	GGCT	Gamma-glutamylcyclotransferase O	21222	88	8(3)	4(3)	0.55	27%	O75223_uniprot
P27482	CALML3	Calmodulin-like protein 3 OS=Hom	16937	85	5(3)	5(3)	0.72	43%	P27482_uniprot
P07437	TUBB	Tubulin beta chain OS=Homo sapie	50095	85	22(7)	7(4)	0.38	20%	P07437_uniprot
Q00839	HNRNPU	Heterogeneous nuclear ribonucleopı	91269	83	5(3)	3(2)	0.07	4%	Q00839_uniprot
Q9H0U4	RAB1B	Ras-related protein Rab-1B OS=Hoı	22328	83	7(3)	2(1)	0.15	9%	Q9H0U4_uniprot
P20930	FLG	Filaggrin OS=Homo sapiens OX=9(	435036	80	8(3)	4(2)	0.02	1%	P20930_uniprot
Q16643	DBN1	Drebrin OS=Homo sapiens OX=96(	71842	79	7(2)	5(2)	0.09	9%	Q16643_uniprot
O43390	HNRNPR	Heterogeneous nuclear ribonucleopı	71184	76	2(2)	1(1)	0.05	1%	O43390_uniprot
P07737	PFN1	Profilin-1 OS=Homo sapiens OX=9	15216	76	5(3)	3(2)	0.50	31%	P07737_uniprot
P13489	RNH1	Ribonuclease inhibitor OS=Homo s	51766	75	6(3)	3(2)	0.13	8%	P13489_uniprot
P06576	ATP5F1B	ATP synthase subunit beta, mitocho	56525	73	13(4)	8(3)	0.19	21%	P06576_uniprot
P19971	TYMP	Thymidine phosphorylase OS=Hom	50323	73	2(2)	2(2)	0.14	6%	P19971_uniprot
P02042	HBD	Hemoglobin subunit delta OS=Hom	16159	73	6(4)	2(1)	0.21	12%	P02042_uniprot
O14950	MYL12B	Myosin regulatory light chain 12B (	19824	72	5(3)	2(2)	0.37	12%	O14950_uniprot
P60842	EIF4A1	Eukaryotic initiation factor 4A-I OS	46353	71	9(4)	5(2)	0.15	15%	P60842_uniprot
O75369	FLNB	Filamin-B OS=Homo sapiens OX=9	280157	70	21(2)	17(2)	0.02	9%	O75369_uniprot
P35268	RPL22	60S ribosomal protein L22 OS=Hor	14835	68	3(2)	2(2)	0.51	18%	P35268_uniprot
O60437	PPL	Periplakin OS=Homo sapiens OX=9	205193	66	20(3)	16(3)	0.05	10%	O60437_uniprot
Q9C075	KRT23	Keratin, type I cytoskeletal 23 OS=I	48272	66	4(1)	4(1)	0.14	12%	Q9C075_uniprot
Q96G03	PGM2	Phosphoglucomutase-2 OS=Homo s	68754	65	3(2)	3(2)	0.10	6%	Q96G03_uniprot
P23246	SFPQ	Splicing factor, proline- and glutam	76216	64	6(4)	4(3)	0.13	5%	P23246_uniprot
P61978	HNRNPK	Heterogeneous nuclear ribonucleopı	51230	64	3(2)	3(2)	0.13	8%	P61978_uniprot
P25705	ATP5F1A	ATP synthase subunit alpha, mitoch	59828	63	11(4)	7(4)	0.24	14%	P25705_uniprot
P40121	CAPG	Macrophage-capping protein OS=H	38760	63	5(2)	3(2)	0.18	10%	P40121_uniprot
P18669	PGAM1	Phosphoglycerate mutase 1 OS=Hoı	28900	62	8(2)	5(2)	0.24	28%	P18669_uniprot
P01859	IGHG2	Immunoglobulin heavy constant gar	36505	61	9(3)	4(3)	0.30	12%	P01859_uniprot
P04843	RPN1	Dolichyl-diphosphooligosaccharide-	68641	61	2(1)	2(1)	0.05	4%	P04843_uniprot
Q9UBH0	IL36RN	Interleukin-36 receptor antagonist p	17237	61	3(3)	2(2)	0.43	8%	Q9UBH0_uniprot
Q92817	EVPL	Envoplakin OS=Homo sapiens OX=	232774	60	20(3)	14(3)	0.04	9%	Q92817_uniprot
P49755	TMED10	Transmembrane emp24 domain-con	25131	60	2(1)	2(1)	0.13	10%	P49755_uniprot
Q9Y281	CFL2	Cofilin-2 OS=Homo sapiens OX=9(	18839	59	2(2)	1(1)	0.18	6%	Q9Y281_uniprot
P61626	LYZ	Lysozyme C OS=Homo sapiens OX	16982	59	3(2)	1(1)	0.20	8%	P61626_uniprot
P16402	HIST1H1I	Histone H1.3 OS=Homo sapiens O)	22336	58	45(2)	3(1)	0.32	14%	P16402_uniprot

P26038	MSN	Moesin OS=Homo sapiens OX=96(	67892	58	10(6)	6(4)	0.21	9%	P26038_uniprot
P69905	HBA1	Hemoglobin subunit alpha OS=Hon	15305	57	5(4)	3(3)	1.23	23%	P69905_uniprot
P28072	PSMB6	Proteasome subunit beta type-6 OS=	25570	56	1(1)	1(1)	0.13	4%	P28072_uniprot
Q9P2E9	RRBP1	Ribosome-binding protein 1 OS=Hc	152780	55	10(4)	8(4)	0.09	8%	Q9P2E9_uniprot
P15880	RPS2	40S ribosomal protein S2 OS=Hom	31590	55	3(1)	3(1)	0.11	10%	P15880_uniprot
Q96FJ2	DYNLL2	Dynein light chain 2, cytoplasmic O	10457	55	1(1)	1(1)	0.33	12%	Q96FJ2_uniprot
P02675	FGB	Fibrinogen beta chain OS=Homo sa	56577	54	5(2)	5(2)	0.12	15%	P02675_uniprot
P31943	HNRNPH	Heterogeneous nuclear ribonucleop	49484	54	3(3)	2(2)	0.21	5%	P31943_uniprot
Q08211	DHX9	ATP-dependent RNA helicase A OS	142181	54	13(1)	11(1)	0.02	11%	Q08211_uniprot
Q8N1N4	KRT78	Keratin, type II cytoskeletal 78 OS=	57629	54	7(2)	5(2)	0.12	12%	Q8N1N4_uniprot
P60866	RPS20	40S ribosomal protein S20 OS=Hon	13478	53	4(2)	3(2)	0.57	26%	P60866_uniprot
Q99436	PSMB7	Proteasome subunit beta type-7 OS=	30288	52	1(1)	1(1)	0.11	3%	Q99436_uniprot
O00159	MYO1C	Unconventional myosin-Ic OS=Hon	122461	52	11(2)	7(2)	0.05	8%	O00159_uniprot
P00403	MT-CO2	Cytochrome c oxidase subunit 2 OS	25719	51	2(2)	1(1)	0.13	4%	P00403_uniprot
P52272	HNRNPM	Heterogeneous nuclear ribonucleop	77749	51	6(3)	4(3)	0.13	5%	P52272_uniprot
P32119	PRDX2	Peroxiredoxin-2 OS=Homo sapiens	22049	51	3(1)	3(1)	0.15	18%	P32119_uniprot
P01040	CSTA	Cystatin-A OS=Homo sapiens OX=	11000	50	2(1)	1(1)	0.31	18%	P01040_uniprot
P30041	PRDX6	Peroxiredoxin-6 OS=Homo sapiens	25133	50	5(3)	3(1)	0.13	16%	P30041_uniprot
O15144	ARPC2	Actin-related protein 2/3 complex su	34426	50	4(1)	4(1)	0.10	13%	O15144_uniprot
Q9H1E1	RNASE7	Ribonuclease 7 OS=Homo sapiens (	17921	50	2(1)	1(1)	0.19	8%	Q9H1E1_uniprot
P51149	RAB7A	Ras-related protein Rab-7a OS=Hor	23760	50	7(2)	5(2)	0.30	30%	P51149_uniprot
P62913	RPL11	60S ribosomal protein L11 OS=Hor	20468	49	4(2)	2(1)	0.16	14%	P62913_uniprot
P42357	HAL	Histidine ammonia-lyase OS=Homo	73336	49	5(2)	3(2)	0.09	6%	P42357_uniprot
P14174	MIF	Macrophage migration inhibitory fa	12639	49	2(1)	1(1)	0.27	9%	P14174_uniprot
Q8WVV4	POF1B	Protein POF1B OS=Homo sapiens (	68878	49	9(3)	7(3)	0.15	12%	Q8WVV4_uniprot
P29401	TKT	Transketolase OS=Homo sapiens O	68519	48	5(1)	3(1)	0.05	7%	P29401_uniprot
P16050	ALOX15	Arachidonate 15-lipoxygenase OS=	75498	48	4(3)	3(3)	0.14	6%	P16050_uniprot
P22314	UBA1	Ubiquitin-like modifier-activating e	118858	48	2(1)	2(1)	0.03	2%	P22314_uniprot
P07108	DBI	Acyl-CoA-binding protein OS=Hon	10038	48	9(2)	5(2)	0.82	64%	P07108_uniprot
P17931	LGALS3	Galectin-3 OS=Homo sapiens OX=	26193	48	5(3)	4(2)	0.43	19%	P17931_uniprot
P21333	FLNA	Filamin-A OS=Homo sapiens OX=	283301	48	15(1)	12(1)	0.02	6%	P21333_uniprot
Q08188	TGM3	Protein-glutamine gamma-glutamylt	76926	48	3(1)	3(1)	0.04	4%	Q08188_uniprot
P14625	HSP90B1	Endoplasmin OS=Homo sapiens OX	92696	47	4(2)	3(2)	0.07	4%	P14625_uniprot
P00352	ALDH1A1	Retinal dehydrogenase 1 OS=Homo	55454	46	3(1)	3(1)	0.06	9%	P00352_uniprot

P62241	RPS8	40S ribosomal protein S8 OS=Hom	24475	45	6(2)	4(2)	0.29	25%	P62241_uniprot
P18085	ARF4	ADP-ribosylation factor 4 OS=Hom	20612	45	3(1)	1(1)	0.16	6%	P18085_uniprot
P51659	HSD17B4	Peroxisomal multifunctional enzym	80092	45	2(1)	2(1)	0.04	3%	P51659_uniprot
P04040	CAT	Catalase OS=Homo sapiens OX=96	59947	45	2(1)	2(1)	0.05	4%	P04040_uniprot
P52907	CAPZA1	F-actin-capping protein subunit alpl	33073	45	5(1)	3(1)	0.10	13%	P52907_uniprot
P62249	RPS16	40S ribosomal protein S16 OS=Hon	16549	45	3(2)	1(1)	0.20	6%	P62249_uniprot
Q8WWI1	LMO7	LIM domain only protein 7 OS=Ho	194002	44	8(1)	5(1)	0.02	3%	Q8WWI1_uniprot
P06748	NPM1	Nucleophosmin OS=Homo sapiens	32726	44	2(1)	1(1)	0.10	4%	P06748_uniprot
P04632	CAPNS1	Calpain small subunit 1 OS=Homo	28469	43	1(1)	1(1)	0.12	5%	P04632_uniprot
Q15907	RAB11B	Ras-related protein Rab-11B OS=H	24588	42	2(2)	1(1)	0.14	5%	Q15907_uniprot
P51572	BCAP31	B-cell receptor-associated protein 3	28031	42	3(1)	3(1)	0.12	13%	P51572_uniprot
Q6WCQ1	MPRIP	Myosin phosphatase Rho-interactin	117260	42	18(2)	16(2)	0.12	22%	Q6WCQ1_uniprot
P47897	QARS	Glutamine--tRNA ligase OS=Homo	88655	42	1(1)	1(1)	0.04	1%	P47897_uniprot
P24534	EEF1B2	Elongation factor 1-beta OS=Homo	24919	42	4(2)	3(2)	0.29	16%	P24534_uniprot
P40763	STAT3	Signal transducer and activator of tr	88810	42	4(2)	4(2)	0.08	8%	P40763_uniprot
P11940	PABPC1	Polyadenylate-binding protein 1 OS	70854	41	4(2)	3(1)	0.05	6%	P11940_uniprot
P31930	UQCRC1	Cytochrome b-c1 complex subunit 1	53297	41	3(1)	3(1)	0.06	6%	P31930_uniprot
P62857	RPS28	40S ribosomal protein S28 OS=Hon	7893	41	4(1)	2(1)	0.45	30%	P62857_uniprot
Q6ZVX7	NCCRP1	F-box only protein 50 OS=Homo sa	30942	40	2(1)	2(1)	0.11	6%	Q6ZVX7_uniprot
Q07065	CKAP4	Cytoskeleton-associated protein 4 O	66097	40	11(2)	11(2)	0.16	24%	Q07065_uniprot
P26641	EEF1G	Elongation factor 1-gamma OS=Ho	50429	40	4(2)	2(2)	0.14	5%	P26641_uniprot
P50914	RPL14	60S ribosomal protein L14 OS=Hor	23531	40	2(1)	1(1)	0.14	5%	P50914_uniprot
P29373	CRABP2	Cellular retinoic acid-binding protei	15854	39	4(1)	2(1)	0.21	13%	P29373_uniprot
O15145	ARPC3	Actin-related protein 2/3 complex su	20761	39	2(1)	1(1)	0.16	6%	O15145_uniprot
P22735	TGM1	Protein-glutamine gamma-glutamylt	90529	39	9(1)	6(1)	0.04	9%	P22735_uniprot
P62269	RPS18	40S ribosomal protein S18 OS=Hon	17708	39	2(1)	2(1)	0.19	13%	P62269_uniprot
Q8NHW5	RPLP0P6	60S acidic ribosomal protein P0-like	34514	38	2(1)	2(1)	0.10	5%	Q8NHW5_uniprot
P19338	NCL	Nucleolin OS=Homo sapiens OX=9	76625	38	1(1)	1(1)	0.04	1%	P19338_uniprot
Q02878	RPL6	60S ribosomal protein L6 OS=Hom	32765	38	2(1)	2(1)	0.10	7%	Q02878_uniprot
P38646	HSPA9	Stress-70 protein, mitochondrial OS	73920	38	7(1)	6(1)	0.04	11%	P38646_uniprot
P30050	RPL12	60S ribosomal protein L12 OS=Hor	17979	38	1(1)	1(1)	0.19	5%	P30050_uniprot
P22392	NME2	Nucleoside diphosphate kinase B O	17401	37	3(2)	2(2)	0.43	17%	P22392_uniprot
P47756	CAPZB	F-actin-capping protein subunit beta	31616	37	1(1)	1(1)	0.11	3%	P47756_uniprot
P45974	USP5	Ubiquitin carboxyl-terminal hydrola	96638	37	3(1)	3(1)	0.03	3%	P45974_uniprot

P08865	RPSA	40S ribosomal protein SA OS=Hom	32947	37	4(1)	3(1)	0.10	8%	P08865_uniprot
P18124	RPL7	60S ribosomal protein L7 OS=Hom	29264	37	7(2)	4(2)	0.38	20%	P18124_uniprot
P62917	RPL8	60S ribosomal protein L8 OS=Hom	28235	36	1(1)	1(1)	0.12	6%	P62917_uniprot
Q9BUF5	TUBB6	Tubulin beta-6 chain OS=Homo sap	50281	36	10(2)	3(2)	0.14	8%	Q9BUF5_uniprot
P06744	GPI	Glucose-6-phosphate isomerase OS=	63335	36	5(2)	4(2)	0.11	10%	P06744_uniprot
P45880	VDAC2	Voltage-dependent anion-selective c	32060	35	4(2)	3(2)	0.22	11%	P45880_uniprot
Q08554	DSC1	Desmocollin-1 OS=Homo sapiens C	101406	35	4(1)	4(1)	0.03	6%	Q08554_uniprot
P13796	LCP1	Plastin-2 OS=Homo sapiens OX=96	70814	35	7(2)	5(2)	0.09	11%	P13796_uniprot
Q13867	BLMH	Bleomycin hydrolase OS=Homo sap	53155	35	5(1)	3(1)	0.06	9%	Q13867_uniprot
A0A0B4J1	IGHV3-74	Immunoglobulin heavy variable 3-7	13002	35	1(1)	1(1)	0.26	9%	A0A0B4J1X5_uniprot
P14735	IDE	Insulin-degrading enzyme OS=Hom	118692	35	11(1)	11(1)	0.06	13%	P14735_uniprot
Q96QA5	GSDMA	Gasdermin-A OS=Homo sapiens O	49619	35	3(1)	3(1)	0.07	8%	Q96QA5_uniprot
O95197	RTN3	Reticulon-3 OS=Homo sapiens OX=	113169	35	2(1)	2(1)	0.03	2%	O95197_uniprot
P31949	S100A11	Protein S100-A11 OS=Homo sapier	11847	35	3(2)	1(1)	0.29	8%	P31949_uniprot
P08133	ANXA6	Annexin A6 OS=Homo sapiens OX	76168	34	10(2)	9(2)	0.09	13%	P08133_uniprot
Q13045	FLII	Protein flightless-1 homolog OS=H	146142	34	9(1)	8(1)	0.02	6%	Q13045_uniprot
Q01469	FABP5	Fatty acid-binding protein, epiderma	15497	34	1(1)	1(1)	0.22	8%	Q01469_uniprot
P60660	MYL6	Myosin light polypeptide 6 OS=Ho	17090	34	4(1)	3(1)	0.43	23%	P60660_uniprot
Q9NZH8	IL36G	Interleukin-36 gamma OS=Homo sa	18937	34	3(1)	2(1)	0.18	12%	Q9NZH8_uniprot
P62851	RPS25	40S ribosomal protein S25 OS=Hon	13791	33	1(1)	1(1)	0.25	8%	P62851_uniprot
P60900	PSMA6	Proteasome subunit alpha type-6 OS	27838	33	2(1)	2(1)	0.12	10%	P60900_uniprot
Q9UJ70	NAGK	N-acetyl-D-glucosamine kinase OS=	37694	33	1(1)	1(1)	0.09	3%	Q9UJ70_uniprot
Q14203	DCTN1	Dynactin subunit 1 OS=Homo sapie	142348	33	2(2)	2(2)	0.05	1%	Q14203_uniprot
P07910	HNRNPC	Heterogeneous nuclear ribonucleopı	33707	32	4(2)	3(2)	0.21	10%	P07910_uniprot
P30101	PDIA3	Protein disulfide-isomerase A3 OS=	57146	32	6(1)	6(1)	0.06	14%	P30101_uniprot
P47914	RPL29	60S ribosomal protein L29 OS=Hor	17798	32	2(1)	2(1)	0.19	17%	P47914_uniprot
P07996	THBS1	Thrombospondin-1 OS=Homo sapie	133291	32	4(1)	4(1)	0.05	4%	P07996_uniprot
P16401	HIST1H1E	Histone H1.5 OS=Homo sapiens O	22566	32	2(1)	1(1)	0.15	4%	P16401_uniprot
Q15008	PSMD6	26S proteasome non-ATPase regula	45787	31	2(1)	2(1)	0.07	6%	Q15008_uniprot
P27797	CALR	Calreticulin OS=Homo sapiens OX=	48283	31	7(1)	3(1)	0.07	12%	P27797_uniprot
P62263	RPS14	40S ribosomal protein S14 OS=Hon	16434	31	1(1)	1(1)	0.21	8%	P62263_uniprot
P0CG04	IGLC1	Immunoglobulin lambda constant 1	11512	31	2(1)	2(1)	0.30	32%	P0CG04_uniprot
P61160	ACTR2	Actin-related protein 2 OS=Homo s	45017	30	3(1)	3(1)	0.07	9%	P61160_uniprot
Q9Y6M1	IGF2BP2	Insulin-like growth factor 2 mRNA-	66195	30	1(1)	1(1)	0.05	2%	Q9Y6M1_uniprot

P22695	UQCRC2	Cytochrome b-c1 complex subunit 2	48584	30	1(1)	1(1)	0.07	3%	P22695_uniprot
P52209	PGD	6-phosphogluconate dehydrogenase	53619	29	2(1)	2(1)	0.13	6%	P52209_uniprot
Q16658	FSCN1	Fascin OS=Homo sapiens OX=960(	55123	29	1(1)	1(1)	0.06	2%	Q16658_uniprot
O15231	ZNF185	Zinc finger protein 185 OS=Homo s	74393	29	4(1)	4(1)	0.04	9%	O15231_uniprot
P07305	H1F0	Histone H1.0 OS=Homo sapiens OX	20850	29	1(1)	1(1)	0.16	6%	P07305_uniprot
P00747	PLG	Plasminogen OS=Homo sapiens OX	93247	29	1(1)	1(1)	0.04	1%	P00747_uniprot
Q9UQ80	PA2G4	Proliferation-associated protein 2G4	44101	29	1(1)	1(1)	0.07	2%	Q9UQ80_uniprot
Q9H2D6	TRIOBP	TRIO and F-actin-binding protein O	264125	28	3(1)	3(1)	0.01	1%	Q9H2D6_uniprot
P52565	ARHGDIA	Rho GDP-dissociation inhibitor 1 O	23250	28	2(1)	1(1)	0.14	7%	P52565_uniprot
P22626	HNRNPA2	Heterogeneous nuclear ribonucleopr	37464	28	2(1)	2(1)	0.09	4%	P22626_uniprot
P49862	KLK7	Kallikrein-7 OS=Homo sapiens OX	28191	28	3(1)	2(1)	0.12	9%	P49862_uniprot
P19367	HK1	Hexokinase-1 OS=Homo sapiens O	103561	28	4(1)	4(1)	0.03	4%	P19367_uniprot
Q6NXT2	H3F3C	Histone H3.3C OS=Homo sapiens (	15318	28	1(1)	1(1)	0.22	5%	Q6NXT2_uniprot
Q14134	TRIM29	Tripartite motif-containing protein 2	66478	28	9(2)	4(2)	0.10	8%	Q14134_uniprot
P0C0S8	HIST1H2A	Histone H2A type 1 OS=Homo sapi	14083	28	5(1)	2(1)	0.24	21%	P0C0S8_uniprot
P23526	AHCY	Adenosylhomocysteinase OS=Homo	48255	27	2(1)	2(1)	0.07	5%	P23526_uniprot
P59998	ARPC4	Actin-related protein 2/3 complex su	19768	27	3(1)	1(1)	0.17	6%	P59998_uniprot
P46940	IQGAP1	Ras GTPase-activating-like protein	189761	27	7(1)	6(1)	0.02	4%	P46940_uniprot
P51648	ALDH3A2	Fatty aldehyde dehydrogenase OS=I	55269	27	2(1)	1(1)	0.06	2%	P51648_uniprot
P80188	LCN2	Neutrophil gelatinase-associated lip	22745	26	2(1)	1(1)	0.15	5%	P80188_uniprot
P55084	HADHB	Trifunctional enzyme subunit beta, i	51547	26	3(1)	2(1)	0.06	4%	P55084_uniprot
Q96F07	CYFIP2	Cytoplasmic FMR1-interacting prot	150298	25	5(1)	3(1)	0.02	2%	Q96F07_uniprot
O15143	ARPC1B	Actin-related protein 2/3 complex su	41722	25	1(1)	1(1)	0.08	4%	O15143_uniprot
P05141	SLC25A5	ADP/ATP translocase 2 OS=Homo	33059	25	8(1)	5(1)	0.10	13%	P05141_uniprot
A0A0C4D	IGKV3D-7	Immunoglobulin kappa variable 3D-	13254	25	1(1)	1(1)	0.26	7%	A0A0C4DH55_uniprot
P30153	PPP2R1A	Serine/threonine-protein phosphatas	66065	25	2(1)	2(1)	0.05	5%	P30153_uniprot
P62424	RPL7A	60S ribosomal protein L7a OS=Hon	30148	24	3(1)	3(1)	0.11	13%	P62424_uniprot
Q14574	DSC3	Desmocollin-3 OS=Homo sapiens C	101218	24	8(1)	6(1)	0.03	9%	Q14574_uniprot
P36578	RPL4	60S ribosomal protein L4 OS=Homo	47953	24	3(1)	2(1)	0.07	6%	P36578_uniprot
P07858	CTSB	Cathepsin B OS=Homo sapiens OX	38766	24	2(1)	2(1)	0.09	6%	P07858_uniprot
P07384	CAPN1	Calpain-1 catalytic subunit OS=Hor	82465	24	2(0)	2(0)	0.04	3%	P07384_uniprot
P09601	HMOX1	Heme oxygenase 1 OS=Homo sapie	32798	24	3(1)	3(1)	0.10	13%	P09601_uniprot
P51991	HNRNPA3	Heterogeneous nuclear ribonucleopr	39799	23	3(1)	3(1)	0.08	10%	P51991_uniprot
O00487	PSMD14	26S proteasome non-ATPase regula	34726	23	1(1)	1(1)	0.10	4%	O00487_uniprot

Q01518	CAP1	Adenylyl cyclase-associated protein	52325	23	3(1)	3(1)	0.06	6%	Q01518_uniprot
Q9Y5Z4	HEBP2	Heme-binding protein 2 OS=Homo	22861	23	1(1)	1(1)	0.15	6%	Q9Y5Z4_uniprot
P00491	PNP	Purine nucleoside phosphorylase OS	32325	21	9(2)	6(2)	0.34	28%	P00491_uniprot
P40939	HADHA	Trifunctional enzyme subunit alpha,	83688	21	1(0)	1(0)	0.04	1%	P40939_uniprot
P25311	AZGP1	Zinc-alpha-2-glycoprotein OS=Hom	34465	19	1(0)	1(0)	0.10	5%	P25311_uniprot
P07814	EPRS	Bifunctional glutamate/proline--tRN	172080	18	4(1)	3(1)	0.02	2%	P07814_uniprot
P61019	RAB2A	Ras-related protein Rab-2A OS=Ho	23702	17	3(0)	3(0)	0.14	19%	P61019_uniprot
P21796	VDAC1	Voltage-dependent anion-selective c	30868	17	5(0)	4(0)	0.11	19%	P21796_uniprot
P63244	RACK1	Receptor of activated protein C kina	35511	16	2(0)	2(0)	0.09	8%	P63244_uniprot

Table S3. The differentially expressed proteins bound to TRIM25 identified by mass spectrometry.

Protein IDs	Majority protein IDs	Intensity IgG	Intensity TRIM25Ab	均一化<br>Intensity IgG	均一化<br>Intensity TRIM25Ab	Fold Change<br>FC(TRIM25Ab/IgG)	sig
sp|A4D161|F221A-FAM221A_HU	sp|A4D161|F221A-FAM221A_HUMAN	0	13997000	0	1.23299E-05	#DIV/0!	up
sp|O00299|CLIC1-CLIC1_HUMAN	sp|O00299|CLIC1-CLIC1_HUMAN	0	6432300	0	5.66618E-06	#DIV/0!	up
sp|O00425|IF2B3-IGF2BP3_HUM	sp|O00425|IF2B3-IGF2BP3_HUMAN	0	109440000	0	9.64051E-05	#DIV/0!	up
sp|O00560|SDCB1-SDCBP_HUM	sp|O00560|SDCB1-SDCBP_HUMAN	0	6306800	0	5.55563E-06	#DIV/0!	up
sp|O00567|NOP56-NOP56_HUMA	sp|O00567|NOP56-NOP56_HUMAN	0	12685000	0	1.11741E-05	#DIV/0!	up
sp|O00622|CCN1-CCN1_HUMAN	sp|O00622|CCN1-CCN1_HUMAN	0	15677000	0	1.38098E-05	#DIV/0!	up
sp|O00767|ACOD-SCD_HUMAN	sp|O00767|ACOD-SCD_HUMAN	0	2910700	0	2.56402E-06	#DIV/0!	up
sp|O14579|COPE-COPE_HUMAN	sp|O14579|COPE-COPE_HUMAN	0	27112000	0	2.38828E-05	#DIV/0!	up
sp|O14908|GIPC1-GIPC1_HUMAN	sp|O14908|GIPC1-GIPC1_HUMAN	0	36486000	0	3.21403E-05	#DIV/0!	up
sp|O14974|MYPT1-PPP1R12A_HU	sp|O14974|MYPT1-PPP1R12A_HUMAN	0	24373000	0	2.147E-05	#DIV/0!	up
sp|O15460|P4HA2-P4HA2_HUMA	sp|O15460|P4HA2-P4HA2_HUMAN	0	15796000	0	1.39146E-05	#DIV/0!	up
sp|O15511|ARPC5-ARPC5_HUMA	sp|O15511|ARPC5-ARPC5_HUMAN	0	43303000	0	3.81454E-05	#DIV/0!	up
sp|O43143|DHX15-DHX15_HUM	sp|O43143|DHX15-DHX15_HUMAN	0	10760000	0	9.47842E-06	#DIV/0!	up
sp|O43175|SERA-PHGDH_HUMA	sp|O43175|SERA-PHGDH_HUMAN	0	22078000	0	1.94484E-05	#DIV/0!	up
sp|O43293|DAPK3-DAPK3_HUM	sp|O43293|DAPK3-DAPK3_HUMAN	0	5205800	0	4.58576E-06	#DIV/0!	up
sp|O43491|E41L2-EPB41L2_HUM	sp|O43491|E41L2-EPB41L2_HUMAN	0	154430000	0	0.000136037	#DIV/0!	up
sp|O43719|HTSF1-HTATSF1_HU	sp|O43719|HTSF1-HTATSF1_HUMAN	0	133410000	0	0.00011752	#DIV/0!	up
sp|O60506|HNRPQ-SYNCRIP_HU	sp|O60506|HNRPQ-SYNCRIP_HUMAN	0	27702000	0	2.44025E-05	#DIV/0!	up
sp|O60832|DKC1-DKC1_HUMAN	sp|O60832|DKC1-DKC1_HUMAN	0	17051000	0	1.50201E-05	#DIV/0!	up
sp|O75044|SRGP2-SRGAP2_HUM	sp|O75044|SRGP2-SRGAP2_HUMAN;sp|Q	0	3868200	0	3.40748E-06	#DIV/0!	up
sp|O75083|WDR1-WDR1_HUMAN	sp|O75083|WDR1-WDR1_HUMAN	0	64444000	0	5.67684E-05	#DIV/0!	up
sp|O75533|SF3B1-SF3B1_HUMAN	sp|O75533|SF3B1-SF3B1_HUMAN	0	942260000	0	0.000830032	#DIV/0!	up
sp|O75592|MYCB2-MYCBP2_HU	sp|O75592|MYCB2-MYCBP2_HUMAN	0	4025800	0	3.5463E-06	#DIV/0!	up
sp|O75643|U520-SNRNP200_HUM	sp|O75643|U520-SNRNP200_HUMAN	0	1847800	0	1.62772E-06	#DIV/0!	up
sp|O75962|TRIO-TRIO_HUMAN;s	sp|O75962|TRIO-TRIO_HUMAN;sp|O6022	0	7053600	0	6.21348E-06	#DIV/0!	up
sp|O76021|RL1D1-RSL1D1_HUM	sp|O76021|RL1D1-RSL1D1_HUMAN	0	28415000	0	2.50306E-05	#DIV/0!	up
sp|O94851|MICA2-MICAL2_HUM	sp|O94851|MICA2-MICAL2_HUMAN	0	4772400	0	4.20398E-06	#DIV/0!	up
sp|O94906|PRP6-PRPF6_HUMAN	sp|O94906|PRP6-PRPF6_HUMAN	0	2011400	0	1.77183E-06	#DIV/0!	up
sp|O94929|ABLM3-ABLIM3_HUM	sp|O94929|ABLM3-ABLIM3_HUMAN	0	16611000	0	1.46325E-05	#DIV/0!	up
sp|O95573|ACSL3-ACSL3_HUMA	sp|O95573|ACSL3-ACSL3_HUMAN	0	26539000	0	2.33781E-05	#DIV/0!	up
sp|O95749|GGPPS-GGPS1_HUMA	sp|O95749|GGPPS-GGPS1_HUMAN	0	8497300	0	7.48522E-06	#DIV/0!	up
sp|O95782|AP2A1-AP2A1_HUMA	sp|O95782|AP2A1-AP2A1_HUMAN;sp|O94	0	26914000	0	2.37084E-05	#DIV/0!	up
sp|O95810|CAVN2-CAVIN2_HUM	sp|O95810|CAVN2-CAVIN2_HUMAN	0	20466000	0	1.80284E-05	#DIV/0!	up
sp|O95816|BAG2-BAG2_HUMAN	sp|O95816|BAG2-BAG2_HUMAN	0	35221000	0	3.1026E-05	#DIV/0!	up
sp|O95864|FADS2-FADS2_HUMA	sp|O95864|FADS2-FADS2_HUMAN	0	53153000	0	4.68222E-05	#DIV/0!	up
sp|P00367|DHE3-GLUD1_HUMAN	sp|P00367|DHE3-GLUD1_HUMAN;sp|P494	0	30396000	0	2.67757E-05	#DIV/0!	up
sp|P01037|CYTN-CST1_HUMAN	sp|P01037|CYTN-CST1_HUMAN	0	4311300	0	3.7978E-06	#DIV/0!	up
sp|P02461|CO3A1-COL3A1_HUM	sp|P02461|CO3A1-COL3A1_HUMAN;CON	0	7176100	0	6.32139E-06	#DIV/0!	up

sp|P04156|PRIO-PRNP_HUMAN	sp|P04156|PRIO-PRNP_HUMAN	0	4042600	0	3.5611E-06	#DIV/0!	up
sp|P05141|ADT2-SLC25A5_HUM/	sp|P05141|ADT2-SLC25A5_HUMAN	0	26696000	0	2.35164E-05	#DIV/0!	up
sp|P05386|RLA1-RPLP1_HUMAN	sp|P05386|RLA1-RPLP1_HUMAN	0	18000000	0	1.58561E-05	#DIV/0!	up
sp|P06748|NPM-NPM1_HUMAN	sp|P06748|NPM-NPM1_HUMAN	0	108020000	0	9.51542E-05	#DIV/0!	up
sp|P07195|LDHB-LDHB_HUMAN	sp|P07195|LDHB-LDHB_HUMAN	0	38977000	0	3.43346E-05	#DIV/0!	up
sp|P07737|PROF1-PFN1_HUMAN	sp|P07737|PROF1-PFN1_HUMAN	0	4755500	0	4.18909E-06	#DIV/0!	up
sp|P07910|HNRPC-HNRNPC_HUI	sp|P07910|HNRPC-HNRNPC_HUMAN	0	22926000	0	2.01954E-05	#DIV/0!	up
sp|P07996|TSP1-THBS1_HUMAN;	sp|P07996|TSP1-THBS1_HUMAN	0	18227000	0	1.60561E-05	#DIV/0!	up
sp|P08195|4F2-SLC3A2_HUMAN	sp|P08195|4F2-SLC3A2_HUMAN	0	5950700	0	5.24194E-06	#DIV/0!	up
sp|P08237|PFKAM-PFKM_HUMA	sp|P08237|PFKAM-PFKM_HUMAN	0	5585800	0	4.9205E-06	#DIV/0!	up
sp|P08579|RU2B-SNRPB2_HUMA	sp|P08579|RU2B-SNRPB2_HUMAN	0	9513300	0	8.38021E-06	#DIV/0!	up
sp|P08708|RS17-RPS17_HUMAN	sp|P08708|RS17-RPS17_HUMAN	0	200520000	0	0.000176637	#DIV/0!	up
sp|P08754|GNAI3-GNAI3_HUMAI	sp|P08754|GNAI3-GNAI3_HUMAN	0	22094000	0	1.94625E-05	#DIV/0!	up
sp|P09382|LEG1-LGALS1_HUMA	sp|P09382|LEG1-LGALS1_HUMAN	0	129360000	0	0.000113953	#DIV/0!	up
sp|P09661|RU2A-SNRPA1_HUMA	sp|P09661|RU2A-SNRPA1_HUMAN	0	73412000	0	6.46682E-05	#DIV/0!	up
sp|P09936|UCHL1-UCHL1_HUM/	sp|P09936|UCHL1-UCHL1_HUMAN	0	5080700	0	4.47556E-06	#DIV/0!	up
sp|P0CG39|POTEJ-POTEJ_HUMA	sp|P0CG39|POTEJ-POTEJ_HUMAN	0	10013000	0	8.8204E-06	#DIV/0!	up
sp|P11047|LAMC1-LAMC1_HUM.	sp|P11047|LAMC1-LAMC1_HUMAN	0	3556000	0	3.13246E-06	#DIV/0!	up
sp|P11387|TOP1-TOP1_HUMAN;s	sp|P11387|TOP1-TOP1_HUMAN	0	27670000	0	2.43743E-05	#DIV/0!	up
sp|P12110|CO6A2-COL6A2_HUM.	sp|P12110|CO6A2-COL6A2_HUMAN	0	68740000	0	6.05527E-05	#DIV/0!	up
sp|P13010|XRCC5-XRCC5_HUM/	sp|P13010|XRCC5-XRCC5_HUMAN	0	123760000	0	0.000109019	#DIV/0!	up
sp|P13489|RINI-RNH1_HUMAN	sp|P13489|RINI-RNH1_HUMAN	0	13297000	0	1.17133E-05	#DIV/0!	up
sp|P13674|P4HA1-P4HA1_HUMAI	sp|P13674|P4HA1-P4HA1_HUMAN	0	67218000	0	5.9212E-05	#DIV/0!	up
sp|P13797|PLST-PLS3_HUMAN;s	sp|P13797|PLST-PLS3_HUMAN;sp|P13796|	0	4527400	0	3.98816E-06	#DIV/0!	up
sp|P13987|CD59-CD59_HUMAN	sp|P13987|CD59-CD59_HUMAN	0	102790000	0	9.05471E-05	#DIV/0!	up
sp|P14649|MYL6B-MYL6B_HUM.	sp|P14649|MYL6B-MYL6B_HUMAN	0	82490000	0	7.2665E-05	#DIV/0!	up
sp|P15144|AMPN-ANPEP_HUMAI	sp|P15144|AMPN-ANPEP_HUMAN	0	55702000	0	4.90676E-05	#DIV/0!	up
sp|P15259|PGAM2-PGAM2_HUM.	sp|P15259|PGAM2-PGAM2_HUMAN;sp|P1	0	2987100	0	2.63132E-06	#DIV/0!	up
sp|P15880|RS2-RPS2_HUMAN	sp|P15880|RS2-RPS2_HUMAN	0	104870000	0	9.23794E-05	#DIV/0!	up
sp|P16070|CD44-CD44_HUMAN	sp|P16070|CD44-CD44_HUMAN	0	19771000	0	1.74162E-05	#DIV/0!	up
sp|P16520|GBB3-GNB3_HUMAN	sp|P16520|GBB3-GNB3_HUMAN	0	13855000	0	1.22048E-05	#DIV/0!	up
sp|P16615|AT2A2-ATP2A2_HUM/	sp|P16615|AT2A2-ATP2A2_HUMAN;sp|Q9	0	4276400	0	3.76706E-06	#DIV/0!	up
sp|P17302|CXA1-GJA1_HUMAN	sp|P17302|CXA1-GJA1_HUMAN	0	11591000	0	1.02104E-05	#DIV/0!	up
sp|P17987|TCPA-TCP1_HUMAN	sp|P17987|TCPA-TCP1_HUMAN	0	14822000	0	1.30566E-05	#DIV/0!	up
sp|P18077|RL35A-RPL35A_HUM/	sp|P18077|RL35A-RPL35A_HUMAN	0	29763000	0	2.62181E-05	#DIV/0!	up
sp|P18206|VINC-VCL_HUMAN	sp|P18206|VINC-VCL_HUMAN	0	9056300	0	7.97764E-06	#DIV/0!	up
sp|P19474|RO52-TRIM21_HUMAI	sp|P19474|RO52-TRIM21_HUMAN	0	17131000	0	1.50906E-05	#DIV/0!	up
sp|P20908|CO5A1-COL5A1_HUM.	sp|P20908|CO5A1-COL5A1_HUMAN	0	15317000	0	1.34927E-05	#DIV/0!	up
sp|P21980|TGM2-TGM2_HUMAN	sp|P21980|TGM2-TGM2_HUMAN	0	89669000	0	7.89889E-05	#DIV/0!	up
sp|P22087|FBRL-FBL_HUMAN;sp	sp|P22087|FBRL-FBL_HUMAN	0	67530000	0	5.94868E-05	#DIV/0!	up
sp|P22314|UBA1-UBA1_HUMAN	sp|P22314|UBA1-UBA1_HUMAN	0	9081500	0	7.99984E-06	#DIV/0!	up
sp|P24593|IBP5-IGFBP5_HUMAN	sp|P24593|IBP5-IGFBP5_HUMAN	0	5573100	0	4.90931E-06	#DIV/0!	up
sp|P24821|TENA-TNC_HUMAN	sp|P24821|TENA-TNC_HUMAN	0	4777500	0	4.20847E-06	#DIV/0!	up

sp|P26038|MOES-MSN_HUMAN;s	sp|P26038|MOES-MSN_HUMAN	0	22062000	0	1.94343E-05	#DIV/0!	up
sp|P26599|PTBP1-PTBP1_HUMAN	sp|P26599|PTBP1-PTBP1_HUMAN	0	11603000	0	1.0221E-05	#DIV/0!	up
sp|P26641|EF1G-EEF1G_HUMAN	sp|P26641|EF1G-EEF1G_HUMAN	0	7591600	0	6.6874E-06	#DIV/0!	up
sp|P28288|ABCD3-ABCD3_HUMA	sp|P28288|ABCD3-ABCD3_HUMAN	0	7678600	0	6.76404E-06	#DIV/0!	up
sp|P28289|TMOD1-TMOD1_HUM	sp|P28289|TMOD1-TMOD1_HUMAN	0	84825000	0	7.47219E-05	#DIV/0!	up
sp|P28290|ITPI2-ITPRID2_HUMAI	sp|P28290|ITPI2-ITPRID2_HUMAN	0	21469000	0	1.89119E-05	#DIV/0!	up
sp|P28300|LYOX-LOX_HUMAN	sp|P28300|LYOX-LOX_HUMAN	0	20011000	0	1.76276E-05	#DIV/0!	up
sp|P28799|GRN-GRN_HUMAN	sp|P28799|GRN-GRN_HUMAN	0	16480000	0	1.45171E-05	#DIV/0!	up
sp|P29401|TKT-TKT_HUMAN	sp|P29401|TKT-TKT_HUMAN	0	8796500	0	7.74879E-06	#DIV/0!	up
sp|P29536|LMOD1-LMOD1_HUM	sp|P29536|LMOD1-LMOD1_HUMAN	0	62977000	0	5.54761E-05	#DIV/0!	up
sp|P30038|AL4A1-ALDH4A1_HUI	sp|P30038|AL4A1-ALDH4A1_HUMAN	0	8633400	0	7.60511E-06	#DIV/0!	up
sp|P30041|PRDX6-PRDX6_HUMA	sp|P30041|PRDX6-PRDX6_HUMAN	0	20802000	0	1.83244E-05	#DIV/0!	up
sp|P30101|PDIA3-PDIA3_HUMAN	sp|P30101|PDIA3-PDIA3_HUMAN	0	20217000	0	1.7809E-05	#DIV/0!	up
sp|P30305|MPIP2-CDC25B_HUMA	sp|P30305|MPIP2-CDC25B_HUMAN	0	31048000	0	2.735E-05	#DIV/0!	up
sp|P32969|RL9-RPL9_HUMAN	sp|P32969|RL9-RPL9_HUMAN	0	111660000	0	9.83607E-05	#DIV/0!	up
sp|P35052|GPC1-GPC1_HUMAN	sp|P35052|GPC1-GPC1_HUMAN	0	19852000	0	1.74875E-05	#DIV/0!	up
sp|P35221|CTNA1-CTNNA1_HUM	sp|P35221|CTNA1-CTNNA1_HUMAN	0	6362600	0	5.60478E-06	#DIV/0!	up
sp|P35222|CTNB1-CTNNB1_HUM	sp|P35222|CTNB1-CTNNB1_HUMAN	0	7775700	0	6.84957E-06	#DIV/0!	up
sp|P35606|COPB2-COPB2_HUMA	sp|P35606|COPB2-COPB2_HUMAN	0	38942000	0	3.43038E-05	#DIV/0!	up
sp|P35611|ADDA-ADD1_HUMAN	sp|P35611|ADDA-ADD1_HUMAN	0	10786000	0	9.50133E-06	#DIV/0!	up
sp|P35749|MYH11-MYH11_HUMA	sp|P35749|MYH11-MYH11_HUMAN	0	154930000	0	0.000136477	#DIV/0!	up
sp|P36873|PP1G-PPP1CC_HUMAN	sp|P36873|PP1G-PPP1CC_HUMAN	0	12006000	0	1.0576E-05	#DIV/0!	up
sp|P38919|IF4A3-EIF4A3_HUMAN	sp|P38919|IF4A3-EIF4A3_HUMAN	0	7394700	0	6.51395E-06	#DIV/0!	up
sp|P39880|CUX1-CUX1_HUMAN;	sp|P39880|CUX1-CUX1_HUMAN;sp|O1452	0	10368000	0	9.13311E-06	#DIV/0!	up
sp|P40227|TCPZ-CCT6A_HUMAN	sp|P40227|TCPZ-CCT6A_HUMAN	0	4408500	0	3.88342E-06	#DIV/0!	up
sp|P41252|SYIC-IARS_HUMAN	sp|P41252|SYIC-IARS_HUMAN	0	8515800	0	7.50152E-06	#DIV/0!	up
sp|P42025|ACTY-ACTR1B_HUMA	sp|P42025|ACTY-ACTR1B_HUMAN	0	13512000	0	1.19026E-05	#DIV/0!	up
sp|P42677|RS27-RPS27_HUMAN	sp|P42677|RS27-RPS27_HUMAN	0	22634000	0	1.99382E-05	#DIV/0!	up
sp|P43243|MATR3-MATR3_HUM.	sp|P43243|MATR3-MATR3_HUMAN	0	71751000	0	6.32051E-05	#DIV/0!	up
sp|P46087|NOP2-NOP2_HUMAN	sp|P46087|NOP2-NOP2_HUMAN	0	230660000	0	0.000203187	#DIV/0!	up
sp|P46777|RL5-RPL5_HUMAN	sp|P46777|RL5-RPL5_HUMAN	0	670660000	0	0.000590781	#DIV/0!	up
sp|P46783|RS10-RPS10_HUMAN;s	sp|P46783|RS10-RPS10_HUMAN;sp|Q9NQ	0	87841000	0	7.73786E-05	#DIV/0!	up
sp|P46939|UTRO-UTRN_HUMAN	sp|P46939|UTRO-UTRN_HUMAN	0	13472000	0	1.18674E-05	#DIV/0!	up
sp|P47914|RL29-RPL29_HUMAN	sp|P47914|RL29-RPL29_HUMAN	0	65453000	0	5.76572E-05	#DIV/0!	up
sp|P48651|PTSS1-PTDSS1_HUMA	sp|P48651|PTSS1-PTDSS1_HUMAN	0	25494000	0	2.24575E-05	#DIV/0!	up
sp|P48681|NEST-NES_HUMAN	sp|P48681|NEST-NES_HUMAN	0	12895000	0	1.13591E-05	#DIV/0!	up
sp|P49207|RL34-RPL34_HUMAN	sp|P49207|RL34-RPL34_HUMAN	0	65199000	0	5.74334E-05	#DIV/0!	up
sp|P49368|TCPG-CCT3_HUMAN	sp|P49368|TCPG-CCT3_HUMAN	0	15108000	0	1.33086E-05	#DIV/0!	up
sp|P50395|GDIB-GDI2_HUMAN;s	sp|P50395|GDIB-GDI2_HUMAN;sp|P31150|	0	4088700	0	3.60171E-06	#DIV/0!	up
sp|P50402|EMD-EMD_HUMAN	sp|P50402|EMD-EMD_HUMAN	0	22749000	0	2.00395E-05	#DIV/0!	up
sp|P50479|PDLI4-PDLIM4_HUMA	sp|P50479|PDLI4-PDLIM4_HUMAN	0	10847000	0	9.55506E-06	#DIV/0!	up
sp|P51114|FXR1-FXR1_HUMAN	sp|P51114|FXR1-FXR1_HUMAN	0	94040000	0	8.28393E-05	#DIV/0!	up
sp|P51116|FXR2-FXR2_HUMAN	sp|P51116|FXR2-FXR2_HUMAN	0	21717000	0	1.91304E-05	#DIV/0!	up

sp|P51571|SSRD-SSR4_HUMAN	sp|P51571|SSRD-SSR4_HUMAN	0	2817500	0	2.48192E-06	#DIV/0!	up
sp|P51991|ROA3-HNRNPA3_HUM	sp|P51991|ROA3-HNRNPA3_HUMAN	0	4382100	0	3.86017E-06	#DIV/0!	up
sp|P52209|6PGD-PGD_HUMAN	sp|P52209|6PGD-PGD_HUMAN	0	24427000	0	2.15176E-05	#DIV/0!	up
sp|P52943|CRIP2-CRIP2_HUMAN	sp|P52943|CRIP2-CRIP2_HUMAN	0	5841100	0	5.14539E-06	#DIV/0!	up
sp|P53007|TXTP-SLC25A1_HUMA	sp|P53007|TXTP-SLC25A1_HUMAN	0	72036000	0	6.34561E-05	#DIV/0!	up
sp|P53396|ACLY-ACLY_HUMAN	sp|P53396|ACLY-ACLY_HUMAN	0	61797000	0	5.44366E-05	#DIV/0!	up
sp|P53618|COPB-COPB1_HUMAN	sp|P53618|COPB-COPB1_HUMAN	0	17127000	0	1.50871E-05	#DIV/0!	up
sp|P54289|CA2D1-CACNA2D1_HU	sp|P54289|CA2D1-CACNA2D1_HUMAN	0	15444000	0	1.36045E-05	#DIV/0!	up
sp|P55209|NP1L1-NAP1L1_HUMA	sp|P55209|NP1L1-NAP1L1_HUMAN;sp|Q9	0	6787600	0	5.97916E-06	#DIV/0!	up
sp|P55265|DSRAD-ADAR_HUMA	sp|P55265|DSRAD-ADAR_HUMAN	0	11046000	0	9.73036E-06	#DIV/0!	up
sp|P55884|EIF3B-EIF3B_HUMAN	sp|P55884|EIF3B-EIF3B_HUMAN	0	7451900	0	6.56434E-06	#DIV/0!	up
sp|P56211|ARP19-ARPP19_HUMA	sp|P56211|ARP19-ARPP19_HUMAN	0	37399000	0	3.29446E-05	#DIV/0!	up
sp|P61513|RL37A-RPL37A_HUMA	sp|P61513|RL37A-RPL37A_HUMAN	0	54460000	0	4.79735E-05	#DIV/0!	up
sp|P61619|S61A1-SEC61A1_HUM	sp|P61619|S61A1-SEC61A1_HUMAN	0	15002000	0	1.32152E-05	#DIV/0!	up
sp|P61978|HNRPK-HNRNPK_HUM	sp|P61978|HNRPK-HNRNPK_HUMAN	0	14808000	0	1.30443E-05	#DIV/0!	up
sp|P61981|1433G-YWHAG_HUMA	sp|P61981|1433G-YWHAG_HUMAN	0	9707900	0	8.55164E-06	#DIV/0!	up
sp|P62136|PP1A-PPP1CA_HUMAN	sp|P62136|PP1A-PPP1CA_HUMAN	0	11932000	0	1.05108E-05	#DIV/0!	up
sp|P62266|RS23-RPS23_HUMAN	sp|P62266|RS23-RPS23_HUMAN	0	99378000	0	8.75415E-05	#DIV/0!	up
sp|P62273|RS29-RPS29_HUMAN	sp|P62273|RS29-RPS29_HUMAN	0	10291000	0	9.06528E-06	#DIV/0!	up
sp|P62306|RUXF-SNRPF_HUMAN	sp|P62306|RUXF-SNRPF_HUMAN	0	3619300	0	3.18822E-06	#DIV/0!	up
sp|P62314|SMD1-SNRPD1_HUMA	sp|P62314|SMD1-SNRPD1_HUMAN	0	36657000	0	3.22909E-05	#DIV/0!	up
sp|P62316|SMD2-SNRPD2_HUMA	sp|P62316|SMD2-SNRPD2_HUMAN	0	74886000	0	6.59667E-05	#DIV/0!	up
sp|P62318|SMD3-SNRPD3_HUMA	sp|P62318|SMD3-SNRPD3_HUMAN	0	57389000	0	5.05537E-05	#DIV/0!	up
sp|P62750|RL23A-RPL23A_HUMA	sp|P62750|RL23A-RPL23A_HUMAN	0	263060000	0	0.000231728	#DIV/0!	up
sp|P62857|RS28-RPS28_HUMAN	sp|P62857|RS28-RPS28_HUMAN	0	10059000	0	8.86092E-06	#DIV/0!	up
sp|P62861|RS30-FAU_HUMAN	sp|P62861|RS30-FAU_HUMAN	0	17634000	0	1.55337E-05	#DIV/0!	up
sp|P62873|GBB1-GNB1_HUMAN	sp|P62873|GBB1-GNB1_HUMAN	0	104160000	0	9.1754E-05	#DIV/0!	up
sp|P62879|GBB2-GNB2_HUMAN;	sp|P62879|GBB2-GNB2_HUMAN;sp|Q9HA	0	66720000	0	5.87733E-05	#DIV/0!	up
sp|P62937|PPIA-PPIA_HUMAN	sp|P62937|PPIA-PPIA_HUMAN	0	17644000	0	1.55425E-05	#DIV/0!	up
sp|P63010|AP2B1-AP2B1_HUMAN	sp|P63010|AP2B1-AP2B1_HUMAN;sp|Q10	0	20307000	0	1.78883E-05	#DIV/0!	up
sp|P63096|GNAI1-GNAI1_HUMAN	sp|P63096|GNAI1-GNAI1_HUMAN	0	13162000	0	1.15943E-05	#DIV/0!	up
sp|P63162|RSMN-SNRPN_HUMAN	sp|P63162|RSMN-SNRPN_HUMAN;sp|P14	0	24614000	0	2.16823E-05	#DIV/0!	up
sp|P63167|DYL1-DYNLL1_HUMA	sp|P63167|DYL1-DYNLL1_HUMAN	0	12452000	0	1.09689E-05	#DIV/0!	up
sp|P63173|RL38-RPL38_HUMAN	sp|P63173|RL38-RPL38_HUMAN	0	14595000	0	1.28567E-05	#DIV/0!	up
sp|P67809|YBOX1-YBX1_HUMAN	sp|P67809|YBOX1-YBX1_HUMAN	0	101450000	0	8.93667E-05	#DIV/0!	up
sp|P67870|CSK2B-CSNK2B_HUM	sp|P67870|CSK2B-CSNK2B_HUMAN	0	8410600	0	7.40885E-06	#DIV/0!	up
sp|P68032|ACTC-ACTC1_HUMAN	sp|P68032|ACTC-ACTC1_HUMAN;sp|P681	0	880830000	0	0.000775918	#DIV/0!	up
sp|P78371|TCPB-CCT2_HUMAN	sp|P78371|TCPB-CCT2_HUMAN	0	5253100	0	4.62743E-06	#DIV/0!	up
sp|P78524|ST5-ST5_HUMAN	sp|P78524|ST5-ST5_HUMAN	0	36975000	0	3.25711E-05	#DIV/0!	up
sp|P78559|MAP1A-MAP1A_HUM	sp|P78559|MAP1A-MAP1A_HUMAN;sp|P4	0	3120000	0	2.74839E-06	#DIV/0!	up
sp|P80723|BASP1-BASP1_HUMAN	sp|P80723|BASP1-BASP1_HUMAN	0	21002000	0	1.85005E-05	#DIV/0!	up
sp|P83111|LACTB-LACTB_HUMA	sp|P83111|LACTB-LACTB_HUMAN	0	119980000	0	0.00010569	#DIV/0!	up
sp|Q00341|VIGLN-HDLBP_HUMA	sp|Q00341|VIGLN-HDLBP_HUMAN	0	55572000	0	4.89531E-05	#DIV/0!	up

sp|Q00577|PURA-PURA_HUMAN	sp|Q00577|PURA-PURA_HUMAN	0	22987000	0	2.02491E-05	#DIV/0!	up
sp|Q01813|PFKAP-PFKP_HUMAN	sp|Q01813|PFKAP-PFKP_HUMAN	0	94882000	0	8.3581E-05	#DIV/0!	up
sp|Q01995|TAGL-TAGLN_HUMA	sp|Q01995|TAGL-TAGLN_HUMAN	0	16571000	0	1.45973E-05	#DIV/0!	up
sp|Q02880|TOP2B-TOP2B_HUMA	sp|Q02880|TOP2B-TOP2B_HUMAN	0	10050000	0	8.85299E-06	#DIV/0!	up
sp|Q02978|M2OM-SLC25A11_HU	sp|Q02978|M2OM-SLC25A11_HUMAN	0	7076200	0	6.23339E-06	#DIV/0!	up
sp|Q06210|GFPT1-GFPT1_HUMA	sp|Q06210|GFPT1-GFPT1_HUMAN	0	3954700	0	3.48367E-06	#DIV/0!	up
sp|Q07955|SRSF1-SRSF1_HUMAN	sp|Q07955|SRSF1-SRSF1_HUMAN	0	46871000	0	4.12884E-05	#DIV/0!	up
sp|Q12797|ASPH-ASPH_HUMAN	sp|Q12797|ASPH-ASPH_HUMAN	0	44429000	0	3.91373E-05	#DIV/0!	up
sp|Q12874|SF3A3-SF3A3_HUMAN	sp|Q12874|SF3A3-SF3A3_HUMAN	0	900180000	0	0.000792964	#DIV/0!	up
sp|Q12906|ILF3-ILF3_HUMAN;sp|	sp|Q12906|ILF3-ILF3_HUMAN	0	105110000	0	9.25908E-05	#DIV/0!	up
sp|Q13162|PRDX4-PRDX4_HUMA	sp|Q13162|PRDX4-PRDX4_HUMAN	0	6809500	0	5.99845E-06	#DIV/0!	up
sp|Q13200|PSMD2-PSMD2_HUMA	sp|Q13200|PSMD2-PSMD2_HUMAN	0	12512000	0	1.10218E-05	#DIV/0!	up
sp|Q13243|SRSF5-SRSF5_HUMAN	sp|Q13243|SRSF5-SRSF5_HUMAN	0	15227000	0	1.34134E-05	#DIV/0!	up
sp|Q13310|PABP4-PABPC4_HUM	sp|Q13310|PABP4-PABPC4_HUMAN	0	119870000	0	0.000105593	#DIV/0!	up
sp|Q13435|SF3B2-SF3B2_HUMAN	sp|Q13435|SF3B2-SF3B2_HUMAN	0	670570000	0	0.000590701	#DIV/0!	up
sp|Q13501|SQSTM-SQSTM1_HUM	sp|Q13501|SQSTM-SQSTM1_HUMAN	0	7147100	0	6.29584E-06	#DIV/0!	up
sp|Q13557|KCC2D-CAMK2D_HU	sp|Q13557|KCC2D-CAMK2D_HUMAN;sp|	0	6064700	0	5.34236E-06	#DIV/0!	up
sp|Q13561|DCTN2-DCTN2_HUMA	sp|Q13561|DCTN2-DCTN2_HUMAN	0	86858000	0	7.65127E-05	#DIV/0!	up
sp|Q13765|NACA-NACA_HUMAN	sp|Q13765|NACA-NACA_HUMAN;sp|E9PA	0	11324000	0	9.97525E-06	#DIV/0!	up
sp|Q14152|EIF3A-EIF3A_HUMAN	sp|Q14152|EIF3A-EIF3A_HUMAN	0	5640400	0	4.9686E-06	#DIV/0!	up
sp|Q14203|DCTN1-DCTN1_HUMA	sp|Q14203|DCTN1-DCTN1_HUMAN	0	61603000	0	5.42657E-05	#DIV/0!	up
sp|Q14247|SRC8-CTTN_HUMAN	sp|Q14247|SRC8-CTTN_HUMAN	0	168050000	0	0.000148034	#DIV/0!	up
sp|Q14257|RCN2-RCN2_HUMAN	sp|Q14257|RCN2-RCN2_HUMAN	0	32067000	0	2.82476E-05	#DIV/0!	up
sp|Q14258|TRI25-TRIM25_HUMA	sp|Q14258|TRI25-TRIM25_HUMAN	0	540760000	0	0.000476352	#DIV/0!	up
sp|Q14699|RFTN1-RFTN1_HUMA	sp|Q14699|RFTN1-RFTN1_HUMAN	0	8921200	0	7.85864E-06	#DIV/0!	up
sp|Q14847|LASP1-LASP1_HUMA	sp|Q14847|LASP1-LASP1_HUMAN	0	7652600	0	6.74113E-06	#DIV/0!	up
sp|Q15019|SEPT2-SEPTIN2_HUM	sp|Q15019|SEPT2-SEPTIN2_HUMAN	0	14225000	0	1.25307E-05	#DIV/0!	up
sp|Q15027|ACAP1-ACAP1_HUMA	sp|Q15027|ACAP1-ACAP1_HUMAN	0	80516000	0	7.09261E-05	#DIV/0!	up
sp|Q15029|U5S1-EFTUD2_HUMA	sp|Q15029|U5S1-EFTUD2_HUMAN	0	6711300	0	5.91195E-06	#DIV/0!	up
sp|Q15084|PDIA6-PDIA6_HUMAN	sp|Q15084|PDIA6-PDIA6_HUMAN	0	11249000	0	9.90918E-06	#DIV/0!	up
sp|Q15293|RCN1-RCN1_HUMAN	sp|Q15293|RCN1-RCN1_HUMAN	0	16657000	0	1.46731E-05	#DIV/0!	up
sp|Q15393|SF3B3-SF3B3_HUMAN	sp|Q15393|SF3B3-SF3B3_HUMAN	0	948460000	0	0.000835493	#DIV/0!	up
sp|Q15427|SF3B4-SF3B4_HUMAN	sp|Q15427|SF3B4-SF3B4_HUMAN	0	28890000	0	2.5449E-05	#DIV/0!	up
sp|Q15428|SF3A2-SF3A2_HUMAN	sp|Q15428|SF3A2-SF3A2_HUMAN	0	232620000	0	0.000204914	#DIV/0!	up
sp|Q15459|SF3A1-SF3A1_HUMAN	sp|Q15459|SF3A1-SF3A1_HUMAN	0	1898400000	0	0.00167229	#DIV/0!	up
sp|Q15582|BGH3-TGFBI_HUMAN	sp|Q15582|BGH3-TGFBI_HUMAN	0	14549000	0	1.28161E-05	#DIV/0!	up
sp|Q15717|ELAV1-ELAVL1_HUM	sp|Q15717|ELAV1-ELAVL1_HUMAN	0	25276000	0	2.22655E-05	#DIV/0!	up
sp|Q16181|SEPT7-SEPTIN7_HUM	sp|Q16181|SEPT7-SEPTIN7_HUMAN	0	13979000	0	1.2314E-05	#DIV/0!	up
sp|Q16658|FSCN1-FSCN1_HUMA	sp|Q16658|FSCN1-FSCN1_HUMAN	0	4408000	0	3.88298E-06	#DIV/0!	up
sp|Q2VIR3|IF2GL-EIF2S3B_HUM	sp|Q2VIR3|IF2GL-EIF2S3B_HUMAN;sp|P4	0	2724600	0	2.40009E-06	#DIV/0!	up
sp|Q32MZ4|LRRF1-LRRFIP1_HUM	sp|Q32MZ4|LRRF1-LRRFIP1_HUMAN	0	75391000	0	6.64115E-05	#DIV/0!	up
sp|Q32P44|EMAL3-EML3_HUMA	sp|Q32P44|EMAL3-EML3_HUMAN	0	2809600	0	2.47496E-06	#DIV/0!	up
sp|Q3ZCM7|TBB8-TUBB8_HUMA	sp|Q3ZCM7|TBB8-TUBB8_HUMAN	0	26956000	0	2.37454E-05	#DIV/0!	up

sp|Q58FF8|H90B2-HSP90AB2P_H	sp|Q58FF8|H90B2-HSP90AB2P_HUMAN	0	8809700	0	7.76042E-06	#DIV/0!	up
sp|Q5EB52|MEST-MEST_HUMAN	sp|Q5EB52|MEST-MEST_HUMAN	0	13895000	0	1.224E-05	#DIV/0!	up
sp|Q5JSL3|DOC11-DOCK11_HUM	sp|Q5JSL3|DOC11-DOCK11_HUMAN	0	3574900	0	3.14911E-06	#DIV/0!	up
sp|Q5M775|CYTSB-SPECC1_HUM	sp|Q5M775|CYTSB-SPECC1_HUMAN	0	15786000	0	1.39058E-05	#DIV/0!	up
sp|Q5SSJ5|HP1B3-HP1BP3_HUMA	sp|Q5SSJ5|HP1B3-HP1BP3_HUMAN	0	111690000	0	9.83871E-05	#DIV/0!	up
sp|Q5VT25|MRCKA-CDC42BPA_	sp|Q5VT25|MRCKA-CDC42BPA_HUMAN	0	5817200	0	5.12434E-06	#DIV/0!	up
sp|Q63ZY3|KANK2-KANK2_HUM	sp|Q63ZY3|KANK2-KANK2_HUMAN	0	12608000	0	1.11063E-05	#DIV/0!	up
sp|Q6NYC8|PPR18-PPP1R18_HUM	sp|Q6NYC8|PPR18-PPP1R18_HUMAN	0	6217200	0	5.4767E-06	#DIV/0!	up
sp|Q6PKG0|LARP1-LARP1_HUM	sp|Q6PKG0|LARP1-LARP1_HUMAN	0	7478600	0	6.58786E-06	#DIV/0!	up
sp|Q6UWE0|LRSM1-LRSAM1_HU	sp|Q6UWE0|LRSM1-LRSAM1_HUMAN	0	82940000	0	7.30614E-05	#DIV/0!	up
sp|Q6YHK3|CD109-CD109_HUMA	sp|Q6YHK3|CD109-CD109_HUMAN	0	43140000	0	3.80018E-05	#DIV/0!	up
sp|Q6ZXV5|TMTC3-TMTC3_HUM	sp|Q6ZXV5|TMTC3-TMTC3_HUMAN	0	19895000	0	1.75254E-05	#DIV/0!	up
sp|Q702N8|XIRP1-XIRP1_HUMAN	sp|Q702N8|XIRP1-XIRP1_HUMAN	0	6503600	0	5.72899E-06	#DIV/0!	up
sp|Q7L014|DDX46-DDX46_HUMA	sp|Q7L014|DDX46-DDX46_HUMAN	0	96471000	0	8.49808E-05	#DIV/0!	up
sp|Q7RTV0|PHF5A-PHF5A_HUM	sp|Q7RTV0|PHF5A-PHF5A_HUMAN	0	71934000	0	6.33663E-05	#DIV/0!	up
sp|Q7Z2W4|ZCCHV-ZC3HAV1_H	sp|Q7Z2W4|ZCCHV-ZC3HAV1_HUMAN	0	8151700	0	7.18079E-06	#DIV/0!	up
sp|Q7Z478|DHX29-DHX29_HUMA	sp|Q7Z478|DHX29-DHX29_HUMAN	0	17648000	0	1.5546E-05	#DIV/0!	up
sp|Q86UP2|KTN1-KTN1_HUMAN	sp|Q86UP2|KTN1-KTN1_HUMAN	0	18548000	0	1.63388E-05	#DIV/0!	up
sp|Q86V48|LUZP1-LUZP1_HUMA	sp|Q86V48|LUZP1-LUZP1_HUMAN	0	35496000	0	3.12682E-05	#DIV/0!	up
sp|Q86YZ3|HORN-HRNR_HUMA	sp|Q86YZ3|HORN-HRNR_HUMAN;CON__	0	5284900	0	4.65544E-06	#DIV/0!	up
sp|Q8IY17|PLPL6-PNPLA6_HUM	sp|Q8IY17|PLPL6-PNPLA6_HUMAN	0	7571600	0	6.66978E-06	#DIV/0!	up
sp|Q8N556|AFAP1-AFAP1_HUMA	sp|Q8N556|AFAP1-AFAP1_HUMAN	0	4872500	0	4.29216E-06	#DIV/0!	up
sp|Q8N5C1|CAHM5-CALHM5_HU	sp|Q8N5C1|CAHM5-CALHM5_HUMAN	0	16369000	0	1.44194E-05	#DIV/0!	up
sp|Q8N8S7|ENAH-ENAH_HUMA	sp|Q8N8S7|ENAH-ENAH_HUMAN	0	6503800	0	5.72916E-06	#DIV/0!	up
sp|Q8NBN3|TM87A-TMEM87A_H	sp|Q8NBN3|TM87A-TMEM87A_HUMAN	0	34812000	0	3.06657E-05	#DIV/0!	up
sp|Q8NEV1|CSK23-CSNK2A3_HU	sp|Q8NEV1|CSK23-CSNK2A3_HUMAN;sp	0	42273000	0	3.72381E-05	#DIV/0!	up
sp|Q8TDN6|BRX1-BRIX1_HUMA	sp|Q8TDN6|BRX1-BRIX1_HUMAN	0	6935600	0	6.10953E-06	#DIV/0!	up
sp|Q8TES7|FBF1-FBF1_HUMAN	sp|Q8TES7|FBF1-FBF1_HUMAN	0	22377000	0	1.97118E-05	#DIV/0!	up
sp|Q8TF72|SHRM3-SHROOM3_H	sp|Q8TF72|SHRM3-SHROOM3_HUMAN	0	6502000	0	5.72758E-06	#DIV/0!	up
sp|Q8WUJ3|CEMIP-CEMIP_HUM	sp|Q8WUJ3|CEMIP-CEMIP_HUMAN	0	90953000	0	8.012E-05	#DIV/0!	up
sp|Q8WX93|PALLD-PALLD_HUM	sp|Q8WX93|PALLD-PALLD_HUMAN	0	23996000	0	2.11379E-05	#DIV/0!	up
sp|Q8WYL5|SSH1-SSH1_HUMAN	sp|Q8WYL5|SSH1-SSH1_HUMAN	0	2066000	0	1.81993E-06	#DIV/0!	up
sp|Q92522|H1X-H1FX_HUMAN	sp|Q92522|H1X-H1FX_HUMAN	0	16035000	0	1.41251E-05	#DIV/0!	up
sp|Q92614|MY18A-MYO18A_HUM	sp|Q92614|MY18A-MYO18A_HUMAN	0	155190000	0	0.000136706	#DIV/0!	up
sp|Q92626|PXDN-PXDN_HUMAN	sp|Q92626|PXDN-PXDN_HUMAN	0	8285000	0	7.29821E-06	#DIV/0!	up
sp|Q92747|ARC1A-ARPC1A_HUM	sp|Q92747|ARC1A-ARPC1A_HUMAN	0	85039000	0	7.49104E-05	#DIV/0!	up
sp|Q92900|RENT1-UPF1_HUMAN	sp|Q92900|RENT1-UPF1_HUMAN	0	102520000	0	9.03093E-05	#DIV/0!	up
sp|Q92908|GATA6-GATA6_HUM	sp|Q92908|GATA6-GATA6_HUMAN	0	4023500	0	3.54428E-06	#DIV/0!	up
sp|Q92974|ARHG2-ARHGEF2_HU	sp|Q92974|ARHG2-ARHGEF2_HUMAN	0	1716700	0	1.51223E-06	#DIV/0!	up
sp|Q969Q0|RL36L-RPL36AL_HUM	sp|Q969Q0|RL36L-RPL36AL_HUMAN;sp|P	0	18880000	0	1.66313E-05	#DIV/0!	up
sp|Q96AQ6|PBIP1-PBXIP1_HUMA	sp|Q96AQ6|PBIP1-PBXIP1_HUMAN	0	7506600	0	6.61252E-06	#DIV/0!	up
sp|Q96CW1|AP2M1-AP2M1_HUM	sp|Q96CW1|AP2M1-AP2M1_HUMAN	0	38861000	0	3.42324E-05	#DIV/0!	up
sp|Q96ER9|MITOK-CCDC51_HUM	sp|Q96ER9|MITOK-CCDC51_HUMAN	0	51110000	0	4.50225E-05	#DIV/0!	up

sp|Q96HS1|PGAM5-PGAM5_HUM	sp|Q96HS1|PGAM5-PGAM5_HUMAN	0	34459000	0	3.03547E-05	#DIV/0!	up
sp|Q96K37|S35E1-SLC35E1_HUM	sp|Q96K37|S35E1-SLC35E1_HUMAN	0	6163400	0	5.4293E-06	#DIV/0!	up
sp|Q96L58|B3GT6-B3GALT6_HU	sp|Q96L58|B3GT6-B3GALT6_HUMAN	0	2487900	0	2.19158E-06	#DIV/0!	up
sp|Q96LZ7|RMD2-RMDN2_HUMA	sp|Q96LZ7|RMD2-RMDN2_HUMAN	0	12878000	0	1.13442E-05	#DIV/0!	up
sp|Q96N67|DOCK7-DOCK7_HUM	sp|Q96N67|DOCK7-DOCK7_HUMAN	0	28380000	0	2.49998E-05	#DIV/0!	up
sp|Q96SB3|NEB2-PPP1R9B_HUM	sp|Q96SB3|NEB2-PPP1R9B_HUMAN	0	28453000	0	2.50641E-05	#DIV/0!	up
sp|Q99784|NOE1-OLFM1_HUMA	sp|Q99784|NOE1-OLFM1_HUMAN	0	13266000	0	1.16859E-05	#DIV/0!	up
sp|Q99848|EBP2-EBNA1BP2_HU	sp|Q99848|EBP2-EBNA1BP2_HUMAN	0	16433000	0	1.44757E-05	#DIV/0!	up
sp|Q99878|H2A1J-H2AC14_HUMA	sp|Q99878|H2A1J-H2AC14_HUMAN;sp|Q9	0	88343000	0	7.78209E-05	#DIV/0!	up
sp|Q9BPW8|NIPS1-NIPSNAP1_HU	sp|Q9BPW8|NIPS1-NIPSNAP1_HUMAN	0	5474300	0	4.82228E-06	#DIV/0!	up
sp|Q9BQE3|TBA1C-TUBA1C_HU	sp|Q9BQE3|TBA1C-TUBA1C_HUMAN	0	14492000	0	1.27659E-05	#DIV/0!	up
sp|Q9BQG0|MBB1A-MYBBP1A_H	sp|Q9BQG0|MBB1A-MYBBP1A_HUMAN	0	15199000	0	1.33887E-05	#DIV/0!	up
sp|Q9BT78|CSN4-COPS4_HUMAN	sp|Q9BT78|CSN4-COPS4_HUMAN	0	27287000	0	2.4037E-05	#DIV/0!	up
sp|Q9BUF5|TBB6-TUBB6_HUMA	sp|Q9BUF5|TBB6-TUBB6_HUMAN	0	195090000	0	0.000171854	#DIV/0!	up
sp|Q9BUJ2|HNRL1-HNRNPUL1_H	sp|Q9BUJ2|HNRL1-HNRNPUL1_HUMAN	0	14477000	0	1.27527E-05	#DIV/0!	up
sp|Q9BXN1|ASPN-ASPN_HUMAN	sp|Q9BXN1|ASPN-ASPN_HUMAN	0	14083000	0	1.24056E-05	#DIV/0!	up
sp|Q9BZF9|UACA-UACA_HUMA	sp|Q9BZF9|UACA-UACA_HUMAN	0	77701000	0	6.84464E-05	#DIV/0!	up
sp|Q9H2D6|TARA-TRIOBP_HUM	sp|Q9H2D6|TARA-TRIOBP_HUMAN	0	28205000	0	2.48456E-05	#DIV/0!	up
sp|Q9H3U1|UN45A-UNC45A_HU	sp|Q9H3U1|UN45A-UNC45A_HUMAN	0	8087800	0	7.1245E-06	#DIV/0!	up
sp|Q9H4M9|EHD1-EHD1_HUMAN	sp|Q9H4M9|EHD1-EHD1_HUMAN;sp|Q9N	0	2321100	0	2.04464E-06	#DIV/0!	up
sp|Q9H772|GREM2-GREM2_HUM	sp|Q9H772|GREM2-GREM2_HUMAN;sp|O	0	7987700	0	7.03632E-06	#DIV/0!	up
sp|Q9HC84|MUC5B-MUC5B_HUM	sp|Q9HC84|MUC5B-MUC5B_HUMAN	0	36434000	0	3.20945E-05	#DIV/0!	up
sp|Q9HCE1|MOV10-MOV10_HUM	sp|Q9HCE1|MOV10-MOV10_HUMAN	0	17546000	0	1.54562E-05	#DIV/0!	up
sp|Q9HD67|MYO10-MYO10_HUM	sp|Q9HD67|MYO10-MYO10_HUMAN	0	12914000	0	1.13759E-05	#DIV/0!	up
sp|Q9NR12|PDLI7-PDLIM7_HUM	sp|Q9NR12|PDLI7-PDLIM7_HUMAN	0	36085000	0	3.17871E-05	#DIV/0!	up
sp|Q9NR30|DDX21-DDX21_HUM	sp|Q9NR30|DDX21-DDX21_HUMAN	0	2288300	0	2.01575E-06	#DIV/0!	up
sp|Q9NS87|KIF15-KIF15_HUMAN	sp|Q9NS87|KIF15-KIF15_HUMAN	0	6563300	0	5.78157E-06	#DIV/0!	up
sp|Q9NUQ6|SPS2L-SPATS2L_HU	sp|Q9NUQ6|SPS2L-SPATS2L_HUMAN	0	6971600	0	6.14124E-06	#DIV/0!	up
sp|Q9NVA2|SEP11-SEPTIN11_HU	sp|Q9NVA2|SEP11-SEPTIN11_HUMAN	0	24233000	0	2.13467E-05	#DIV/0!	up
sp|Q9NZ01|TECR-TECR_HUMAN	sp|Q9NZ01|TECR-TECR_HUMAN	0	27230000	0	2.39868E-05	#DIV/0!	up
sp|Q9NZ32|ARP10-ACTR10_HUM	sp|Q9NZ32|ARP10-ACTR10_HUMAN	0	8783700	0	7.73751E-06	#DIV/0!	up
sp|Q9NZB2|F120A-FAM120A_HU	sp|Q9NZB2|F120A-FAM120A_HUMAN	0	31197000	0	2.74813E-05	#DIV/0!	up
sp|Q9NZI8|IF2B1-IGF2BP1_HUMA	sp|Q9NZI8|IF2B1-IGF2BP1_HUMAN	0	26877000	0	2.36758E-05	#DIV/0!	up
sp|Q9P0V3|SH3B4-SH3BP4_HUM	sp|Q9P0V3|SH3B4-SH3BP4_HUMAN	0	12945000	0	1.14032E-05	#DIV/0!	up
sp|Q9P0X4|CAC1I-CACNA1I_HU	sp|Q9P0X4|CAC1I-CACNA1I_HUMAN	0	37438000	0	3.29789E-05	#DIV/0!	up
sp|Q9P121|NTRI-NTM_HUMAN	sp|Q9P121|NTRI-NTM_HUMAN	0	17198000	0	1.51496E-05	#DIV/0!	up
sp|Q9P266|JCAD-JCAD_HUMAN	sp|Q9P266|JCAD-JCAD_HUMAN	0	3604700	0	3.17536E-06	#DIV/0!	up
sp|Q9P2E9|RRBP1-RRBP1_HUMA	sp|Q9P2E9|RRBP1-RRBP1_HUMAN	0	16582000	0	1.4607E-05	#DIV/0!	up
sp|Q9UBI6|GBG12-GNG12_HUMA	sp|Q9UBI6|GBG12-GNG12_HUMAN	0	7944900	0	6.99862E-06	#DIV/0!	up
sp|Q9UBM7|DHCR7-DHCR7_HUN	sp|Q9UBM7|DHCR7-DHCR7_HUMAN	0	4514100	0	3.97645E-06	#DIV/0!	up
sp|Q9UEY8|ADDG-ADD3_HUMA	sp|Q9UEY8|ADDG-ADD3_HUMAN	0	7108900	0	6.26219E-06	#DIV/0!	up
sp|Q9UGR2|Z3H7B-ZC3H7B_HUN	sp|Q9UGR2|Z3H7B-ZC3H7B_HUMAN	0	3851100	0	3.39241E-06	#DIV/0!	up
sp|Q9UHB9|SRP68-SRP68_HUMA	sp|Q9UHB9|SRP68-SRP68_HUMAN	0	6227500	0	5.48577E-06	#DIV/0!	up

sp|Q9UJW0|DCTN4-DCTN4_HUM	sp|Q9UJW0|DCTN4-DCTN4_HUMAN	0	64617000	0	5.69208E-05	#DIV/0!	up
sp|Q9UN86|G3BP2-G3BP2_HUMA	sp|Q9UN86|G3BP2-G3BP2_HUMAN	0	19621000	0	1.7284E-05	#DIV/0!	up
sp|Q9UNX3|RL26L-RPL26L1_HU	sp|Q9UNX3|RL26L-RPL26L1_HUMAN	0	9973400	0	8.78551E-06	#DIV/0!	up
sp|Q9UPN3|MACF1-MACF1_HUM	sp|Q9UPN3|MACF1-MACF1_HUMAN	0	6248000	0	5.50383E-06	#DIV/0!	up
sp|Q9UQ03|COR2B-CORO2B_HU	sp|Q9UQ03|COR2B-CORO2B_HUMAN	0	210610000	0	0.000185525	#DIV/0!	up
sp|Q9UQE7|SMC3-SMC3_HUMAN	sp|Q9UQE7|SMC3-SMC3_HUMAN	0	4850300	0	4.2726E-06	#DIV/0!	up
sp|Q9Y230|RUVB2-RUVBL2_HU	sp|Q9Y230|RUVB2-RUVBL2_HUMAN	0	9447900	0	8.3226E-06	#DIV/0!	up
sp|Q9Y265|RUVB1-RUVBL1_HU	sp|Q9Y265|RUVB1-RUVBL1_HUMAN	0	26687000	0	2.35084E-05	#DIV/0!	up
sp|Q9Y2D5|AKAP2-AKAP2_HUM	sp|Q9Y2D5|AKAP2-AKAP2_HUMAN	0	10243000	0	9.023E-06	#DIV/0!	up
sp|Q9Y2X3|NOP58-NOP58_HUMA	sp|Q9Y2X3|NOP58-NOP58_HUMAN	0	9579200	0	8.43826E-06	#DIV/0!	up
sp|Q9Y3B4|SF3B6-SF3B6_HUMA	sp|Q9Y3B4|SF3B6-SF3B6_HUMAN	0	66837000	0	5.88763E-05	#DIV/0!	up
sp|Q9Y3I0|RTCB-RTCB_HUMAN	sp|Q9Y3I0|RTCB-RTCB_HUMAN	0	23887000	0	2.10419E-05	#DIV/0!	up
sp|Q9Y490|TLN1-TLN1_HUMAN	sp|Q9Y490|TLN1-TLN1_HUMAN	0	40795000	0	3.59361E-05	#DIV/0!	up
sp|Q9Y4F1|FARP1-FARP1_HUMA	sp|Q9Y4F1|FARP1-FARP1_HUMAN	0	26662000	0	2.34864E-05	#DIV/0!	up
sp|Q9Y4P3|TBL2-TBL2_HUMAN	sp|Q9Y4P3|TBL2-TBL2_HUMAN	0	24578000	0	2.16506E-05	#DIV/0!	up
sp|Q9Y678|COPG1-COPG1_HUM	sp|Q9Y678|COPG1-COPG1_HUMAN	0	13395000	0	1.17996E-05	#DIV/0!	up
sp|Q9Y6C2|EMIL1-EMILIN1_HU	sp|Q9Y6C2|EMIL1-EMILIN1_HUMAN	0	8640200	0	7.6111E-06	#DIV/0!	up
sp|Q9Y6M1|IF2B2-IGF2BP2_HUM	sp|Q9Y6M1|IF2B2-IGF2BP2_HUMAN	0	128260000	0	0.000112984	#DIV/0!	up
sp|O00159|MYO1C-MYO1C_HUM	sp|O00159|MYO1C-MYO1C_HUMAN	1429400000	21272000000	0.008907742	0.018738387	2.10360676	up
sp|O15143|ARC1B-ARPC1B_HUM	sp|O15143|ARC1B-ARPC1B_HUMAN	8103400	139520000	5.04988E-05	0.000122902	2.433768141	up
sp|O15144|ARPC2-ARPC2_HUMA	sp|O15144|ARPC2-ARPC2_HUMAN	1396200	272500000	8.70085E-06	0.000240044	27.58855055	up
sp|O15145|ARPC3-ARPC3_HUMA	sp|O15145|ARPC3-ARPC3_HUMAN	2673400	197940000	1.66601E-05	0.000174364	10.46597356	up
sp|O43390|HNRPR-HNRNPR_HU	sp|O43390|HNRPR-HNRNPR_HUMAN	1813500	65950000	1.13014E-05	5.8095E-05	5.140522343	up
sp|O43707|ACTN4-ACTN4_HUMA	sp|O43707|ACTN4-ACTN4_HUMAN	26508000	759340000	0.000165193	0.000668898	4.049200923	up
sp|O43795|MYO1B-MYO1B_HUM	sp|O43795|MYO1B-MYO1B_HUMAN	574480000	7527300000	0.003580047	0.006630757	1.852142181	up
sp|O43852|CALU-CALU_HUMAN	sp|O43852|CALU-CALU_HUMAN	3039300	57733000	1.89403E-05	5.08567E-05	2.685100631	up
sp|O43854|EDIL3-EDIL3_HUMAN	sp|O43854|EDIL3-EDIL3_HUMAN	25807000	479270000	0.000160824	0.000422186	2.625141602	up
sp|O60716|CTND1-CTNND1_HUM	sp|O60716|CTND1-CTNND1_HUMAN	678520	8075100	4.2284E-06	7.11331E-06	1.682268322	up
sp|O75165|DJC13-DNAJC13_HUM	sp|O75165|DJC13-DNAJC13_HUMAN	3510500	584430000	2.18768E-05	0.000514821	23.53279626	up
sp|O75369|FLNB-FLNB_HUMAN	sp|O75369|FLNB-FLNB_HUMAN	7072700	192720000	4.40757E-05	0.000169766	3.851692084	up
sp|O95425|SVIL-SVIL_HUMAN	sp|O95425|SVIL-SVIL_HUMAN	29173000	1278900000	0.0001818	0.001126576	6.196771847	up
sp|P01857|IGHG1-IGHG1_HUMAN	sp|P01857|IGHG1-IGHG1_HUMAN;sp|P0D	123650000	11915000000	0.000770563	0.010495857	13.62102961	up
sp|P02452|CO1A1-COL1A1_HUM	sp|P02452|CO1A1-COL1A1_HUMAN	11838000	374470000	7.37721E-05	0.000329869	4.471453371	up
sp|P02751|FINC-FN1_HUMAN	sp|P02751|FINC-FN1_HUMAN	177430000	3357800000	0.001105709	0.002957867	2.675086215	up
sp|P04216|THY1-THY1_HUMAN	sp|P04216|THY1-THY1_HUMAN	4511700	135850000	2.8116E-05	0.00011967	4.256272645	up
sp|P04899|GNAI2-GNAI2_HUMAN	sp|P04899|GNAI2-GNAI2_HUMAN	21184000	258760000	0.000132015	0.00022794	1.726629616	up
sp|P05388|RLA0-RPLP0_HUMAN	sp|P05388|RLA0-RPLP0_HUMAN;sp|Q8NH	20147000	1018400000	0.000125552	0.000897103	7.145259654	up
sp|P06396|GELS-GSN_HUMAN;C	sp|P06396|GELS-GSN_HUMAN;CON__Q3	227770000	3983100000	0.001419418	0.003508691	2.471921639	up
sp|P06753|TPM3-TPM3_HUMAN	sp|P06753|TPM3-TPM3_HUMAN	37880000	1067000000	0.000236061	0.000939914	3.981662641	up
sp|P07237|PDIA1-P4HB_HUMAN	sp|P07237|PDIA1-P4HB_HUMAN	3707600	152090000	2.3105E-05	0.000133975	5.79852852	up
sp|P07437|TBB5-TUBB_HUMAN	sp|P07437|TBB5-TUBB_HUMAN	135930000	1574600000	0.000847089	0.001387056	1.637438289	up
sp|P07814|SYEP-EPRS_HUMAN	sp|P07814|SYEP-EPRS_HUMAN	628650	19554000	3.91762E-06	1.7225E-05	4.39679991	up
sp|P07900|HS90A-HSP90AA1_HU	sp|P07900|HS90A-HSP90AA1_HUMAN	1323800	89261000	8.24966E-06	7.86295E-05	9.531239496	up

sp|P07951|TPM2-TPM2_HUMAN	sp|P07951|TPM2-TPM2_HUMAN	13474000	196750000	8.39673E-05	0.000173316	2.064088001	up
sp|P08123|CO1A2-COL1A2_HUM	sp|P08123|CO1A2-COL1A2_HUMAN	4420000	231890000	2.75446E-05	0.000204271	7.416000705	up
sp|P08238|HS90B-HSP90AB1_HU	sp|P08238|HS90B-HSP90AB1_HUMAN	37952000	421260000	0.000236509	0.000371086	1.569009479	up
sp|P08670|VIME-VIM_HUMAN;sp	sp|P08670|VIME-VIM_HUMAN	666050000	7528200000	0.004150694	0.00663155	1.597696659	up
sp|P09493|TPM1-TPM1_HUMAN	sp|P09493|TPM1-TPM1_HUMAN	196170000	3305300000	0.001222493	0.00291162	2.381706834	up
sp|P0DP25|CALM3-CALM3_HUM	sp|P0DP25|CALM3-CALM3_HUMAN;sp|P(	6230000	79035000	3.88241E-05	6.96215E-05	1.793252406	up
sp|P11021|BIP-HSPA5_HUMAN	sp|P11021|BIP-HSPA5_HUMAN	61087000	922370000	0.000380682	0.000812511	2.134353659	up
sp|P11142|HSP7C-HSPA8_HUMA	sp|P11142|HSP7C-HSPA8_HUMAN	45606000	560950000	0.000284208	0.000494138	1.738650063	up
sp|P11940|PABP1-PABPC1_HUM	sp|P11940|PABP1-PABPC1_HUMAN;sp|Q9	11366000	686850000	7.08307E-05	0.000605042	8.542092344	up
sp|P12109|CO6A1-COL6A1_HUM	sp|P12109|CO6A1-COL6A1_HUMAN	2358100	100730000	1.46952E-05	8.87325E-05	6.038187946	up
sp|P12111|CO6A3-COL6A3_HUM	sp|P12111|CO6A3-COL6A3_HUMAN	17876000	677950000	0.0001114	0.000597202	5.36089536	up
sp|P12236|ADT3-SLC25A6_HUM	sp|P12236|ADT3-SLC25A6_HUMAN;sp|P1	14789000	180290000	9.21622E-05	0.000158816	1.72322839	up
sp|P12268|IMDH2-IMPDH2_HUM	sp|P12268|IMDH2-IMPDH2_HUMAN	1974700	55433000	1.23059E-05	4.88306E-05	3.968051207	up
sp|P12814|ACTN1-ACTN1_HUM	sp|P12814|ACTN1-ACTN1_HUMAN	113090000	1958300000	0.000704755	0.001725056	2.447738569	up
sp|P12956|XRCC6-XRCC6_HUM	sp|P12956|XRCC6-XRCC6_HUMAN	3510900	223150000	2.18792E-05	0.000196572	8.984386831	up
sp|P13639|EF2-EEF2_HUMAN	sp|P13639|EF2-EEF2_HUMAN	800670	75436000	4.98962E-06	6.64512E-05	13.31788028	up
sp|P14618|KPYM-PKM_HUMAN	sp|P14618|KPYM-PKM_HUMAN	12544000	179610000	7.81718E-05	0.000158217	2.0239719	up
sp|P14625|ENPL-HSP90B1_HUM	sp|P14625|ENPL-HSP90B1_HUMAN	6127700	101070000	3.81866E-05	8.9032E-05	2.331496576	up
sp|P16401|H15-H1-5_HUMAN	sp|P16401|H15-H1-5_HUMAN	6365900	141510000	3.9671E-05	0.000124655	3.142225365	up
sp|P18124|RL7-RPL7_HUMAN	sp|P18124|RL7-RPL7_HUMAN	51773000	623030000	0.000322639	0.000548824	1.70104436	up
sp|P18621|RL17-RPL17_HUMAN	sp|P18621|RL17-RPL17_HUMAN	5066100	264600000	3.15709E-05	0.000233085	7.382884818	up
sp|P19338|NUCL-NCL_HUMAN	sp|P19338|NUCL-NCL_HUMAN	13647000	411120000	8.50454E-05	0.000362153	4.258350691	up
sp|P21333|FLNA-FLNA_HUMAN	sp|P21333|FLNA-FLNA_HUMAN	230010000	3681800000	0.001433377	0.003243277	2.262681816	up
sp|P21589|5NTD-NT5E_HUMAN	sp|P21589|5NTD-NT5E_HUMAN	8595600	316550000	5.35661E-05	0.000278847	5.205663985	up
sp|P23396|RS3-RPS3_HUMAN	sp|P23396|RS3-RPS3_HUMAN	11843000	273370000	7.38033E-05	0.00024081	3.26286522	up
sp|P25398|RS12-RPS12_HUMAN	sp|P25398|RS12-RPS12_HUMAN	5367300	103340000	3.3448E-05	9.10316E-05	2.721589357	up
sp|P26373|RL13-RPL13_HUMAN	sp|P26373|RL13-RPL13_HUMAN	20768000	264390000	0.000129422	0.0002329	1.799535261	up
sp|P27635|RL10-RPL10_HUMAN;	sp|P27635|RL10-RPL10_HUMAN	2573100	308530000	1.60351E-05	0.000271782	16.94926015	up
sp|P30050|RL12-RPL12_HUMAN	sp|P30050|RL12-RPL12_HUMAN	8396000	488210000	5.23222E-05	0.000430061	8.219478279	up
sp|P35268|RL22-RPL22_HUMAN;	sp|P35268|RL22-RPL22_HUMAN	3378600	70019000	2.10548E-05	6.16793E-05	2.929470557	up
sp|P35580|MYH10-MYH10_HUM	sp|P35580|MYH10-MYH10_HUMAN	1620600000	27805000000	0.010099263	0.02449327	2.425253143	up
sp|P36542|ATPG-ATP5F1C_HUM	sp|P36542|ATPG-ATP5F1C_HUMAN	4731700	51336000	2.9487E-05	4.52216E-05	1.533609661	up
sp|P36578|RL4-RPL4_HUMAN	sp|P36578|RL4-RPL4_HUMAN	46116000	995010000	0.000287386	0.000876499	3.049901589	up
sp|P39019|RS19-RPS19_HUMAN	sp|P39019|RS19-RPS19_HUMAN	2060400	84014000	1.284E-05	7.40075E-05	5.763816321	up
sp|P39023|RL3-RPL3_HUMAN;sp|	sp|P39023|RL3-RPL3_HUMAN	29976000	645770000	0.000186805	0.000568855	3.045188609	up
sp|P40429|RL13A-RPL13A_HUM	sp|P40429|RL13A-RPL13A_HUMAN	15969000	229420000	9.95157E-05	0.000202095	2.030783218	up
sp|P42766|RL35-RPL35_HUMAN	sp|P42766|RL35-RPL35_HUMAN	2526000	99438000	1.57415E-05	8.75944E-05	5.564537144	up
sp|P46776|RL27A-RPL27A_HUM	sp|P46776|RL27A-RPL27A_HUMAN	8275400	88252000	5.15707E-05	7.77407E-05	1.50745922	up
sp|P46778|RL21-RPL21_HUMAN	sp|P46778|RL21-RPL21_HUMAN	9588700	140870000	5.97549E-05	0.000124092	2.076676243	up
sp|P46779|RL28-RPL28_HUMAN	sp|P46779|RL28-RPL28_HUMAN	4188600	166930000	2.61025E-05	0.000147048	5.633463907	up
sp|P46781|RS9-RPS9_HUMAN	sp|P46781|RS9-RPS9_HUMAN	6762000	105750000	4.21395E-05	9.31546E-05	2.210625731	up
sp|P46782|RS5-RPS5_HUMAN	sp|P46782|RS5-RPS5_HUMAN	4326700	64486000	2.69632E-05	5.68054E-05	2.106777476	up
sp|P46940|IQGA1-IQGAP1_HUM	sp|P46940|IQGA1-IQGAP1_HUMAN	3572000	161190000	2.226E-05	0.000141991	6.378765788	up

sp|P47755|CAZA2-CAPZA2_HUM	sp|P47755|CAZA2-CAPZA2_HUMAN	29448000	600210000	0.000183514	0.000528722	2.881094037	up
sp|P47756|CAPZB-CAPZB_HUMA	sp|P47756|CAPZB-CAPZB_HUMAN	45350000	1305400000	0.000282612	0.00114992	4.068893551	up
sp|P49327|FAS-FASN_HUMAN	sp|P49327|FAS-FASN_HUMAN	3610700	225340000	2.25012E-05	0.000198501	8.821793654	up
sp|P49411|EFTU-TUFM_HUMAN	sp|P49411|EFTU-TUFM_HUMAN	5697700	130560000	3.5507E-05	0.00011501	3.239071792	up
sp|P50454|SERPH-SERPINH1_HU	sp|P50454|SERPH-SERPINH1_HUMAN	13768000	157020000	8.57995E-05	0.000138318	1.612107977	up
sp|P50914|RL14-RPL14_HUMAN	sp|P50914|RL14-RPL14_HUMAN	23619000	361710000	0.000147189	0.000318628	2.164756342	up
sp|P52272|HNRPM-HNRNPM_HU	sp|P52272|HNRPM-HNRNPM_HUMAN	15255000	236630000	9.50662E-05	0.000208446	2.192641365	up
sp|P52907|CAZA1-CAPZA1_HUM	sp|P52907|CAZA1-CAPZA1_HUMAN	76335000	1629200000	0.000475705	0.001435153	3.016898525	up
sp|P53621|COPA-COPA_HUMAN	sp|P53621|COPA-COPA_HUMAN	2351100	410960000	1.46516E-05	0.000362012	24.70804948	up
sp|P55072|TERA-VCP_HUMAN	sp|P55072|TERA-VCP_HUMAN	734700	27836000	4.57851E-06	2.45206E-05	5.355583553	up
sp|P55084|ECHB-HADHB_HUMA	sp|P55084|ECHB-HADHB_HUMAN	3030200	63175000	1.88836E-05	5.56505E-05	2.947026003	up
sp|P59998|ARPC4-ARPC4_HUMA	sp|P59998|ARPC4-ARPC4_HUMAN	15624000	202010000	9.73657E-05	0.000177949	1.827639979	up
sp|P60866|RS20-RPS20_HUMAN	sp|P60866|RS20-RPS20_HUMAN	9324100	126150000	5.8106E-05	0.000111125	1.912451118	up
sp|P61158|ARP3-ACTR3_HUMAN	sp|P61158|ARP3-ACTR3_HUMAN	18827000	738940000	0.000117326	0.000650928	5.548020598	up
sp|P61160|ARP2-ACTR2_HUMAN	sp|P61160|ARP2-ACTR2_HUMAN	17367000	456840000	0.000108228	0.000402428	3.71834198	up
sp|P61163|ACTZ-ACTR1A_HUMA	sp|P61163|ACTZ-ACTR1A_HUMAN	3190400	80806000	1.9882E-05	7.11816E-05	3.580209855	up
sp|P61247|RS3A-RPS3A_HUMAN	sp|P61247|RS3A-RPS3A_HUMAN	9210100	293150000	5.73955E-05	0.000258234	4.499202746	up
sp|P61254|RL26-RPL26_HUMAN	sp|P61254|RL26-RPL26_HUMAN	5239100	249660000	3.2649E-05	0.000219924	6.736003271	up
sp|P61313|RL15-RPL15_HUMAN	sp|P61313|RL15-RPL15_HUMAN	3386200	290430000	2.11021E-05	0.000255838	12.12380327	up
sp|P61353|RL27-RPL27_HUMAN	sp|P61353|RL27-RPL27_HUMAN	18836000	468290000	0.000117382	0.000412514	3.514278805	up
sp|P62081|RS7-RPS7_HUMAN	sp|P62081|RS7-RPS7_HUMAN	10155000	157390000	6.3284E-05	0.000138644	2.190822633	up
sp|P62241|RS8-RPS8_HUMAN	sp|P62241|RS8-RPS8_HUMAN	10111000	189120000	6.30098E-05	0.000166595	2.643950743	up
sp|P62244|RS15A-RPS15A_HUMA	sp|P62244|RS15A-RPS15A_HUMAN	1306700	210200000	8.1431E-06	0.000185164	22.73876282	up
sp|P62249|RS16-RPS16_HUMAN	sp|P62249|RS16-RPS16_HUMAN	10123000	174830000	6.30846E-05	0.000154007	2.441275158	up
sp|P62263|RS14-RPS14_HUMAN	sp|P62263|RS14-RPS14_HUMAN	16340000	180930000	0.000101828	0.00015938	1.565195325	up
sp|P62269|RS18-RPS18_HUMAN	sp|P62269|RS18-RPS18_HUMAN	6944500	190560000	4.32768E-05	0.000167863	3.878830243	up
sp|P62277|RS13-RPS13_HUMAN	sp|P62277|RS13-RPS13_HUMAN	15976000	433470000	9.95593E-05	0.000381841	3.835314701	up
sp|P62280|RS11-RPS11_HUMAN	sp|P62280|RS11-RPS11_HUMAN	7921200	240930000	4.93634E-05	0.000212234	4.299420402	up
sp|P62424|RL7A-RPL7A_HUMAN	sp|P62424|RL7A-RPL7A_HUMAN	12201000	621500000	7.60343E-05	0.000547476	7.200384976	up
sp|P62701|RS4X-RPS4X_HUMAN	sp|P62701|RS4X-RPS4X_HUMAN	18689000	359110000	0.000116466	0.000316338	2.716135589	up
sp|P62753|RS6-RPS6_HUMAN	sp|P62753|RS6-RPS6_HUMAN	4186200	144280000	2.60876E-05	0.000127095	4.871875388	up
sp|P62829|RL23-RPL23_HUMAN	sp|P62829|RL23-RPL23_HUMAN	3817900	197390000	2.37924E-05	0.00017388	7.308203118	up
sp|P62847|RS24-RPS24_HUMAN	sp|P62847|RS24-RPS24_HUMAN	5488300	96219000	3.4202E-05	8.47588E-05	2.478180912	up
sp|P62851|RS25-RPS25_HUMAN	sp|P62851|RS25-RPS25_HUMAN	2391000	76315000	1.49002E-05	6.72255E-05	4.511701346	up
sp|P62854|RS26-RPS26_HUMAN;s	sp|P62854|RS26-RPS26_HUMAN;sp|Q5JNZ	4212400	129010000	2.62509E-05	0.000113644	4.329161448	up
sp|P62888|RL30-RPL30_HUMAN	sp|P62888|RL30-RPL30_HUMAN	12453000	264040000	7.76047E-05	0.000232591	2.997131148	up
sp|P62899|RL31-RPL31_HUMAN	sp|P62899|RL31-RPL31_HUMAN	15193000	171050000	9.46798E-05	0.000150677	1.59143737	up
sp|P62906|RL10A-RPL10A_HUMA	sp|P62906|RL10A-RPL10A_HUMAN	63085000	743630000	0.000393133	0.00065506	1.666252469	up
sp|P62910|RL32-RPL32_HUMAN	sp|P62910|RL32-RPL32_HUMAN	3885000	104100000	2.42106E-05	9.17011E-05	3.787648924	up
sp|P62913|RL11-RPL11_HUMAN	sp|P62913|RL11-RPL11_HUMAN	20333000	393780000	0.000126711	0.000346879	2.737550915	up
sp|P62917|RL8-RPL8_HUMAN	sp|P62917|RL8-RPL8_HUMAN	20587000	416380000	0.000128294	0.000366787	2.858951629	up
sp|P62979|RS27A-RPS27A_HUMA	sp|P62979|RS27A-RPS27A_HUMAN;sp|P62	33296000	357960000	0.000207494	0.000315325	1.519681049	up
sp|P63244|RACK1-RACK1_HUMA	sp|P63244|RACK1-RACK1_HUMAN	3849100	208500000	2.39868E-05	0.000183666	7.656968778	up

sp|P63261|ACTG-ACTG1_HUMAI	sp|P63261|ACTG-ACTG1_HUMAN	781530000	8293800000	0.004870343	0.007305962	1.500092073	up
sp|P63267|ACTH-ACTG2_HUMAI	sp|P63267|ACTH-ACTG2_HUMAN	3432700	60364000	2.13919E-05	5.31743E-05	2.485719799	up
sp|P67936|TPM4-TPM4_HUMAN	sp|P67936|TPM4-TPM4_HUMAN	71628000	1620100000	0.000446372	0.001427137	3.19719414	up
sp|P68363|TBA1B-TUBA1B_HUM	sp|P68363|TBA1B-TUBA1B_HUMAN;sp|P(	1885700	65062000	1.17513E-05	5.73128E-05	4.877135475	up
sp|P68371|TBB4B-TUBB4B_HUM	sp|P68371|TBB4B-TUBB4B_HUMAN	301620000	4225500000	0.001879637	0.003722219	1.980286294	up
sp|P78527|PRKDC-PRKDC_HUM/	sp|P78527|PRKDC-PRKDC_HUMAN	2741600	280380000	1.70851E-05	0.000246985	14.45616016	up
sp|P83731|RL24-RPL24_HUMAN	sp|P83731|RL24-RPL24_HUMAN	11189000	156650000	6.97277E-05	0.000137992	1.979015231	up
sp|P84098|RL19-RPL19_HUMAN	sp|P84098|RL19-RPL19_HUMAN	5664100	95013000	3.52976E-05	8.36964E-05	2.371166933	up
sp|P84103|SRSF3-SRSF3_HUMAN	sp|P84103|SRSF3-SRSF3_HUMAN	2953400	65674000	1.8405E-05	5.78519E-05	3.143266538	up
sp|Q00325|MPCP-SLC25A3_HUM	sp|Q00325|MPCP-SLC25A3_HUMAN	4814100	64545000	3.00005E-05	5.68573E-05	1.895210741	up
sp|Q00610|CLH1-CLTC_HUMAN;	sp|Q00610|CLH1-CLTC_HUMAN	54832000	1172200000	0.000341702	0.001032584	3.02188297	up
sp|Q00839|HNRPU-HNRNPU_HU	sp|Q00839|HNRPU-HNRNPU_HUMAN	4917300	140830000	3.06437E-05	0.000124056	4.048354037	up
sp|Q01082|SPTB2-SPTBN1_HUM/	sp|Q01082|SPTB2-SPTBN1_HUMAN	94468000	3033800000	0.000588706	0.002672458	4.539544077	up
sp|Q02543|RL18A-RPL18A_HUM.	sp|Q02543|RL18A-RPL18A_HUMAN	4509700	179080000	2.81036E-05	0.000157751	5.613186147	up
sp|Q02878|RL6-RPL6_HUMAN	sp|Q02878|RL6-RPL6_HUMAN	34434000	426240000	0.000214586	0.000375472	1.749752969	up
sp|Q03135|CAV1-CAV1_HUMAN.	sp|Q03135|CAV1-CAV1_HUMAN	8870100	349090000	5.52767E-05	0.000307511	5.563126089	up
sp|Q05682|CALD1-CALD1_HUM/	sp|Q05682|CALD1-CALD1_HUMAN	56980000	1785200000	0.000355088	0.001572573	4.428681464	up
sp|Q07065|CKAP4-CKAP4_HUM/	sp|Q07065|CKAP4-CKAP4_HUMAN	124860000	2657700000	0.000778103	0.002341153	3.008795265	up
sp|Q07157|ZO1-TJP1_HUMAN	sp|Q07157|ZO1-TJP1_HUMAN	552500	147640000	3.44307E-06	0.000130055	37.77302494	up
sp|Q08211|DHX9-DHX9_HUMAN	sp|Q08211|DHX9-DHX9_HUMAN	2036700	101520000	1.26923E-05	8.94284E-05	7.045868879	up
sp|Q08431|MFGM-MFGE8_HUM/	sp|Q08431|MFGM-MFGE8_HUMAN	34796000	888200000	0.000216842	0.00078241	3.608207109	up
sp|Q0ZGT2|NEXN-NEXN_HUMA'	sp|Q0ZGT2|NEXN-NEXN_HUMAN	229340000	4834900000	0.001429202	0.004259037	2.980009846	up
sp|Q12905|ILF2-ILF2_HUMAN	sp|Q12905|ILF2-ILF2_HUMAN	1989900	72820000	1.24007E-05	6.41467E-05	5.172844624	up
sp|Q12965|MYO1E-MYO1E_HUM	sp|Q12965|MYO1E-MYO1E_HUMAN	4593200	320020000	2.86239E-05	0.000281904	9.848538249	up
sp|Q13009|TIAM1-TIAM1_HUMA	sp|Q13009|TIAM1-TIAM1_HUMAN	1636600	18124000	1.0199E-05	1.59653E-05	1.565386261	up
sp|Q13045|FLII-FLII_HUMAN	sp|Q13045|FLII-FLII_HUMAN	5330300	479800000	3.32174E-05	0.000422653	12.7238518	up
sp|Q13509|TBB3-TUBB3_HUMAN	sp|Q13509|TBB3-TUBB3_HUMAN	10496000	230300000	6.5409E-05	0.00020287	3.101559572	up
sp|Q13813|SPTN1-SPTAN1_HUM.	sp|Q13813|SPTN1-SPTAN1_HUMAN	83626000	3335100000	0.000521141	0.002937871	5.637383078	up
sp|Q13885|TBB2A-TUBB2A_HUN	sp|Q13885|TBB2A-TUBB2A_HUMAN;sp|Q	2907100	83509000	1.81165E-05	7.35626E-05	4.060535607	up
sp|Q14204|DYHC1-DYNC1H1_HU	sp|Q14204|DYHC1-DYNC1H1_HUMAN	897110	278590000	5.59061E-06	0.000245408	43.89649422	up
sp|Q14315|FLNC-FLNC_HUMAN	sp|Q14315|FLNC-FLNC_HUMAN	15670000	514270000	9.76524E-05	0.000453018	4.639083667	up
sp|Q14764|MVP-MVP_HUMAN	sp|Q14764|MVP-MVP_HUMAN	2962400	320810000	1.84611E-05	0.0002826	15.3078504	up
sp|Q15149|PLEC-PLEC_HUMAN	sp|Q15149|PLEC-PLEC_HUMAN	72369000	2688400000	0.00045099	0.002368197	5.251112544	up
sp|Q15746|MYLK-MYLK_HUMAI	sp|Q15746|MYLK-MYLK_HUMAN	268110000	5494500000	0.001670809	0.004840075	2.896844453	up
sp|Q16643|DREB-DBN1_HUMAN	sp|Q16643|DREB-DBN1_HUMAN	949690000	11478000000	0.005918283	0.010110906	1.708418897	up
sp|Q562R1|ACTBL-ACTBL2_HUN	sp|Q562R1|ACTBL-ACTBL2_HUMAN	3079500	44763000	1.91908E-05	3.94315E-05	2.054702682	up
sp|Q69YQ0|CYTSA-SPECC1L_HU	sp|Q69YQ0|CYTSA-SPECC1L_HUMAN	2553400	308360000	1.59123E-05	0.000271633	17.07061604	up
sp|Q6NZI2|CAVN1-CAVIN1_HUN	sp|Q6NZI2|CAVN1-CAVIN1_HUMAN	35060000	760560000	0.000218487	0.000669973	3.066419587	up
sp|Q6WCQ1|MPRIP-MPRIP_HUM	sp|Q6WCQ1|MPRIP-MPRIP_HUMAN	73593000	2569900000	0.000458617	0.002263811	4.936165683	up
sp|Q71U36|TBA1A-TUBA1A_HUI	sp|Q71U36|TBA1A-TUBA1A_HUMAN;sp|I	415330000	4643000000	0.002588256	0.004089993	1.580212136	up
sp|Q71UM5|RS27L-RPS27L_HUM	sp|Q71UM5|RS27L-RPS27L_HUMAN	6981500	96264000	4.35073E-05	8.47984E-05	1.949059838	up
sp|Q8NHM4|TRY6-PRSS3P2_HUN	sp|Q8NHM4|TRY6-PRSS3P2_HUMAN;sp|P	3379800	40993000	2.10623E-05	3.61105E-05	1.714465349	up
sp|Q8WWI1|LMO7-LMO7_HUMA	sp|Q8WWI1|LMO7-LMO7_HUMAN	2119300	400730000	1.32071E-05	0.000353001	26.72818259	up

sp|Q969G5|CAVN3-CAVIN3_HUN	sp|Q969G5|CAVN3-CAVIN3_HUMAN	5834200	76167000	3.63576E-05	6.70951E-05	1.845421383	up
sp|Q99715|COCA1-COL12A1_HU	sp|Q99715|COCA1-COL12A1_HUMAN	2205400	111260000	1.37436E-05	9.80083E-05	7.13118489	up
sp|Q9NVI7|ATD3A-ATAD3A_HU	sp|Q9NVI7|ATD3A-ATAD3A_HUMAN	6072900	208130000	3.78451E-05	0.000183341	4.844495598	up
sp|Q9NZR1|TMOD2-TMOD2_HUI	sp|Q9NZR1|TMOD2-TMOD2_HUMAN	2460800	61084000	1.53352E-05	5.38086E-05	3.50882058	up
sp|Q9P0K7|RAI14-RAI14_HUMAN	sp|Q9P0K7|RAI14-RAI14_HUMAN	85998000	3047400000	0.000535923	0.002684438	5.009001055	up
sp|Q9UHB6|LIMA1-LIMA1_HUM.	sp|Q9UHB6|LIMA1-LIMA1_HUMAN	208770000	2918500000	0.001301014	0.00257089	1.976066782	up
sp|Q9ULV4|COR1C-CORO1C_HU	sp|Q9ULV4|COR1C-CORO1C_HUMAN	1239100	150920000	7.72183E-06	0.000132945	17.21672136	up
sp|Q9UM54|MYO6-MYO6_HUMA	sp|Q9UM54|MYO6-MYO6_HUMAN	26900000	1605400000	0.000167636	0.001414188	8.436085833	up
sp|Q9Y3U8|RL36-RPL36_HUMAN	sp|Q9Y3U8|RL36-RPL36_HUMAN	3051000	58452000	1.90132E-05	5.149E-05	2.708115477	up
sp|Q9Y4I1|MYO5A-MYO5A_HUN	sp|Q9Y4I1|MYO5A-MYO5A_HUMAN	9062100	545880000	5.64732E-05	0.000480863	8.514876313	up
sp|Q9Y608|LRRF2-LRRFIP2_HUN	sp|Q9Y608|LRRF2-LRRFIP2_HUMAN	5804300	119870000	3.61713E-05	0.000105593	2.919245808	up
sp|Q9Y6R6|Z780B-ZNF780B_HUN	sp|Q9Y6R6|Z780B-ZNF780B_HUMAN	44876000	599690000	0.000279658	0.000528264	1.88895964	up
sp|O43324|MCA3-EEF1E1_HUMA	sp|O43324|MCA3-EEF1E1_HUMAN	1394800	6239100	8.69212E-06	5.49599E-06	0.632295395	down
sp|P00338|LDHA-LDHA_HUMAN	sp|P00338|LDHA-LDHA_HUMAN	21104000	93518000	0.000131516	8.23795E-05	0.626383693	down
sp|P00558|PGK1-PGK1_HUMAN;s	sp|P00558|PGK1-PGK1_HUMAN	4905500	8715600	3.05701E-05	7.67752E-06	0.251144702	down
sp|P00742|FA10-F10_HUMAN	sp|P00742|FA10-F10_HUMAN	46887000	0	0.000292191	0	0	down
sp|P01876|IGHA1-IGHA1_HUMAI	sp|P01876|IGHA1-IGHA1_HUMAN	12283000	0	7.65453E-05	0	0	down
sp|P02533|K1C14-KRT14_HUMAI	sp|P02533|K1C14-KRT14_HUMAN;CON__	1029800000	78993000	0.006417513	6.95845E-05	0.010842907	down
sp|P02538|K2C6A-KRT6A_HUMA	sp|P02538|K2C6A-KRT6A_HUMAN;CON_	1209800000	86842000	0.007539238	7.64986E-05	0.010146733	down
sp|P02545|LMNA-LMNA_HUMAI	sp|P02545|LMNA-LMNA_HUMAN	8129000	9245700	5.06583E-05	8.14449E-06	0.160772841	down
sp|P02647|APOA1-APOA1_HUMA	sp|P02647|APOA1-APOA1_HUMAN	8555100	0	5.33137E-05	0	0	down
sp|P02768|ALBU-ALB_HUMAN;C	sp|P02768|ALBU-ALB_HUMAN;CON__P0	11949000	0	7.44638E-05	0	0	down
sp|P04075|ALDOA-ALDOA_HUM	sp|P04075|ALDOA-ALDOA_HUMAN	18628000	8110600	0.000116086	7.14458E-06	0.061545565	down
sp|P04083|ANXA1-ANXA1_HUM.	sp|P04083|ANXA1-ANXA1_HUMAN	226070000	8174100	0.001408824	7.20052E-06	0.005111013	down
sp|P04264|K2C1-KRT1_HUMAN;(	sp|P04264|K2C1-KRT1_HUMAN;CON__P0	3132400000	2875200000	0.019520506	0.002532748	0.129748055	down
sp|P04792|HSPB1-HSPB1_HUMAI	sp|P04792|HSPB1-HSPB1_HUMAN	40293000	55015000	0.000251098	4.84624E-05	0.193001873	down
sp|P05787|K2C8-KRT8_HUMAN;(	sp|P05787|K2C8-KRT8_HUMAN;CON__P0	3423700	0	2.13358E-05	0	0	down
sp|P06576|ATPB-ATP5F1B_HUM/	sp|P06576|ATPB-ATP5F1B_HUMAN	3871600	9993100	2.41271E-05	8.80287E-06	0.364854547	down
sp|P06733|ENOA-ENO1_HUMAN:	sp|P06733|ENOA-ENO1_HUMAN	12515000	55210000	7.7991E-05	4.86342E-05	0.623586787	down
sp|P08727|K1C19-KRT19_HUMAI	sp|P08727|K1C19-KRT19_HUMAN;CON__	64544000	7080900	0.000402226	6.23753E-06	0.015507529	down
sp|P08779|K1C16-KRT16_HUMAI	sp|P08779|K1C16-KRT16_HUMAN;CON__	154640000	51801000	0.000963686	4.56312E-05	0.04735069	down
sp|P09211|GSTP1-GSTP1_HUMAI	sp|P09211|GSTP1-GSTP1_HUMAN	15669000	16036000	9.76462E-05	1.4126E-05	0.144665435	down
sp|P09496|CLCA-CLTA_HUMAN	sp|P09496|CLCA-CLTA_HUMAN	2770400	2111100	1.72646E-05	1.85966E-06	0.107715038	down
sp|P10809|CH60-HSPD1_HUMAN	sp|P10809|CH60-HSPD1_HUMAN	5415900	17352000	3.37508E-05	1.52853E-05	0.452886018	down
sp|P13645|K1C10-KRT10_HUMAI	sp|P13645|K1C10-KRT10_HUMAN;CON__	3992900000	1893500000	0.024882974	0.001667974	0.067032727	down
sp|P13646|K1C13-KRT13_HUMAI	sp|P13646|K1C13-KRT13_HUMAN;CON__	59281000	0	0.000369428	0	0	down
sp|P13647|K2C5-KRT5_HUMAN;(	sp|P13647|K2C5-KRT5_HUMAN;CON__P1	569340000	33170000	0.003548016	2.92193E-05	0.008235383	down
sp|P14923|PLAK-JUP_HUMAN	sp|P14923|PLAK-JUP_HUMAN	70755000	0	0.000440931	0	0	down
sp|P15924|DESP-DSP_HUMAN	sp|P15924|DESP-DSP_HUMAN	106750000	0	0.000665245	0	0	down
sp|P19013|K2C4-KRT4_HUMAN;(	sp|P19013|K2C4-KRT4_HUMAN;CON__P1	21817000	0	0.000135959	0	0	down
sp|P22528|SPR1B-SPRR1B_HUM/	sp|P22528|SPR1B-SPRR1B_HUMAN	3852200	0	2.40062E-05	0	0	down
sp|P23528|COF1-CFL1_HUMAN;s	sp|P23528|COF1-CFL1_HUMAN;sp|Q9Y28	3868600	7153300	2.41084E-05	6.3013E-06	0.261374144	down
sp|P30838|AL3A1-ALDH3A1_HUI	sp|P30838|AL3A1-ALDH3A1_HUMAN	9841200	0	6.13284E-05	0	0	down

sp|P31943|HNRH1-HNRNPH1_HU	sp|P31943|HNRH1-HNRNPH1_HUMAN;sp|	2989200	13456000	1.86281E-05	1.18533E-05	0.636313322	down
sp|P31947|1433S-SFN_HUMAN	sp|P31947|1433S-SFN_HUMAN	17781000	0	0.000110808	0	0	down
sp|P35321|SPR1A-SPRR1A_HUM	sp|P35321|SPR1A-SPRR1A_HUMAN	37640000	0	0.000234565	0	0	down
sp|P35908|K22E-KRT2_HUMAN	sp|P35908|K22E-KRT2_HUMAN	4518800000	1385400000	0.028160281	0.001220391	0.043337322	down
sp|P47929|LEG7-LGALS7_HUMA	sp|P47929|LEG7-LGALS7_HUMAN	17076000	0	0.000106414	0	0	down
sp|P58107|EPIPL-EPPK1_HUMAN	sp|P58107|EPIPL-EPPK1_HUMAN	4034500	0	2.51422E-05	0	0	down
sp|P60709|ACTB-ACTB_HUMAN	sp|P60709|ACTB-ACTB_HUMAN	70010000000	2.7872E+11	0.436288672	0.245522899	0.562753321	down
sp|P61626|LYSC-LYZ_HUMAN	sp|P61626|LYSC-LYZ_HUMAN	239260000	4927600	0.001491022	4.3407E-06	0.002911222	down
sp|P62805|H4-H4C1_HUMAN	sp|P62805|H4-H4C1_HUMAN	72052000	191640000	0.000449014	0.000168815	0.375967352	down
sp|P63104|1433Z-YWHAZ_HUMA	sp|P63104|1433Z-YWHAZ_HUMAN;sp|Q04	9150300	19056000	5.70229E-05	1.67863E-05	0.294378727	down
sp|P84085|ARF5-ARF5_HUMAN;s	sp|P84085|ARF5-ARF5_HUMAN;sp|P18085	8906700	0	5.55048E-05	0	0	down
sp|Q02413|DSG1-DSG1_HUMAN	sp|Q02413|DSG1-DSG1_HUMAN	26682000	0	0.000166277	0	0	down
sp|Q04695|K1C17-KRT17_HUMA	sp|Q04695|K1C17-KRT17_HUMAN;CON_	154360000	4474600	0.000961941	3.94165E-06	0.004097599	down
sp|Q06830|PRDX1-PRDX1_HUM	sp|Q06830|PRDX1-PRDX1_HUMAN	13928000	17809000	8.67966E-05	1.56878E-05	0.180742709	down
sp|Q08188|TGM3-TGM3_HUMAN	sp|Q08188|TGM3-TGM3_HUMAN	4104000	0	2.55753E-05	0	0	down
sp|Q13835|PKP1-PKP1_HUMAN	sp|Q13835|PKP1-PKP1_HUMAN	5153900	0	3.21181E-05	0	0	down
sp|Q14134|TRI29-TRIM29_HUMA	sp|Q14134|TRI29-TRIM29_HUMAN	3158000	0	1.968E-05	0	0	down
sp|Q5VTE0|EF1A3-EEF1A1P5_HU	sp|Q5VTE0|EF1A3-EEF1A1P5_HUMAN;sp	118310000	274340000	0.000737285	0.000241665	0.327776408	down
sp|Q71DI3|H32-HIST2H3A_HUM	sp|Q71DI3|H32-HIST2H3A_HUMAN;sp|Q1	32277000	130840000	0.000201144	0.000115256	0.573003645	down
sp|Q8TDL5|BPIB1-BPIFB1_HUM	sp|Q8TDL5|BPIB1-BPIFB1_HUMAN	23275000	0	0.000145045	0	0	down
sp|Q92841|DDX17-DDX17_HUM	sp|Q92841|DDX17-DDX17_HUMAN	2082800	8927100	1.29796E-05	7.86383E-06	0.605860783	down
sp|Q96DA0|ZG16B-ZG16B_HUM	sp|Q96DA0|ZG16B-ZG16B_HUMAN	1657500	0	1.03292E-05	0	0	down
sp|Q9UPS8|ANR26-ANKRD26_HU	sp|Q9UPS8|ANR26-ANKRD26_HUMAN	4170200	0	2.59879E-05	0	0	down
